# Toward highly efficient deep-blue OLEDs: Tailoring the multiresonance-induced TADF molecules for suppressed excimer formation and near-unity horizontal dipole ratio

**DOI:** 10.1126/sciadv.adf1388

**Published:** 2023-05-31

**Authors:** Hyung Suk Kim, Hyung Jin Cheon, Donggyun Lee, Woochan Lee, Junho Kim, Yun-Hi Kim, Seunghyup Yoo

**Affiliations:** ^1^ School of Electrical Engineering, Korea Advanced Institute of Science and Technology (KAIST), Daejeon 34141, Republic of Korea.; ^2^ Department of Chemistry and RIGET, Gyeongsang National University, Jinju 52828, Republic of Korea.

## Abstract

Boron-based compounds exhibiting a multiresonance thermally activated delayed fluorescence are regarded promising as a narrowband blue emitter desired for efficient displays with wide color gamut. However, their planar nature makes them prone to concentration-induced excimer formation that broadens the emission spectrum, making it hard to increase the emitter concentration without raising CIE *y* coordinate. To overcome this bottleneck, we here propose *o*-Tol-ν-DABNA-Me, wherein sterically hindered peripheral phenyl groups are introduced to reduce intermolecular interactions, leading to excimer formation and thus making the pure narrowband emission character far less sensitive to concentration. With this approach, we demonstrate deep-blue OLEDs with *y* of 0.12 and full width at half maximum of 18 nm, with maximum external quantum efficiency (EQE) of ca. 33%. Adopting a hyperfluorescent architecture, the OLED performance is further enhanced to EQE of 35.4%, with mitigated efficiency roll-off, illustrating the immense potential of the proposed method for energy-efficient deep-blue OLEDs.

## Teaser

MR type TADF emitters are tailored for reduced excimers and enhanced horizontal dipole orientation for efficient deep-blue OLEDs.

## INTRODUCTION

Thermally activated delayed fluorescence (TADF) molecules have been established as promising candidates for triplet-harvesting emitters in organic light-emitting diodes (OLEDs), which can lead to internal charge-to-photon conversion efficiency close to 100% (*1*). On the basis of a charge-transfer (CT)–type molecular system, and with the help of thermal energy, the spin-flip uphill transition of a dark triplet excited state to a singlet excited state occurs via reverse intersystem crossing (RISC) (*2*). For efficient room-temperature RISC in TADF systems, researchers have been mainly relying on molecular design strategies to make the spatial wave function overlap between frontier molecular orbitals (FMOs) as small as possible by combining electron donor (D) and acceptor (A) moieties into one molecule (*3*). Unfortunately, this often yields a relatively large Stokes shift and a broad emission profile, which are attributed to the substantial geometrical change between the lowest singlet state (S_1_) and the ground state (S_0_). Full widths at half-maxima (FWHMs) above 70 nm are thus observed in TADF OLEDs (*4*). This is undesirable for display applications, which generally require saturated primary colors for wide color gamut.

In this regard, multiresonance (MR) TADF emitters, called “ν-DABNA,” have recently been proposed as alternatives that can overcome the large FWHM of typical D-A CT-type TADF emitters. Separating FMOs in the boron-centered azatriangulene-like backbone brings about the MR effect between boron and nitrogen atoms (*5*), which in turn reduces the vibrational relaxation of molecules in each electronic state. On the basis of π extension of the so-called DABNA structure, ν-DABNA used as MR-TADF emitters enabled an OLED device with highly efficient pure-blue emission, a maximum external quantum efficiency (EQE) of ~34%, and narrow FWHM of 18 nm (*6*). In this MR-TADF molecule, FMOs localized at individual atoms resulted not only in small Δ*E*_ST_ but also in strong oscillator strength (*f*_osc_) (*7*), which is in contrast to conventional D-A–type CT TADF emitters, which are subject to a trade-off in which the decrement of Δ*E*_ST_ with small spatial overlap of FMOs leads to lowered *f*_osc_ and thus reduced photoluminescence quantum yield (PLQY).

Nevertheless, the notable properties of ν-DABNA turned out to be valid only when the emission layer (EML) had a very low doping concentration. This low-concentration requirement stems from the planar molecular π framework of ν-DABNA, which makes this material prone to excimer formation via π-π intermolecular interaction, which could then yield the appearance of a shoulder peak at a longer wavelength than that of the main emission peak. For various host layers, it was shown that this shoulder peak was noticeable even with 0.1% concentration of ν-DABNA and that it already became quite significant at doping concentration as low as 1%. This shoulder peak could be problematic as its influence could overshadow the advantage of a narrow FWHM, yielding a higher Commission Internationale de l'éclairage (CIE) *y* coordinate than it would otherwise achieve (*8*). Furthermore, OLEDs adopting hyperfluorescent (HF) architecture with EMLs doped with ν-DABNA as end dopants showed efficiency that was not equal to what HF architecture can offer; this limitation was also attributed to excimer formation, a major source of exciton quenching, which significantly appeared even at very low doping concentration of 1 weight % (wt %). This low-doping requirement can further limit the effective rate constant of Förster resonant energy transfer (FRET) between host and dopant in HF-type OLEDs, leading to disadvantages in attempts to further enhancing the efficiency as well as keeping the efficiency roll-off low at high brightness condition (*9*–*10*).

**Fig. 1.**
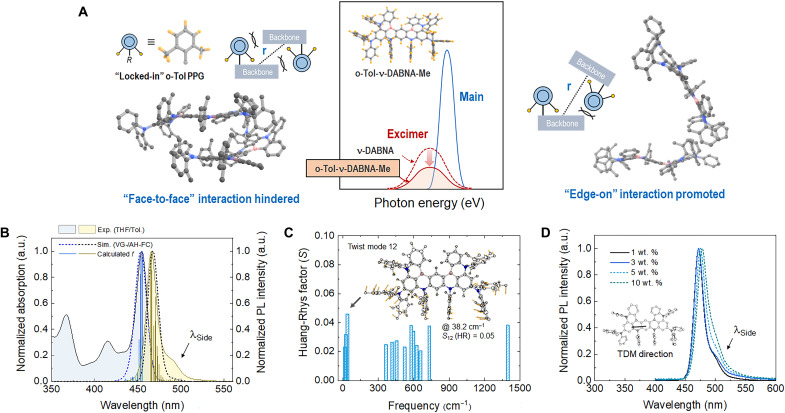
Photophysical characteristics. (**A**) Schematic illustration of the proposed design concept for *o*-Tol-ν-DABNA-Me molecule, the structure of which is shown as an inset in the middle. By introducing PPGs locked in sterically orthogonal orientation via methyl substitution, face-to-face interaction (shown on the left) leading to excimer formation is hindered, while the relative presence of edge-on interaction (shown in the right) becomes more prominent. Main MR-TADF and excimer contribution to typical emission spectra are shown schematically in the middle. (**B**) Absorption spectrum (blue) of *o*-Tol-ν-DABNA-Me in THF solution (0.01 mM) and fluorescence spectrum (yellow) measured with a solution in toluene. Simulated absorption spectrum (dotted line) obtained by VG-FC analysis and scaled emission spectrum (dotted line) modeled by AH-FC method are also shown. The blue sticks correspond to the oscillator strength (*f*) (see also fig. S26). (**C**) HR factor (*S*_i_) involved in reorganization energy (λ_S_) for *o*-Tol-ν-DABNA-Me system, in which the cutoff was 0.02 (dimensionless). Inset, schematic representation of “twist mode 12” in *o*-Tol-ν-DABNA-Me. (**D**) Steady-state PL spectra of *o*-Tol-ν-DABNA-Me doped in DBFPO host. Inset, TDM direction for *o*-Tol-ν-DABNA-Me from the transition: S_1_ → S_0_ − (*X*, *Y*, *Z*) = (−3.91, −0.10, 0.17) − lies along with the five-membered ring plane (i.e., long axis of molecule). a.u., arbitrary units.

To solve these issues, we propose an MR-type TADF emitter called “*o*-Tol-ν-DABNA-Me” (see table S1 and figs. S1 to S24 for details on their synthetic routes). Its peripheral phenyl groups (PPGs) being locked in sterically orthogonal orientation via methyl substitution, the proposed approach provides a structural foundation for significant reduction in intermolecular face-to-face π-π interaction, as described in Fig. 1A. A comprehensive set of calculation methods, including quantum mechanical and molecular dynamic (MD) simulations, is used to ensure that the proposed structural modification indeed helps achieve the goal while keeping the major benefits of ν-DABNA intact. The feasibility of the proposed approach in actual devices is tested with OLEDs adopting HF-based architecture as well as OLEDs in conventional host:guest TADF configuration. These OLEDs based on *o*-Tol-ν-DABNA-Me are shown to exhibit electroluminescence (EL) spectra with a far reduced side peak compared to those reported for ν-DABNA–based OLEDs, even for emitter concentrations as high as 5 wt %, illustrating the validity of the proposed approach.

In addition, our MD simulation–based study reveals that these sterically orthogonal PPGs added to the ν-DABNA backbone can cause *o*-Tol-ν-DABNA-Me to form preferred horizontal transition dipole orientations (TDOs) during deposition; the experimental results indicate the ratio of horizontal TDOs (Θ_h_) in *o*-Tol-ν-DABNA-Me thin films to be as high as 95%, consistent with the theoretical prediction. Because of to this very high Θ_h_, as well as to the excimer suppression that extends the range of allowable emitter concentration, the proposed OLEDs exhibit very high EQE of ca. 32% and a relatively low CIE *y* coordinate of 0.12, illustrating the excellent potential of *o*-Tol-ν-DABNA-Me as pure blue emitters in OLEDs. Upon adopting an HF architecture, the OLED performance is further enhanced to EQE of 35.4% (56.3% with a half-ball lens) with mitigated efficiency roll-off. Analysis of exciton dynamics performed at steady state showed that excitonic annihilation processes are reduced at a given current density for OLEDs in the HF architecture with an emitter concentration of 3 wt %, explaining the origin of the mitigated roll-off. We believe that our study provides deeper insight into the design of sterically hindered MR-TADF molecules for highly efficient deep-blue OLEDs and provides a direction for future synthetic development to get the most out of DABNA analog systems.

## RESULTS

### Photophysical properties of *o*-Tol-ν-DABNA-Me and its intermolecular interaction

Figure 1A shows the schematic illustration of the proposed design concept for *o*-Tol-ν-DABNA-Me showing face-to-face arrangement and edge-on arrangement as its representative molecular arrangements (refer to tables S2 and S3 and figs. S25 to S27 for calculated electronic properties). It is based on the ν-DABNA backbone as a main electronic transition site, but its PPGs are modified in the form of sterically hindered *o*-tolyl groups as shown in Fig. 1A. With the methyl substitution in the proposed positions, the PPGs in this molecule are orthogonally locked-in and have a far less degree of freedom in movement than the case of conventional ν-DABNA, hindering adjacent molecules from getting too close to one another face-to-face so that excimer formation can be discouraged.

When molecular engineering is done for a certain purpose via modification of peripheral groups, it is important to keep the beneficial characteristics of the parent molecule intact. In the present system, the normalized absorption spectrum of *o*-Tol-ν-DABNA-Me dissolved in tetrahydrofuran (THF) exhibits electronic transitions inherent to the ν-DABNA core (*8*). The strong absorption band at 455 nm (log ε = 5.01, ε: molar extinction coefficient, M^−1^ cm^−1^) corresponds to the main electronic transition for S_0_ → S_1_ transition (Fig. 1B). The spectral shape, having FWHM of 11 nm, coincides well with the one simulated by vertical gradient Franck-Condon (VG-FC) analysis (*11*–*12*). Mirrored from this first absorption band, the photoluminescence (PL) spectrum exhibits a sharp symmetric emission centered at 466 nm with FWHM of 14 nm (Fig. 1B), confirming that the molecular engineering done in this work does preserve the desirable properties of the ν-DABNA core. Similar to the case of absorption, the PL spectrum shows good agreement with the spectral shape predicted by the adiabatic Hessian Franck-Condon (AH-FC) method for S_1_ → S_0_ transition (*11*–*12*). As shown in fig. S28, the room-temperature PL and low-temperature PL curves look very similar, and the solvatochromic effect is less visible in the absorption and emission profiles of *o*-Tol-ν-DABNA-Me. These results indicate that S_1_ and T_1_ have similar MR features, resulting in a very small ∆*E*_ST_ of ~0.01 eV; also, the solvent-induced dipole moment change is orthogonal to the transition dipole moment (TDM) vector of *o*-Tol-ν-DABNA-Me, which lies along the five-membered ring plane (i.e., the long axis of the molecule), as shown in the inset of Fig. 1D and in fig. S29 (see also figs. S30 and S31 and table S4 for a summary of photophysical properties of o-Tol-ν-DABNA-Me).

The small Stokes shift of only 11 nm, as well as the low FWHM values, can be attributed mainly to the rigid π-conjugated framework, which minimizes the change in structural displacement between S_0_ and S_1_ electronic states and reduces molecular vibration in each of the electronic states. Consistent with this observation, the reorganization energies of *o*-Tol-ν-DABNA-Me for its S_1_ (λ_S_) and T_1_ (λ_T_) states have been estimated to be as low as 0.03 and 0.04 eV, respectively, from the potential energy surface curves (*13*). However, modification of peripheral groups can often change the molecular vibrational behavior in such a way that Stokes shift and FWHM increase (*8*). A detailed analysis done under harmonic oscillator approximation (*14*) to find the components influencing λ_S_ of *o*-Tol-ν-DABNA-Me showed that the largest Huang-Rhys (HR) factor (*S*_i_) (i.e., the electron-vibration coupling) corresponds to the vibrational twist mode 12 (38.2 cm^−1^) associated with the proposed PPGs (see the inset of Fig. 1C). Nevertheless, even the highest HR factor (*S*_12_) turns out to be merely 0.05, and the total HR factor for all the vibrational modes involved ($=\sum_{i=1}^{504}S_{i}$) is estimated to be as small as 1.07 (see Fig. 1C and fig. S32). Given that the representative D-A–type TADF molecule, 1,2,3,5-tetrakis(carbazol-9-yl)-4,6-dicyanobenzene (4CzIPN) (*1*), shows a large vibrational mode (e.g., *S* > 13 for torsional motion at low frequency) for the decay process from S_1_ to S_0_ state, the relatively low values estimated for *o*-Tol-ν-DABNA-Me suggest that its major nonradiative recombination channel is effectively restricted by the steric hindrance introduced by our finely tuned PPG design (*15*–*17*). A one-to-one photophysical comparison study done between ν-DABNA and the proposed *o*-Tol-ν-DABNA-Me also supports the effectiveness in our design strategy (refer to the “Comparison study for *o*-Tol-ν-DABNA-Me vs. ν-DABNA” section in the Supplementary Materials for details).

**Fig. 2.**
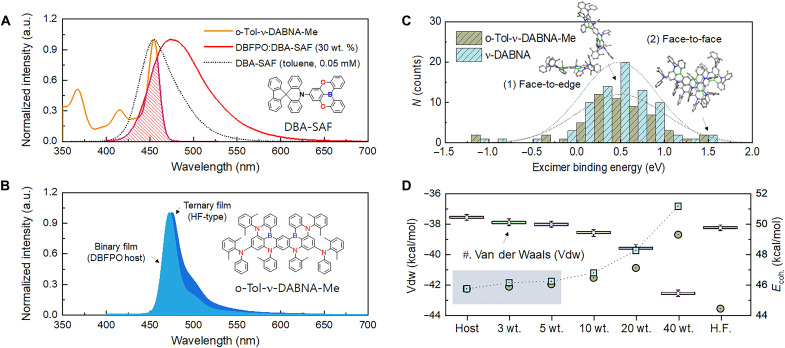
Intermolecular interaction model. (**A**) Normalized absorption spectrum of *o*-Tol-ν-DABNA-Me in THF at concentration of 10^−5^ M and steady-state PL spectra of 30 wt % DBA-SAF doped DBFPO film and DBA-SAF in toluene (0.05 mM). (**B**) Steady-state PL spectra for 3 wt % *o*-Tol-ν-DABNA-Me:DBFPO binary film and for *o*-Tol-ν-DABNA-Me (3 wt %):DBA-SAF (30 wt %):DBFPO ternary film. (**C**) Excimer binding energy distributions for ν-DABNA and *o*-Tol-ν-DABNA-Me. (**D**) Calculated nonbonding energy (*E*_coh_) and separated van der Waals (Vdw) energy (kcal/mol) for each individual system integrated from 20-ns converged frames.

While one can now be assured that the proposed modification leads to neither a considerable change in vibrational behavior nor a compromise in the main feature of its luminescence, it is important to check if it can indeed suppress or reduce the chance of excimer formation. First, one may note that there is a shoulder peak (λ_Side_) around 470 to 500 nm in the PL spectrum for both solution and film states; it is this peak that is attributed to excimer formation resulting from intermolecular interactions between π-rich ν-DABNA derivatives (*8*). With the doping concentration change of 3 to 5 wt % in *o*-Tol-ν-DABNA-Me doped within a host layer of 2,8-bis(diphenyl-phosphoryl)dibenzo[b,d]furan (DBFPO) (“binary” film), contribution from λ_side_ to the overall PL spectra becomes more apparent, making the FWHM and the CIE *y* coordinate increase (Fig. 1D and fig. S33, A to C). When it is co-deposited with 10-(5,9-dioxa-13b-boranaphtho[3,2,1-de]anthracen-7-yl)-10*H*-spiro[acridine-9,9′-fluorene] (DBA-SAF), the newly designed boron-based D-A–type CT emitter (refer to fig. S1 and Fig. 2A), a TADF assistant dopant for HF architecture (“ternary” film), shows a shoulder peak higher and broader than that of the binary (BN) film; this is because emission spectrum from DBA-SAF itself can still be mixed-in due to efficient yet limited energy transfer to the end emitter. Nevertheless, it may be emphasized that the shoulder peaks in both binary and ternary films are much less apparent than those reported previously for ν-DABNA (*9*); this is particularly so when one considers the relatively high doping concentration of *o*-Tol-ν-DABNA-Me (Fig. 2B).

To elucidate the origin of the subdued shoulder peak for the films with *o*-Tol-ν-DABNA-Me, we carried out MD simulation of isothermal-isobaric (i.e., the system in contact with a thermostat at temperature *T* and a barostat at pressure *p*, *NpT* ensemble class for both binary and ternary EML systems. A molecular simulation cubic box under the periodic boundary condition was built by populating molecules (*N* = 1024 molecules) using the self-avoiding random walk algorithm implemented in Desmond (Schrödinger Inc.) (*18*–*19*), with a uniform probability distribution for the rotational bonds included in the molecules under study. By extracting the excimer clusters with the condition of center-of-mass (COM) distance < 15 Å from the converged frame at 100 ns (20 wt % doped DBFPO host system), we identified that the number of excimer formations for *o*-Tol-ν-DABNA-Me (55 sets) is smaller by ca. 30% than that for ν-DABNA (78 sets) at the same condition. Using a posteriori-corrected functional B3LYP-MM that considers noncovalent interactions such as van der Waals interactions, and with basis set superposition error (BSSE) corrected, the excimer binding energy distribution for each molecule was obtained as shown in Fig. 2C (*20*–*21*). Note that the major portion of the excimer configuration is mainly in face-to-edge rather than face-to-face form. The modeling results indicate that the numbers of both configurations are reduced in *o*-Tol-ν-DABNA-Me, illustrating the critical role of rigidified PPGs in hindering excimer formation.

Van der Waals energies increase gradually with the doping ratio, in contrast to the case of electrostatic energies, which decrease with the doping ratio (see Fig. 2D). This is because dipole-induced interactions are mainly caused by the presence of ν-DABNA derivatives, in contrast to the electrostatic energies, which originate mainly from the polar DBFPO host (refer to table S5 for summary of simulation results). The increase in van der Waals energy with the doping ratio is clearly less significant in the *o*-Tol-ν-DABNA-Me case (−57.02 kcal/mol at neat) than in the case of ν-DABNA (−61.55 kcal/mol). In short, introduction of sterically hindered PPGs in *o*-Tol-ν-DABNA-Me reduces, though it does not completely suppress, excimer formation, yielding a far more reduced side peak than that of ν-DABNA system at each doping concentration.

### Promotion of molecular anisotropy

**Fig. 3.**
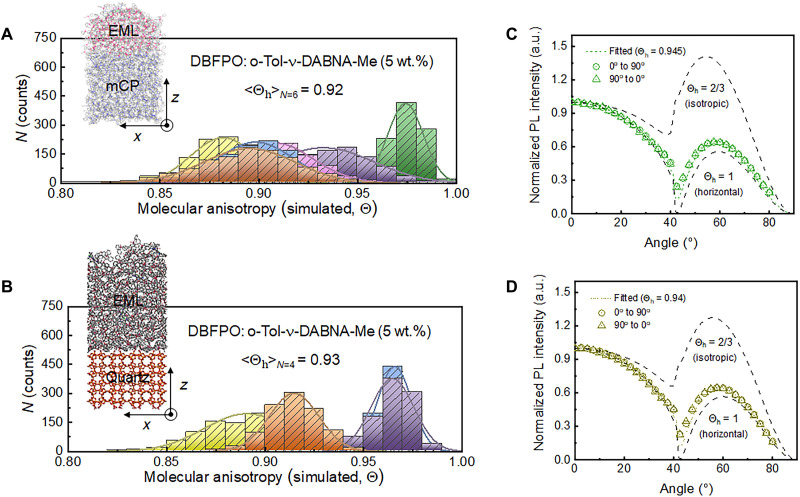
Horizontal TDM orientation ratio (Θ_h_). Molecular deposition simulation results for (**A**) DBFPO:*o*-Tol-ν-DABNA-Me (5 wt %) deposited on an amorphous mCP layer and (**B**) the same deposited on a quartz substrate. Angular-dependent *p*-polarized PL profiles of (**C**) DBFPO:*o*-Tol-ν-DABNA-Me (3 wt %) (the binary film) and of (**D**) DBFPO:DBA-SAF (30 wt %):*o*-Tol-ν-DABNA-Me (3 wt %) (the ternary film).

In addition to reduction of excimer formation, the sterically hindered PPGs provide an additional benefit by retaining the horizontal TDO of the DABNA-based ribbon core, which is known to be critical for efficiency enhancement. Figure 3A shows MD simulation results obtained by dropping *o*-Tol-ν-DABNA-Me and DBFPO molecules onto 1,3-bis(*N*-carbazolyl)benzene (mCP) substrate under vacuum environment by thermal equilibration at 300 K. MD simulation was done by running *NVT* (i.e., the number of particles *N* and volume *V* remain fixed in the system) ensemble class for 3 ns with a time step of 2.0 fs; trajectory of system was recorded every 10 ps (*19*, *22*). During this deposition protocol, we restricted the translational motion of mCP molecules to prevent the potential change from system drift, particularly when the doped molecules impart an additional momentum in the (−) *z* direction. The statistical average of horizontal TDM orientation ratios <Θ_h_>_*N*=6_ is as high as 0.92, indicating that the interaction of *o*-Tol-ν-DABNA-Me molecules with DBFPO host molecules on mCP substrates during film deposition allows them to have a very high degree of preferred horizontal TDM orientation. In the simulation performed on quartz instead of mCP as underlying substrate, the TDM orientation of *o*-Tol-ν-DABNA-Me for the same EML configuration remained almost unchanged, suggesting weak or little dependence of its TDM orientation on underlying layers (Fig. 3B).

Considering that the TDM of *o*-Tol-ν-DABNA-Me is in the plane of the DABNA ribbon along the long axis (see fig. S29 and the inset of Fig. 1C), the high Θ_h_ indicates that the presence of sterically hindered PPGs causes the long axis of DABNA-based ribbon core to be oriented perpendicular to the *z* direction in randomly distributed DBFPO hosts due to nonbonding interactions (i.e., host-guest interaction). From an angle-dependent *p*-polarized PL profile (refer to figs. S34 and S35), Θ_h_ of the proposed material is estimated to be very high, at 0.945 ± 0.005, showing quantitative agreement with predictions made by MD simulation (Fig. 3C). To see whether this high degree of TDM orientation is achieved only with a specific host material, *o*-Tol-ν-DABNA-Me was doped into bis[2-(diphenylphosphino)phenyl]ether oxide (DPEPO) and into [1-(1,4-dicyanocarbazolyl)-3-carbazolylbenzene] (*p*-dCNmCP), because polar host materials can anchor target materials by a series of electrostatic energies [−P=O^δ−^ in DPEPO or −CN^δ−^ in *p*-dCNmCP with H(PPG)], as presented in the analysis of electrostatic potentials (fig. S36). In each of the binary films, the value of Θ_h_ turns out to be in the range of 0.94 to 0.95. Furthermore, the ternary film for HF architecture design also exhibited a Θ_h_ of 0.95 (Fig. 3D). These results illustrate near-universal realization of horizontal TDM orientation obtained via molecular design in which the PPGs proposed in this work maintain the molecular arrangement highly anisotropic by a series of dipole-induced van der Waals interactions between our target molecule and surrounding host molecules, rather than by Coulomb interactions.

Last, we have explored the potential of our target molecule as a pure-blue emitter in OLEDs. The device configurations we designed were based on (i) binary (conventional) EMLs (3.0 and 5.0 wt % doped into DBFPO host; Dev. A and Dev. B, respectively) and (ii) ternary (HF) EMLs (*x* wt % *o*-Tol-ν-DABNA-Me: 30 wt % DBA-SAF: DBFPO host, where *x* = 1.5 and 3.0 for Dev. C and Dev. D, respectively) (see Table 1 and figs. S37 to S42 for summary of detailed device characteristics).

**Table 1.** Characteristics of tested OLED devices.

Device*	Type/dopant concentration (wt %)	λ_EL_ (nm)	Voltage (V)^†^	EQE (%)^‡^	Power efficiency (lm/W)^§^	Current efficiency (cd/A)^║^	λ_FWHM_ (nm)	CIE (*x*, *y*)
Dev. A	Bin./3.0	472	3.4/6.4	33.1/17.7	26.3/7.7	31.1/16.7	18	(0.11, 0.12)
Dev. B	Bin./5.0	473	3.4/6.6	28.8/15.2	29.5/8.0	34.9/18.4	20	(0.12, 0.17)
Dev. C	HF/1.5	472	3.1/6.4	32.9/22.5	36.5/11.7	38.1/26.1	19	(0.13, 0.16)
Dev. D	HF/3.0	472	3.4/6.6	35.4/25.0	33.9/12.4	38.2/27.0	18	(0.12, 0.15)
Dev. A′^¶^	Bin./3.0	472	3.4/5.4	55.6/40.2	47.9/21.8	75.2/54.3	18	(0.12, 0.13)
Dev. D′^¶^	HF/3.0	472	3.1/5.6	56.3/44.4	57.5/26.0	97.0/76.6	18	(0.12, 0.15)

*ITO (70 nm)/MoO_3_ (4 nm)/TAPC (40 nm)/TCTA (10 nm)/mCP (10 nm)/EML (25 nm)/DBFPO (5 nm)/TPBi (35 nm)/LiF (1 nm)/Al (100 nm), with the binary film (3.0, 5.0 wt % doped DBFPO host) for Dev. A and Dev. B and the ternary film (1.5, 3.0 wt % *o*-Tol-ν-DABNA-Me: 30 wt % DBA-SAF: DBFPO host) for Dev. C and Dev. D.

†Applied voltage at luminance of 1 and 1000 cd/m^2^.

‡EQE: maximum, then values at 1000 cd/m^2^.

§Power efficiency: maximum, then values at 1000 cd/m^2^.

║Current efficiency: maximum, then values at 1000 cd/m^2^.

¶With a half-ball lens.

**Fig. 4.**
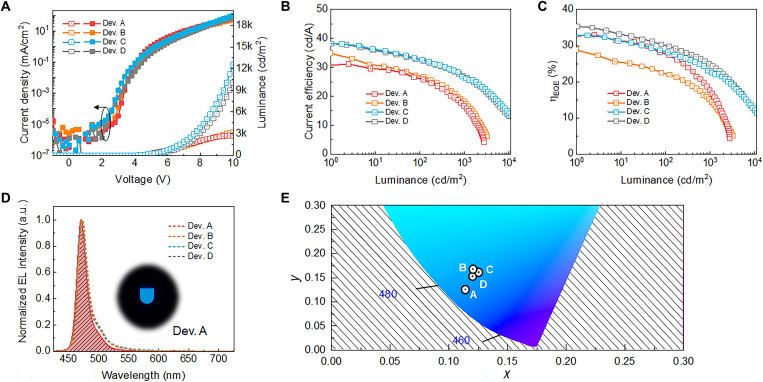
OLED performance. (**A**) *J*-*V*-*L* characteristics and (**B**) CE-luminance curves of tested OLEDs. (**C**) EQE-luminance curves of tested OLED devices. (**D**) EL spectra for Dev. A (3 wt % doped, binary), Dev. B (5 wt% doped, binary), Dev. C (1.5 wt % doped, HF), and Dev. D (3 wt % doped, HF). Inset image: photograph of Dev. A. (**E**) Color coordinates plotted in CIE 1931 diagram.

Figure 4 (A to E) shows the current density-voltage-luminance (*J-V*-*L*), EQE-luminance, current efficiency (CE)–luminance, EL spectra, and CIE chromaticity for devices under study. Devices with 3 wt % *o*-Tol-ν-DABNA-Me doped into DBFPO host (Dev. A) exhibit maximum EQE and CE of 33.1% and 31.1 cd/A, respectively. These high values of EQE are attributed partly to the high degree of horizontal TDM orientation of *o*-Tol-ν-DABNA-Me, as well as to the high PLQY and fast RISC. In devices using HF architecture with DBA-SAF as an assistant dopant, the maximum EQE of OLEDs improved to 35.4% (56.3% with a half-ball lens) for 3 wt % *o*-Tol-ν-DABNA-Me (Dev. D); this was because the optimal HF architecture led to a sufficient FRET from DBA-SAF to *o*-Tol-ν-DABNA-Me (see the “Hyperfluorescent (HF) design” section in the Supplementary Materials and tables S6 to S8 as well as figs. S43 and S44 for detailed FRET analysis done for present HF scheme).

The FWHM and CIE *y* coordinate for the EL spectral emission of Dev. A are 18 nm and 0.12, respectively, corresponding to the blue index (the color coordinate corrected CE) of 259 cd/A (*2*, *6*). The achieved FWHM and *y* coordinate are comparable to previously reported values for OLEDs based on 1 wt % ν-DABNA emitters (*6*). Considering the PL of ν-DABNA doped into similar host materials, which showed strong side emission peaks when doping concentration was larger than 1% (*8*), the observed small *y* coordinate at the dopant concentration of 3 wt % supports the validity of the proposed approach based on steric hindrance induced with PPGs.

In cases Dev. C and Dev. D, the EL spectra result partly from the excited states of sensitizers that do not undergo energy transfer to *o*-Tol-ν-DABNA-Me; this emission from sensitizers, though slight, introduced a long tail for λ > 500 nm and thus increased the *y* coordinates to 0.16 and 0.15, respectively. Nevertheless, increases in excimer-related side emission are shown to be minimal in the present systems, even with relatively high dopant concentration (and even for binary EML composition with 5 wt % dopant concentration; Dev. B), keeping the *y* coordinates relatively low. This low sensitivity of the EL spectra to the end dopant concentration extends the range of dopant concentrations that can be adopted without concern on excessive excimer formation and provides a higher degree of freedom to find optimal device structure, for example, for enhanced FRET in the HF system via increased end-dopant concentrations; the corresponding high EQE value in the proposed HF-based OLEDs can be regarded as a clear step-up from the EQE of the previous reports made for HF OLEDs with low end-dopant concentration (*8*). To support the effectiveness of PPG design, we have tried out a one-to-one comparison between ν-DABNA and the proposed *o*-Tol-ν-DABNA-Me using the same type (i.e., BN and HF type) and the same concentration of 3 wt %, allowing for a more straightforward comparison. The detailed information on the device performances (*o*-Tol-ν-DABNA-Me versus ν-DABNA) is summarized in the Supplementary Materials for “Comparison study for *o*-Tol-ν-DABNA-Me versus ν-DABNA” section (tables S9 and S10 and figs. S45 to S54). We conducted further investigations on the photostability of o-Tol-ν-DABNA-Me in comparison to that of the control, ν-DABNA (fig. S55).

**Fig. 5.**
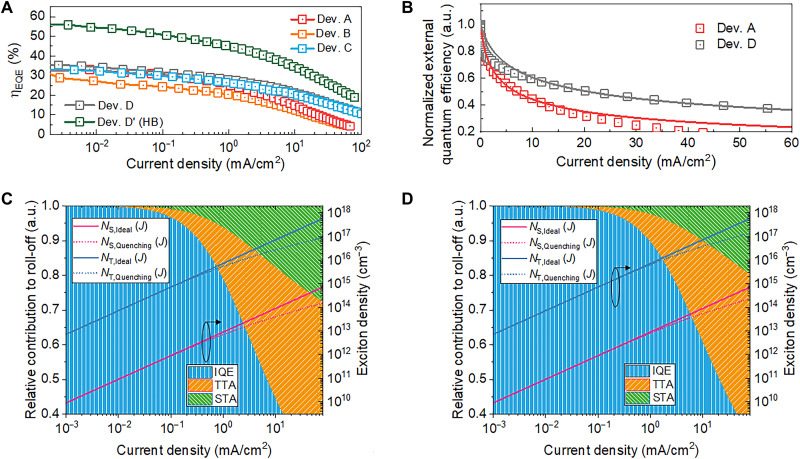
Roll-off characteristics. (**A**) *J*-EQE curves for tested OLED devices. (**B**) Quantification of roll-off in ν-DABNA derivative system. The solid line corresponds to calculated *J*-EQE model based on biexcitonic TTA and STA processes. (**C** and **D**) Simulated relative contributions of TTA-based (orange) and STA-based quenching (olive) to efficiency roll-off as a function of current density for (C) Dev. A and (D) Dev. D, respectively. The remaining portion (blue) corresponds to the fraction of excitons that can make radiative recombination and thus corresponds to the achievable internal quantum efficiency (IQE). The line curves show the number density of S_1_ (red) and T_1_ (blue) excitons for the case without quenching (ideal; solid line) and for the case with quenching considered (dashed line), respectively.

While the max EQE values of OLEDs based on ternary EMLs are comparable with those of OLEDs based on binary EMLs, it is the efficiency roll-off behavior that gives a clear edge to HF-based OLEDs. As shown in Fig. 4 (B and C), the ratio of EQE measured at 1000 nits to that measured at 1 nit is 70.6% for Dev. D, while it is only 53.5% for Dev. A. If the ratio is estimated for 2500 nits, the relative difference becomes even more severe at 58.5% for Dev. D and 21.1% for Dev. A. This roll-off behavior is in good agreement with the biexcitonic model for triplet-triplet annihilation (TTA) or singlet-triplet annihilation (STA), which have been regarded as major roll-off components in TADF-based OLEDs (*23*–*24*). From analysis of exciton dynamics, the rate constant for STA (*k*_STA_) decreases 3.3-fold from Dev. A (4.0 × 10^−9^ cm^3^ s^−1^) to Dev. D (1.2 × 10^−9^ cm^3^ s^−1^), and the rate constant of TTA (*k*_TTA_) decreases 2.5-fold from 2.5 × 10^−11^ cm^3^ s^−1^ for Dev. A to 1.0 × 10^−11^ cm^3^ s^−1^ for Dev. D. In the modeling results presented in Fig. 5 (C and D), it can be seen that the relative contributions of TTA (orange hatched region) and STA (olive hatched area) are smaller in Dev. D than in Dev. A for a given *J*, leading to the reduced gap between the actual and ideal singlet or triplet exciton density and thus reduced roll-off in Dev. D. Given that the experimentally determined *k*_RISC_ was 3.21 × 10^5^ s^−1^ @ 3 wt % doped DBFPO host (binary film), the average number (#*n*) of ISC ↔ RISC spin cycles (S_1_ → T_1_ → S_1_, defined as a unit cycle) (*25*–*26*) is estimated to be approximately 0.19. This low spin cycle value corresponds to an efficient consumption of excited states via S_1_ → S_0_ immediately after formation of (or conversion into) S_1_, which is regarded as essential for mitigated efficiency roll-off, as discussed previously (*13*).

## DISCUSSION

In this work, *o*-Tol-ν-DABNA-Me was explored as a MR-TADF molecule for efficient deep blue emitters. The proposed methyl substitution at the ortho-tolyl position of PPGs in ν-DABNA was shown to provide steric hindrance that reduced intermolecular van der Waals interactions without undermining the major benefits of ν-DABNA, which include narrow FWHM, small Stokes shift, and high PLQY. The reduced intermolecular interactions in the proposed molecules tend to decrease the chance of excimer formation at a given dopant concentration compared to as-is ν-DABNA, extending the range of dopant concentration that can be used in OLEDs, making device optimization easier without worry of excessive formation of excimers, which could make the CIE *y* coordinate larger than the value corresponding to the pure S_1_ → S_0_ emission peak. With 3.0 wt % *o*-Tol-ν-DABNA-Me doped into DBFPO host, OLEDs exhibited maximum EQE of 33.1% as well as CE and *y* coordinate of 31.1 cd/A and 0.12, respectively, which corresponds to blue index as high as 259 cd /A. By using HF architecture with DBA-SAF, a newly designed boron-based CT-type TADF molecule, as an assistant dopant in the ternary EML, we were able to improve the maximum EQE of OLEDs to 35.4% (56.3% with a half-ball lens) and mitigate the efficiency roll-off. Comprehensive spectroscopic and modeling study revealed that the steric hindrance introduced in the proposed molecular design reduced excimer formation while retaining the beneficial characteristics of ν-DABNA, including its high degree of horizontal TDM orientation and pushing the emission peak wavelength toward a deeper blue region. We believe that the results presented in this work provide deeper insight into the molecular design that needs to be done to get the most out of DABNA analog systems and establish a foundation for future synthetic approaches to the development of efficient deep-blue emitters.

## Supplementary Materials

### This PDF file includes:

Supplementary Text Figs. S1 to S55 Tables S1 to S10 References
