# Supplementary material for: Toward highly efficient deep-blue OLEDs: Tailoring the multiresonance-induced TADF molecules for suppressed excimer formation and near-unity horizontal dipole ratio
Source: Sci Adv. 2023 May 31;9(22):eadf1388. doi: 10.1126/sciadv.adf1388 (PMC10413681; doi:10.1126/sciadv.adf1388)
Supplement: Supplementary file 1 — Supplementary Text Figs. S1 to S55 Tables S1 to S10 References [file sciadv.adf1388_sm.pdf]

Science Advances
AAAS

Supplementary Materials for

Toward highly efficient deep-blue OLEDs: Tailoring the multiresonance-induced TADF molecules for suppressed excimer formation and near-unity horizontal dipole ratio

Hyung Suk Kim *et al.*

Corresponding author: Yun-Hi Kim, ykim@gnu.ac.kr; Seunghyup Yoo, syoo.ee@kaist.edu

Sci. Adv. **9**, eadf1388 (2023)
DOI: 10.1126/sciadv.adf1388

This PDF file includes:

Supplementary Text
Figs. S1 to S55
Tables S1 to S10
References

I. Experimental

Solvents. All materials associated with synthesis were purchased from Sigma-Aldrich, Tokyo Chemical Industry (TCI) or Alfa Aesar and were used without additional purification. The compounds 7-bromo-5,9-dioxa-13b-boranaphtho[3,2,1-*de*]anthracene (DBA-Br), (*27*) 10*H*-spiro[acridine-9,9'-fluorene] (*28*) were synthesized according to the previous procedures. Anhydrous toluene was purchased Aldrich. Common organic solvents were purchased from Daejung Chemical & Metal Co. Ltd.

Materials. We used Molybdenum oxide (MoO_x) as a hole injection layer (HIL) between an Indium-tin oxide (ITO) layer and a hole transport layer (HTL). And, 1,1-Bis[(di-4-tolylamino)phenyl]cyclohexane (TAPC) was utilized for HTL. For the balanced hole injection, Tris(4-carbazoyl-9-ylphenyl)amine (TCTA) and 1,3-Bis(*N*-carbazolyl)benzene (mCP) were used for the consecutive HTL. We took a series of host materials, 2,8-Bis(diphenyl-phosphoryl)dibenzo[b,d]furan (DBFPO), Bis[2-(diphenylphosphino)phenyl] ether oxide (DPEPO), and [1-(1,4-dicyanocarbazolyl)-3-carbazolylbenzene] (*p*-dCNmCP), to scrutinize molecular anisotropy with host dependence. Herein, we mainly used DBFPO host for the emission layer (EML). And, 10-(5,9-dioxa-13b-boranaphtho[3,2,1-*de*]anthracen-7-yl)-10*H*-spiro[acridine-9,9'-fluorene] (DBA-SAF) was designed as an assistant dopant. And, as a terminal multi-resonance charge-transfer (MR-CT) dopant, N^7,N^7,N^7,N^{13},5,9,11,15-tetrakis(2,6-dimethylphenyl)-9,11-bis(*o*-tolyl)phenyl)-5,9,11,15-tetrahydro-5,9,11,15-tetraaza-19b,20b-diboradinaphtho[3,2,1-*de*:1',2',3'-*jk*]pentacene-7,13-diamine (*o*-Tol-*ν*-DABNA-Me), was newly synthesized for the study. To prevent carrier overflow from EML to electron transport layer (ETL), DBFPO was used as the hole blocking layer (HBL) and 2,2′,2′′-(1,3,5-Benzinetriyl)-tris(1-phenyl-1-*H*-benzimidazole) (TPBi) works as an ETL in this study. And, lithium fluoride (LiF) and aluminum (Al) were used as an electron injection layer (EIL), and cathode, respectively. And, other organic electronic materials, excluding the materials; DBFPO, *p*-dCNmCP, DBA-SAF, and, o-Tol-ν-DABNA-Me, were purchased from Alfa Aesar, Nichem Fine Tech. Co. Ltd, and Luminescence Tech. Corp.

Optoelectronic Characterization of Materials. The absorption spectra for target molecules dissolved in the anhydrous tetrahydrofuran (THF) solution were obtained by UV-vis/IR spectroscopy (PerkinElmer, LAMBDA-900). We recorded the room temperature photoluminescence (RTPL) and low temperature (LTPL) spectra (at 77K) of target materials (i.e., o-Tol-ν-DABNA-Me and DBA-SAF) dissolved in the solution using a PL spectrofluorometer (JASCO, FP-8500) and luminescence spectrophotometer for the cross-check (PerkinElmer, LS-50B). The RTPL spectra of the solid thin film deposited on a quartz substrate (UV grade, Corning 0F 7980 HPFS) were obtained by PL spectrofluorometer (JASCO, FP-8500). We then studied transient photoluminescence (Tr-PL) behaviors of a series of each system (e.g., solution or solid-state) in N_2 purged system. The Tr-PL decay profiles @ RT were recorded with a time-correlated single-photon counting (TCSPC) method using an LED at 340 nm (or 280 nm) and a Quantaurus-Tau fluorescence lifetime measurement system (Hamamatsu Photonics, C11367-03). The absolute PL quantum yields (PLQY) for the target materials at each phase (e.g., solution and

solid-state) were measured by a Quantaurus-QY C11347 (Hamamatsu Photonics) system equipped with an integrating sphere.

Instruments. All reactions were strictly performed under nitrogen conditions. Proton nuclear magnetic resonance (^{1}H NMR) spectra were measured using Bruker DRX 300 (300 MHz) NMR spectrometer And, the carbon nuclear magnetic resonance (^{13}C NMR) spectra were measured on a Bruker DRX 500 (500 MHz) NMR spectrometer. High- resolution mass spectrometry (HR-MS) in chemical analysis was performed by the electron impact (EI) and fast atom bombardment (FAB) methods using a JMS-700 MStation (Jeol). Thermogravimetric analysis (TGA) was performed using a TGA 2050 (TA Instruments) thermogravimetric analyzer under a nitrogen atmosphere for the thermal stability of target materials. The samples were heated at 10 °C/min. Differential scanning calorimetry (DSC) was performed using a TA Instruments 2100 DSC operated under a nitrogen flow at a heating rate of 10 °C/min from 0 °C – 300 °C. The electrochemical properties of target materials were measured by cyclic voltammetry (CV) (ZIVE SP1, Wonatech) @ RT in a 0.1 M solution of Tetrabutylammonium perchlorate in CH_2Cl_2 at a scanning rate of 20 mV/s. We used a Pt wire as the counter electrode and an Ag/Ag+ electrode as the reference electrode, respectively. The redox potentials for target materials were reported with the reference to the ferrocene/ferrocenium (Fc/Fc^{+}) redox couple. The final products were purified by vacuum sublimation before the measurements and device fabrication. High-performance liquid chromatography (HPLC) analysis on Agilent Technologies (1260 infinity) equipped with a reversed-phase C18 HPLC column (InertSustain C18, GL Sciences Inc., 4.6 mm x 250 mm).

Device fabrication and measurements. We used 25 mm × 25 mm glass substrates with 70 nm half-patterned Indium-tin oxide (ITO) for the comprehensive OLED sets. Organic materials were deposited on ITO substrates by the thermal vacuum evaporation technique under ~ 10^{-7} Torr at the deposition rate of ~ 0.6 Å /s. MoO_x, LiF, and Al were formed with the deposition rate of 0.2 Å/s, 0.15 Å/s, and 0.85 Å/s, respectively. The current density–voltage (*J*–*V*) and luminance–voltage (*L*–*V*) data of tested OLEDs were measured by a programmable Keithley source meter (Keithley 2400), a calibrated photodiode (FDS100-cal, Thorlab), and a fiber optic spectrometer (Blue-Wave_UVNb, StellarNet). The active area for the tested OLED samples was 7.0 - 8.0 mm^2. To get well angle-resolved EL intensity characteristics for the tested OLED devices, we utilized the motorized goniometer (PRM1/MZ8, Thorlabs) equipped with the calibrated photodiode and the fiber optic spectrometer in a nitrogen-filled glove box. With this, we corrected the values of external quantum efficiency (EQE) and the power efficiency (PE) of our tested OLED devices from the full angular properties without Lambertian simplification.

Optical Modeling and analysis. We performed the wave-optic simulation based on coherent dipole radiation theory for OLED multi-layered architecture. It is known as CPS model, describing the system where the emitting dipole is placed in an organic layer near interfaces, which can consider Purcell effect (*F*), dipole orientation, and a series of coupled modes [e.g., Sub-confined, wave-guided, and surface plasmon polaritons (SPP)]. With this classical dipole oscillation model implemented as homemade MATLAB codes, we factored in the optically optimized deep-blue OLED architecture and confirmed that the simulated angle- and spectral-resolved data are

well correspondence to the experimental. Herein, we assume the emitting dipole with factored molecular anisotropy (Θ) locates at the center of the EML for all cases (i.e., fixed dipole model).

Angle dependent p-polarized PL profile. To investigate the transition dipole orientation (TDO) in EML, we used polarization- and angle-dependent PL spectroscopy (Phelos, FLUXiM). A series of thin films (target thickness: 50 nm) with binary (e.g., host-guest type) and ternary composition (e.g., hyperfluorescent type), deposited on a quartz substrate, was placed onto the goniometer equipped with a macroscopic half-cylinder prism. Herein, we used the certified refractive index liquid (1.5160 ± 0.0002) purchased from Cargille Labs that could be placed in between the quartz substrate and half-cylinder. Using a 365 nm UV-LED, the spot point on the target thin film is excited, thus, we obtained the polarized response from the emissive species by the emission angle (0-80°, in steps of 2.5^{o}). We then determined TDO by fitting the measured *p*-polarized PL profile that is normalized intensity to 0° with an optical system modeled by CPS model (refer to *optical modeling and analysis*).

II. Synthesis and Characterization

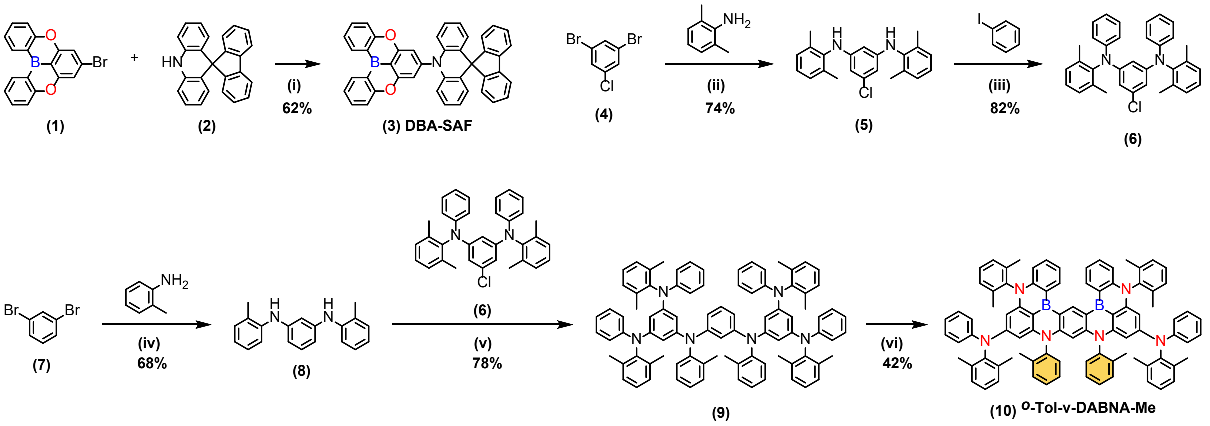

Figure S1. Synthetic procedures: i) NaO*t*Bu, 1.0 M P(*t*-Bu)$_3$, Pd$_2$(dba)$_3$, Toluene, 120 °C, 12 h; ii) NaO*t*Bu, BINAP, Pd$_2$(dba)$_3$, Toluene, 120 °C, 12 h; iii) NaO*t*Bu, 1.0 M P(*t*-Bu)$_3$, Pd$_2$(dba)$_3$, Toluene, 120 °C, 12 h; iv) NaO*t*Bu, BINAP, Pd$_2$(dba)$_3$, Toluene, 120 °C, 12 h; v) NaO*t*Bu, 1.0 M P(*t*-Bu)$_3$, Pd$_2$(dba)$_3$, Toluene, 120 °C, 12 h; vi) 1. BBr$_3$, *o*-DCB, 180 °C, 20 h; 2. *i*-Pr$_2$NEt, O °C.

Synthesis of 10-(5,9-dioxa-13b-boranaphtho[3,2,1-*de*]anthracen-7-yl)-10*H*-spiro[acridine-9,9'-fluorene] (3) (DBA-SAF): Compound 1 (2.0 g, 5.7 mmol), compound 2 (2.08 g, 6.3 mmol), sodium *tert*-butoxide (0.83 g, 8.6 mmol), tri-*tert*-butylphosphine (1.0 M solution in toluene, 0.57 mL, 0.57 mmol) and tris(dibenzylideneacetone)palladium(0) (0.21 g, 0.228 mmol) were dissolved in anhydrous toluene (30.0 mL) under a nitrogen atmosphere. After stirring at 120 °C for 12 h, the reaction mixture was filtered through a pad of Florisil® using toluene as an eluent. After the solvent was evaporated, the crude product was purified by column chromatography (eluent = *n*-hexane/toluene, 2:1). After purification, the product was obtained as a white solid (yield: 2.1 g, 62%). ^{1}H NMR (500 MHz, CD_2Cl_2) δ 8.85 (dd, *J* = 7.6, 1.7 Hz, 2H), 7.91 (d, *J* = 7.6 Hz, 2H), 7.85 (ddd, *J* = 8.5, 7.0, 1.7 Hz, 2H), 7.68 (d, *J* = 8.1 Hz, 2H), 7.53 (dd, *J* = 11.8, 7.4 Hz, 4H), 7.47 (d, *J* = 2.6 Hz, 4H), 7.41 – 7.34 (m, 2H), 6.98 (ddd, *J* = 8.5, 7.0, 1.6 Hz, 2H), 6.68 – 6.61 (m, 2H), 6.58 (d, *J* = 8.4 Hz, 2H), 6.46 (dd, *J* = 7.8, 1.6 Hz, 2H), 5.36 (d, *J* = 1.7 Hz, 2H). ^{13}C NMR (126 MHz, CD_2Cl_2) δ 160.68, 156.45, 140.95, 139.29, 134.74, 134.17, 128.41, 127.76, 127.51, 127.34, 125.58, 124.89, 123.32, 120.78, 120.08, 118.51, 114.95, 111.18. HRMS (FAB+, m/z): calcd for $C_{43}H_{26}BNO_2$ 599.2057, found 599.2051.

Synthesis of 5-chloro-*N*^{1},*N*^{3}-bis(2,6-dimethylphenyl)benzene-1,3-diamine (5): Compound 4 (10.0 g, 36.9 mmol), 2,6-dimethylaniline (9.85 g, 81.3 mmol), sodium *tert*-butoxide (10.6 g, 110.8 mmol), BINAP (1.38 g, 2.21 mmol) and tris(dibenzylideneacetone)palladium(0) (1.01 g, 1.10 mmol) were dissolved in anhydrous toluene (200.0 mL) under a nitrogen atmosphere. After stirring at 120 °C for 12 h, the reaction mixture was filtered through a pad of Florisil® using toluene as an eluent. After the solvent was evaporated, the crude product was wash with hexane. After purification, the product was obtained as a white solid (yield: 9.6 g, 74%). ^{1}H NMR (500 MHz, CD_2Cl_2) δ 7.32 – 6.95 (m, 6H), 5.87 (d, *J* = 2.0 Hz, 2H), 5.63 (t, *J* = 2.0 Hz, 1H), 5.21 (s, 2H), 2.24 (s, 12H). ^{13}C NMR (126 MHz, CD_2Cl_2) δ 148.64, 137.68, 136.29, 135.57, 128.44, 126.13, 103.46, 96.47, 18.00. HRMS (EI+, m/z): calcd for $C_{22}H_{23}ClN_2$ 350.1550, found 350.1550.

Synthesis of 5-chloro-*N*^{1},*N*^{3}-bis(2,6-dimethylphenyl)-*N*^{1},*N*^{3}-diphenylbenzene-1,3-diamine (6): Compound 5 (9.5 g, 27.1 mmol), iodobenzene (16.6 g, 81.3 mmol), sodium *tert*-butoxide (7.8 g, 81.3 mmol), tri-*tert*-butylphosphine (1.0 M solution in toluene, 1.08 mL, 1.08 mmol) and tris(dibenzylideneacetone)palladium(0) (0.49 g, 0.542 mmol) were dissolved in anhydrous toluene (80.0 mL) under a nitrogen atmosphere. After stirring at 120 °C for 12 h, the reaction mixture was filtered through a pad of Florisil® using toluene as an eluent. After the solvent was evaporated, the crude product was wash with hexane and methanol. After purification, the product was obtained as a white solid (yield: 11.1 g, 82%). ^{1}H NMR (500 MHz, CD_2Cl_2) δ 7.20 (ddd, *J* = 10.5, 8.6, 6.7 Hz, 6H), 7.14 (d, *J* = 8.2 Hz, 4H), 6.99 – 6.89 (m, 6H), 6.67 (t, *J* = 2.1 Hz, 1H), 6.31 (d, *J* = 2.1 Hz, 2H), 2.05 (s, 12H). ^{13}C NMR (126 MHz, CD_2Cl_2) δ 147.75, 145.13, 142.04, 137.76, 135.36, 129.20, 128.98, 127.39, 121.45, 120.24, 111.30, 108.27, 18.22. HRMS (EI+, m/z): calcd for $C_{34}H_{31}ClN_2$ 502.2176, found 502.2175.

Synthesis of *N*^{1},*N*^{3}-di-*o*-tolylbenzene-1,3-diamine (8)

Compound 7 (3.0 g, 12.7 mmol), *o*-toluidine (4.08 g, 38.1 mmol), sodium *tert*-butoxide (3.6 g, 38.1 mmol), BINAP (0.47 g, 0.762 mmol) and tris(dibenzylideneacetone)palladium(0) (0.35 g, 0.381 mmol) were dissolved in

anhydrous toluene (40.0 mL) under a nitrogen atmosphere. After stirring at 120 °C for 12 h, the reaction mixture was filtered through a pad of Florisil® using toluene as an eluent. After the solvent was evaporated, the crude product was wash with hexane. After purification, the product was obtained as a white solid (yield: 2.48 g, 68%). ^{1}H NMR (500 MHz, CD_2Cl_2) δ 7.32 (dd, *J* = 8.0, 1.3 Hz, 2H), 7.26 (dd, *J* = 7.5, 1.6 Hz, 2H), 7.23 – 7.13 (m, 3H), 6.99 (td, *J* = 7.4, 1.3 Hz, 2H), 6.61 – 6.52 (m, 3H), 5.46 (s, 2H), 2.31 (s, 6H). ^{13}C NMR (126 MHz, CD_2Cl_2) δ 145.37, 141.16, 130.88, 130.01, 128.78, 126.64, 122.08, 119.37, 109.33, 105.43, 17.67. HRMS (EI+, m/z): calcd for $C_{20}H_{20}N_2$ 288.1626, found 288.1629.

Synthesis of *N*^{1},*N*$^{1'}$-(1,3-phenylene)bis(*N*^{3},*N*^{5}-bis(2,6-dimethylphenyl)-*N*^{3},*N*^{5}-diphenyl-*N*^{1}-(*o*-tolyl)benzene-1,3,5-triamine) (9): Compound 6 (5.75 g, 11.4 mmol), compound 8 (1.5 g, 5.2 mmol), sodium *tert*-butoxide (4.0 g, 41.6 mmol), tri-*tert*-butylphosphine (1.0 M solution in toluene, 0.52 mL, 0.52 mmol) and tris(dibenzylideneacetone)palladium(0) (0.19 g, 0.208 mmol) were dissolved in anhydrous toluene (40.0 mL) under a nitrogen atmosphere. After stirring at 120 °C for 12 h, the reaction mixture was filtered through a pad of Florisil® using toluene as an eluent. After the solvent was evaporated, the crude product was purified by column chromatography (eluent = *n*-hexane/dichloromethane, 4:1). After purification, the product was obtained as a white solid (yield: 4.95 g, 78%). ^{1}H NMR (500 MHz, CD_2Cl_2) δ 7.11 – 7.04 (m, 18H), 7.00 (d, J = 7.5 Hz, 8H), 6.86 – 6.84 (m, 2H), 6.84 – 6.74 (m, 14H), 6.23 (dd, J = 8.1, 2.2 Hz, 2H), 6.13 (t, J = 2.0 Hz, 2H), 6.08 (d, J = 2.0 Hz, 4H), 1.93 (s, 24H), 1.90 (s, 6H). ^{13}C NMR (126 MHz, CD_2Cl_2) δ 148.08, 147.60, 146.68, 145.68, 145.29, 142.38, 137.68, 136.00, 131.27, 129.06, 128.88, 128.80, 126.88, 126.76, 125.54, 120.24, 119.11, 114.39, 112.81, 106.45, 105.34, 18.14, 18.07. HRMS (FAB+, m/z): calcd for $C_{88}H_{80}N_6$ 1220.6444, found 1220.6444.

Synthesis of *N*^{7},*N*^{7},*N*^{7},*N*^{13},5,9,11,15-tetrakis(2,6-dimethylphenyl)-9,11-bis(*o*-tolyl)phenyl)-5,9,11,15-tetrahydro-5,9,11,15-tetraaza-19b,20b-diboradinaphtho[3,2,1-*de*:1',2',3'-*jk*]pentacene-7,13-diamine (10) (*o*-Tol-*ν*-DABNA-Me): Compound 9 (4.5 g, 3.68 mmol) was dissolved in o-dichlorobenzene (50.0 mL) and bo ron tribromide (2.09 mL, 22.1 mmol) was added under a nitrogen atmosphere. Then stirring at 200 °C for 20 h, the reaction mixture was allowed to cool to room temperature. After addition of N,N-diisopro pylethylamine (9.6 mL, 55.2 mmol) was stirred 30 min at 0 °C, and then the reaction mixture was filt ered through a pad of Florisil® using toluene as an eluent. After the solvent was evaporated, the crud e product was purified by column chromatography (eluent = n-hexane/toluene, 7:3). After purification, t he product was obtained as a yellow solid (yield: 1.91 g, 42%). Final product was purified by sublima tion (385 °C, 1.0 × 10^{-5} Pa) to obtain the title compound (yield: 1.49 g, 33%). ^{1}H NMR (500 MHz, $(CDCl_2)_2$) δ 10.69 (d, J = 4.4 Hz, 1H), 9.44 (d, J = 8.0 Hz, 2H), 7.53 (t, J = 7.5 Hz, 2H), 7.46 (q, J = 6.5 Hz, 2H), 7.31 – 7.00 (m, 22H), 6.98 (d, J = 6.7 Hz, 4H), 6.93 (s, 3H), 6.83 (dt, J = 25.7, 7.0 Hz, 4H), 5.77 – 5.73 (m, 2H), 5.56 (s, 2H), 2.00 – 1.85 (m, 30H). ^{13}C NMR (126 MHz, $(CDCl_2)_2$) δ 149.98, 149.61, 147.72, 146.36, 142.70, 139.65, 137.83, 137.83, 137.53, 137.52, 129.29, 128.81, 128.52, 128.24, 127.05, 121.06, 119.97, 115.43, 96.52, 95.34, 67.95, 25.78, 17.97, 17.39, 16.89. HRMS (FAB+, m/z): calcd for $C_{88}H_{74}B_2N_6$ 1236.6161, found 1236.6158.

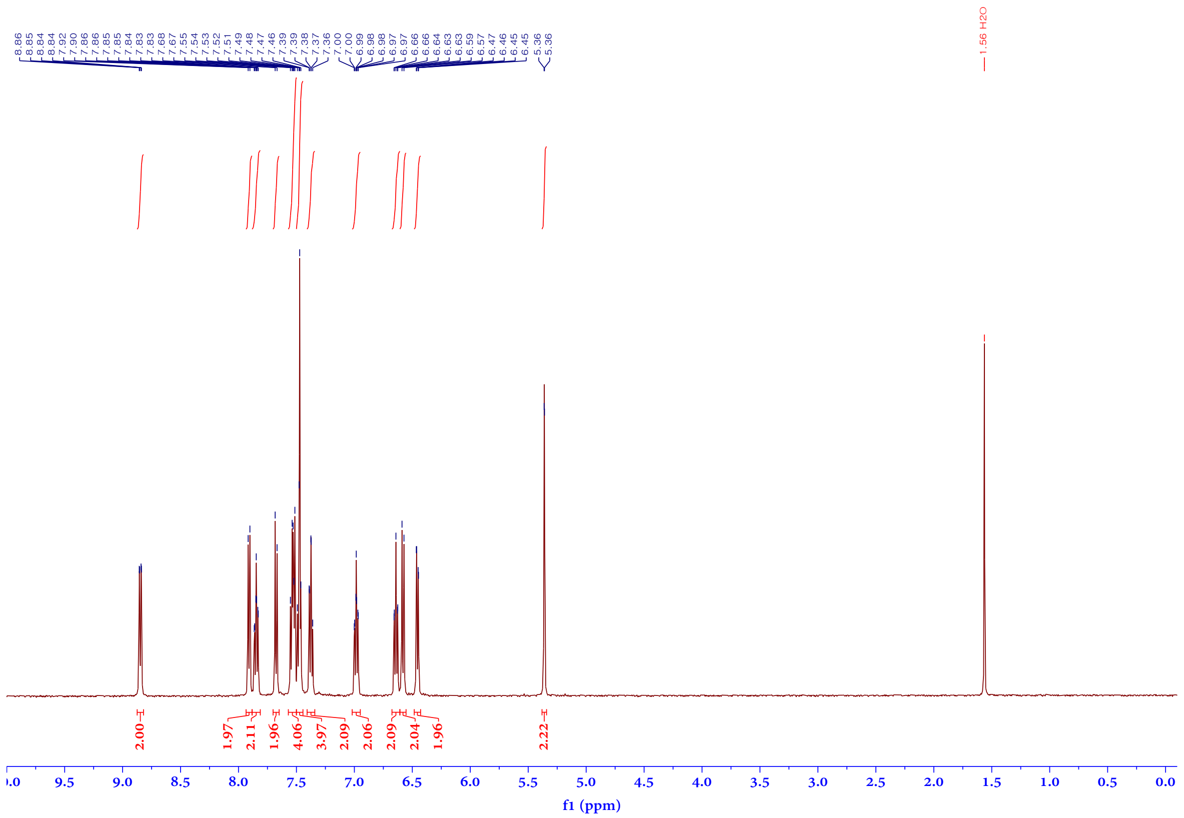

Figure S2. ^{1}H-NMR spectrum of compound 3 (**DBA-SAF**) in CD_2Cl_2 at 25 ºC.

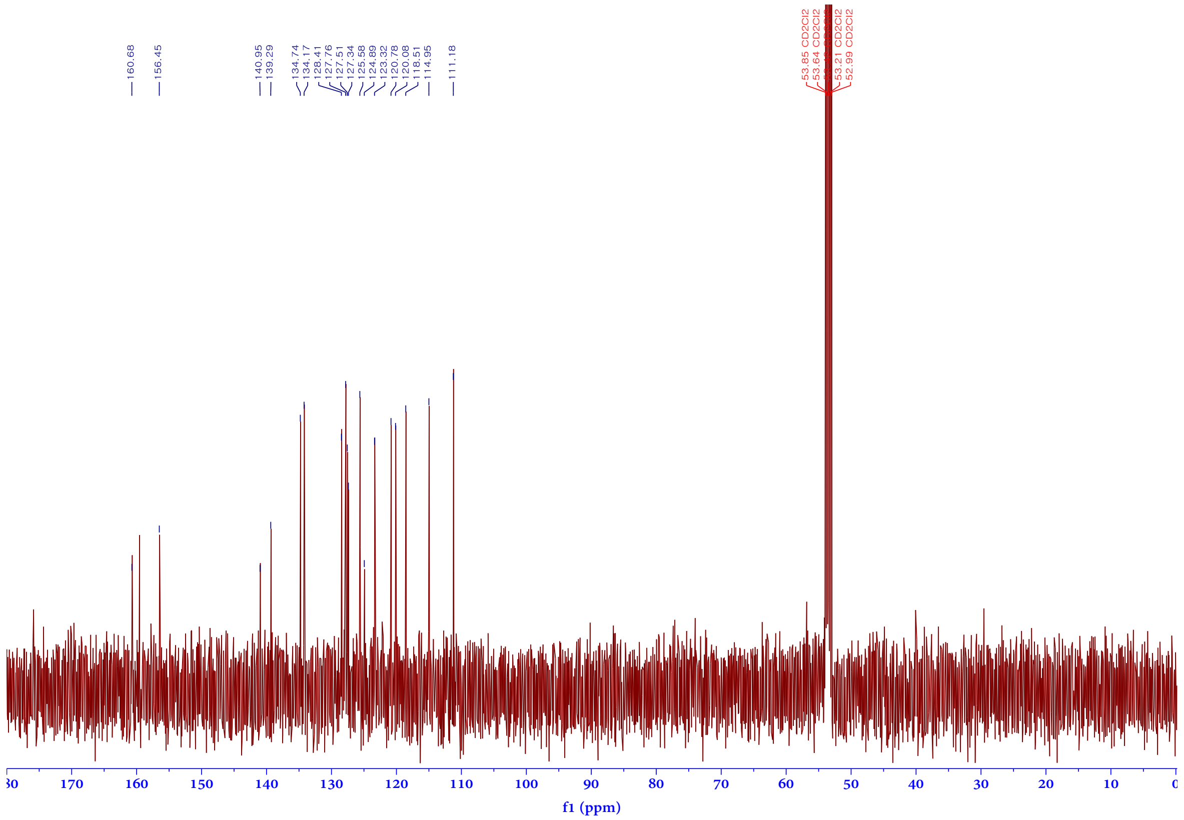

Figure S3. ^{13}C-NMR spectrum of compound 3 (**DBA-SAF**) in CD_2Cl_2 at 25 ºC.

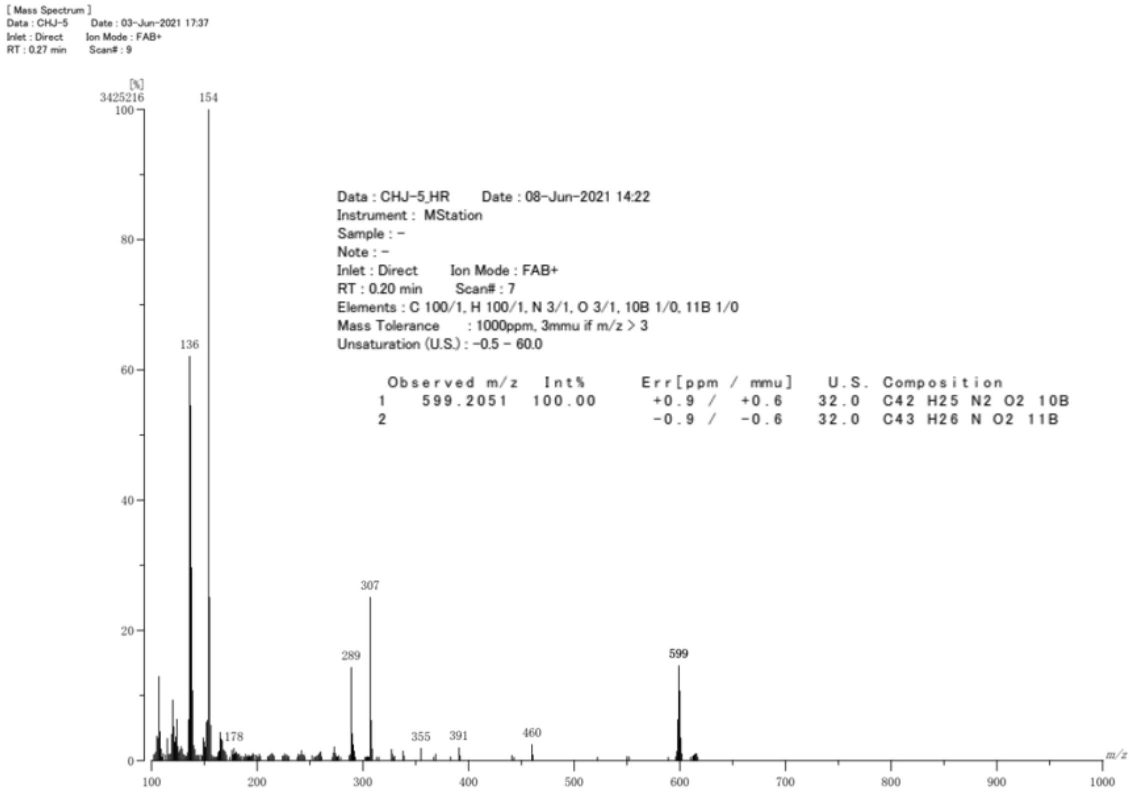

Figure S4. FAB+ Mass spectrum of compound 3 (**DBA-SAF**)

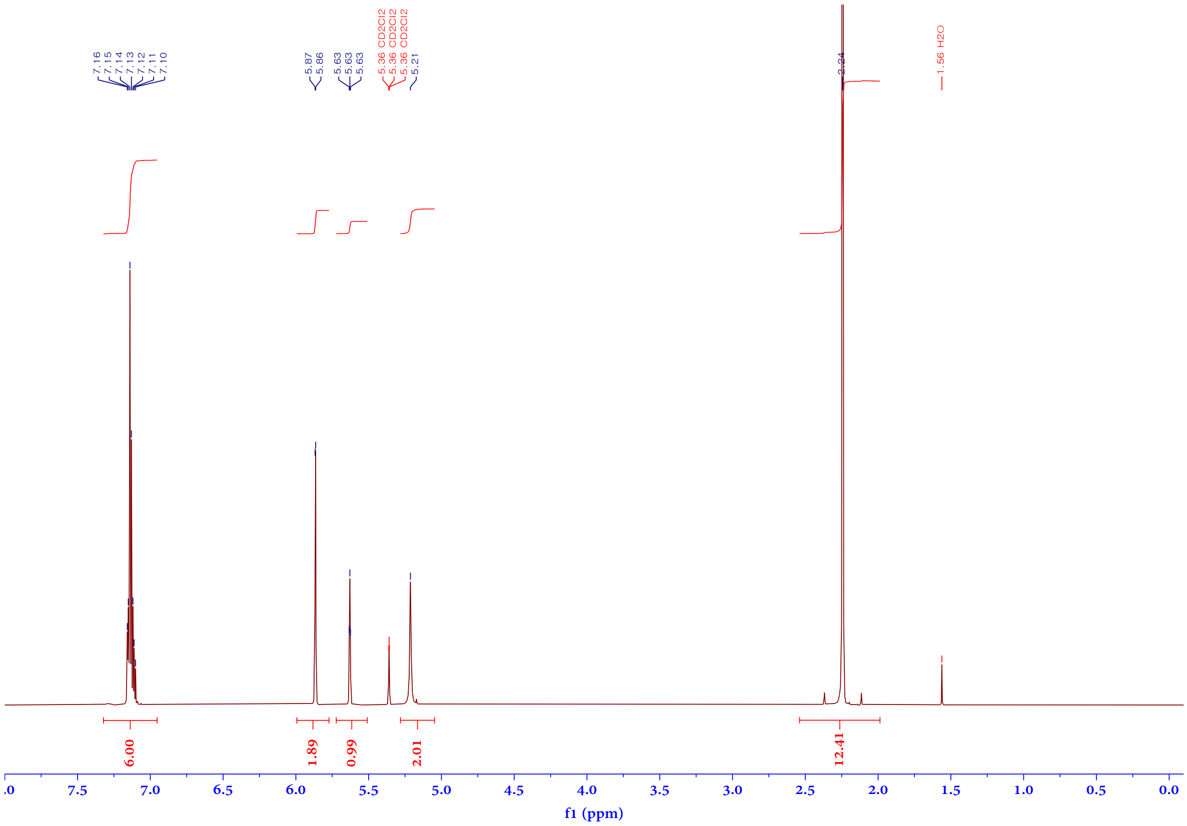

Figure S5. ^{1}H-NMR spectrum of compound 5 in CD_2Cl_2 at 25 ºC.

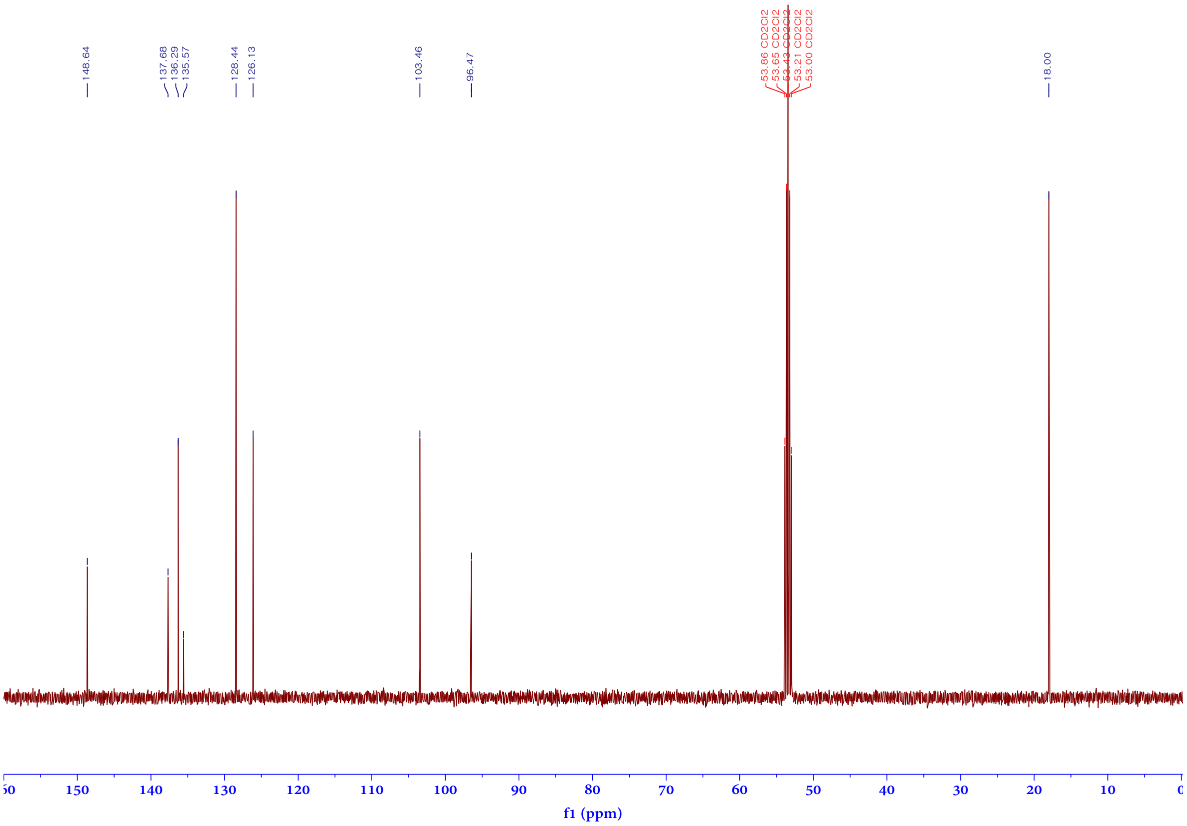

Figure S6. ^{13}C-NMR spectrum of compound 5 in CD_2Cl_2 at 25 ºC.

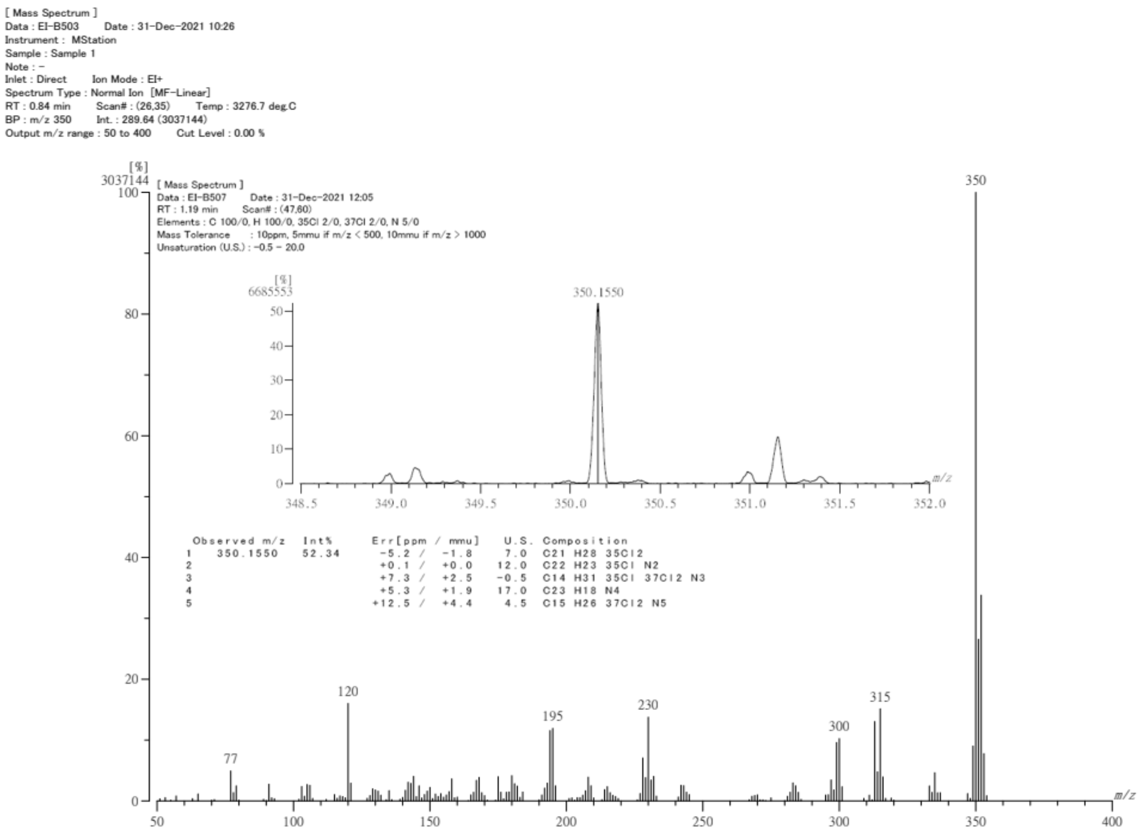

Figure S7. EI+ Mass spectrum of compound 5

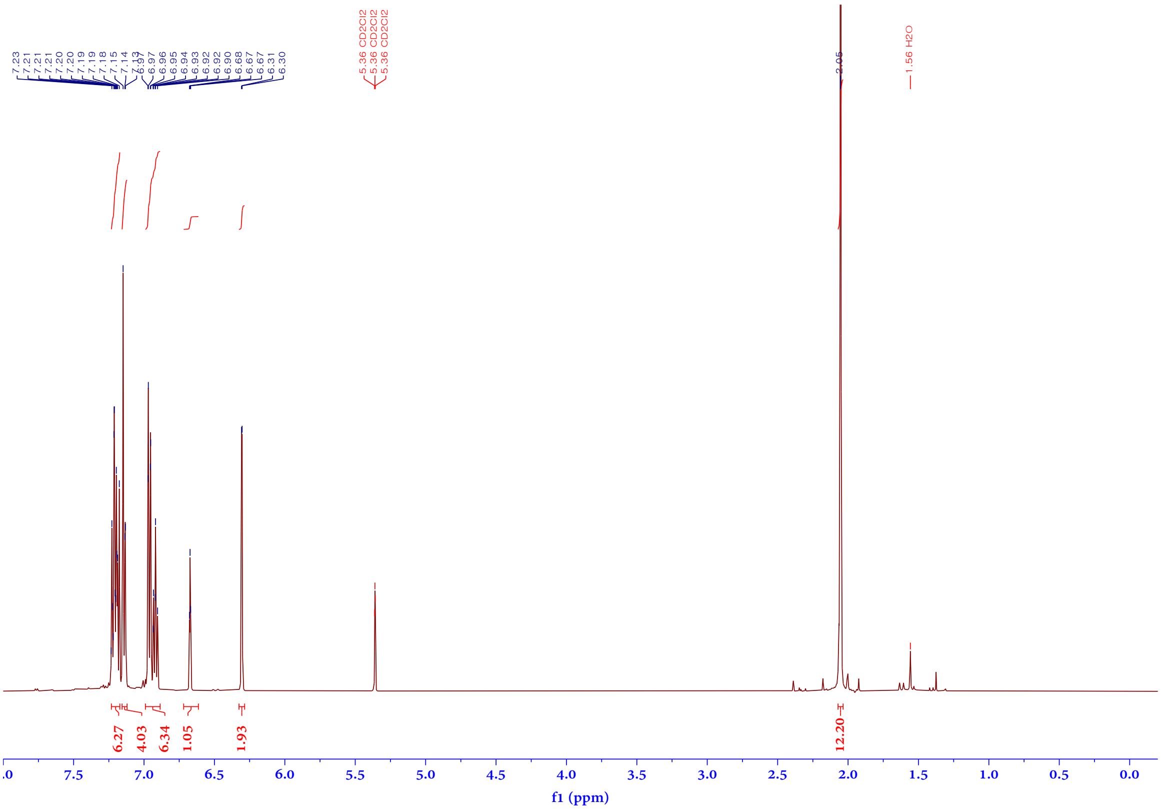

Figure S8. ^{1}H-NMR spectrum of compound 6 in CD_2Cl_2 at 25 ºC.

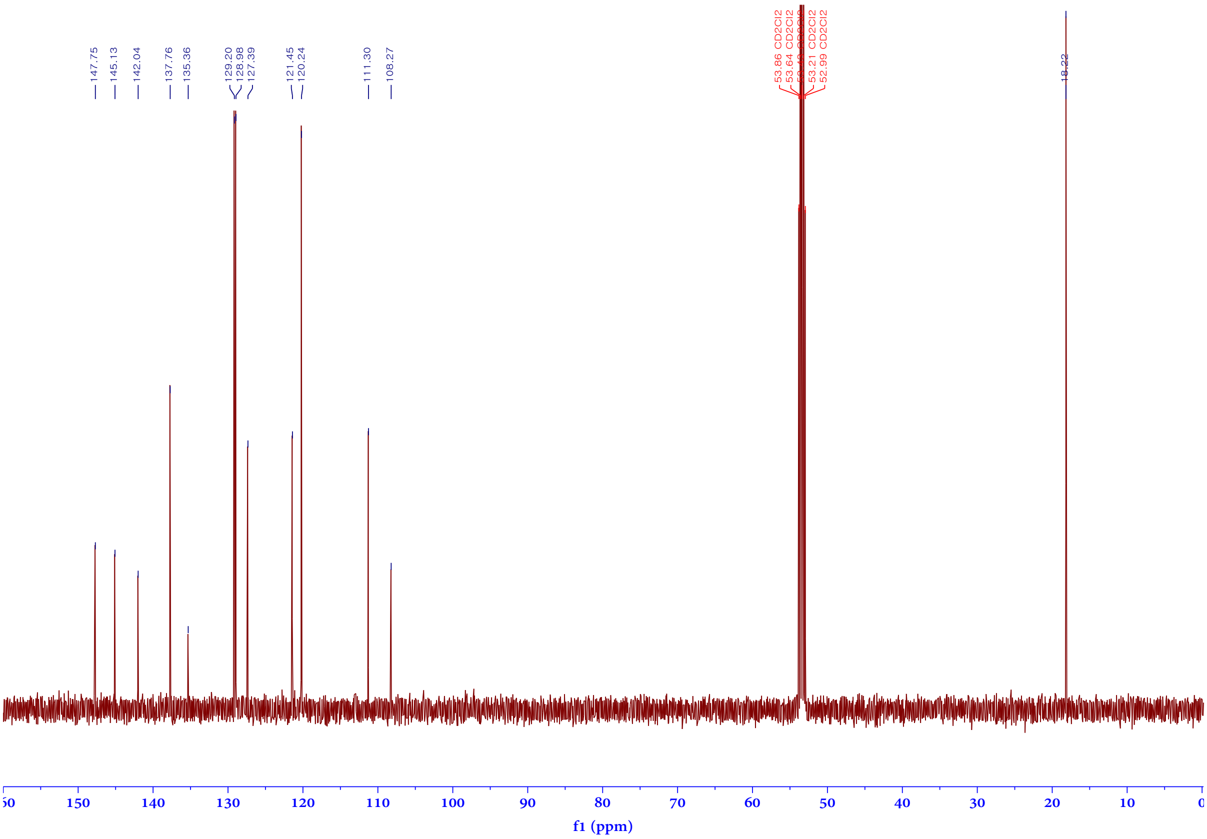

Figure S9. ^{13}C-NMR spectrum of compound 6 in CD_2Cl_2 at 25 ºC.

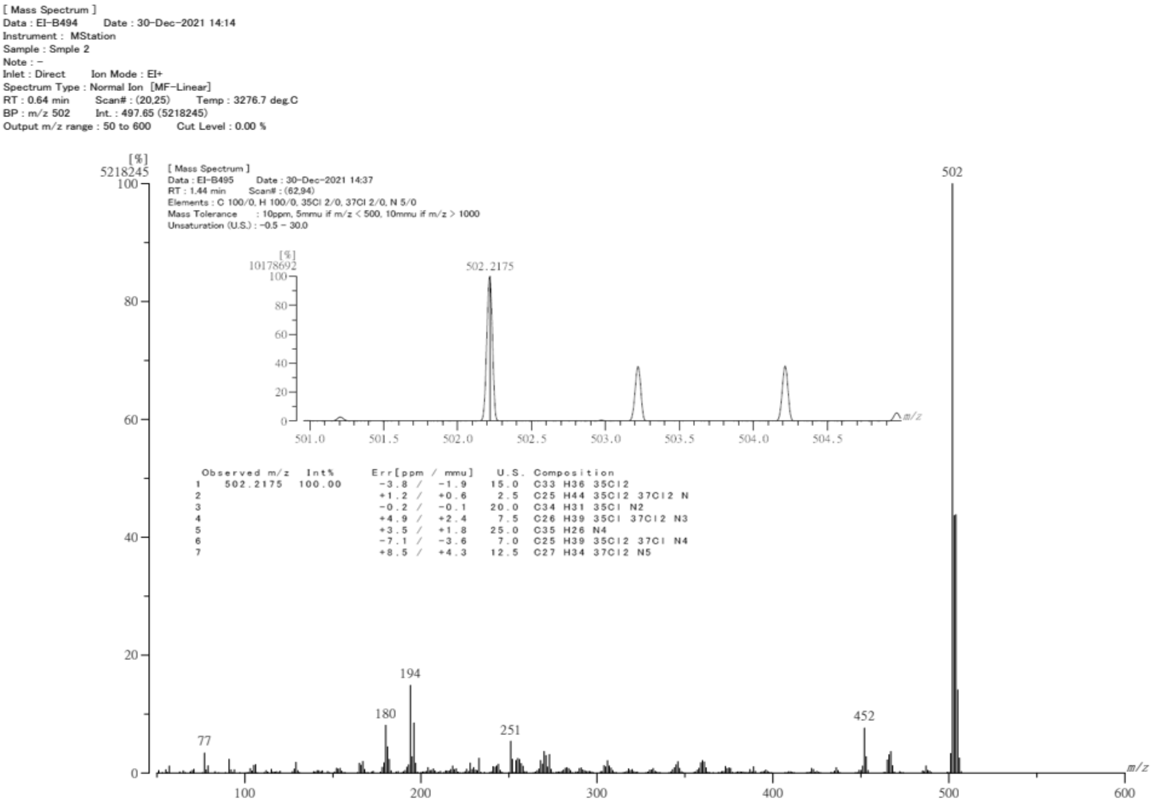

Figure S10. EI+ Mass spectrum of compound 6

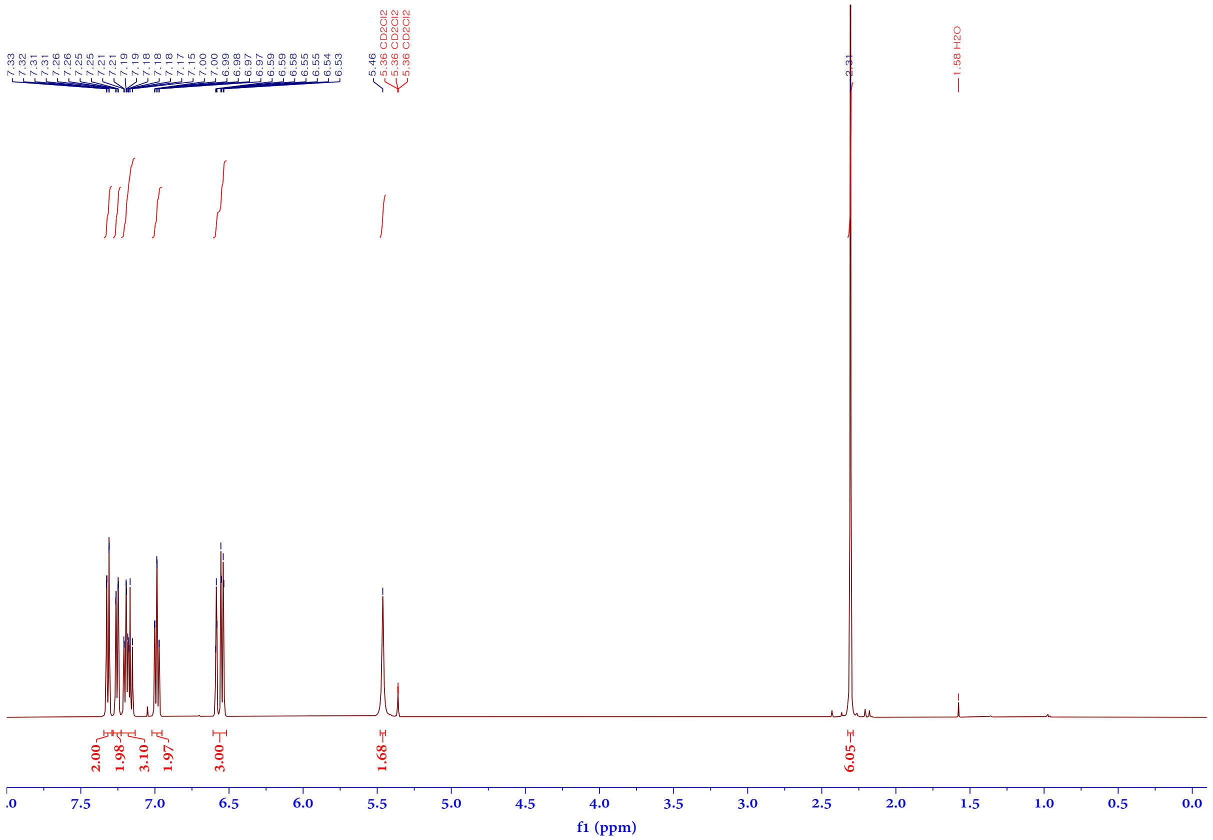

Figure S11. ^{1}H-NMR spectrum of compound 8 in CD_2Cl_2 at 25 ºC.

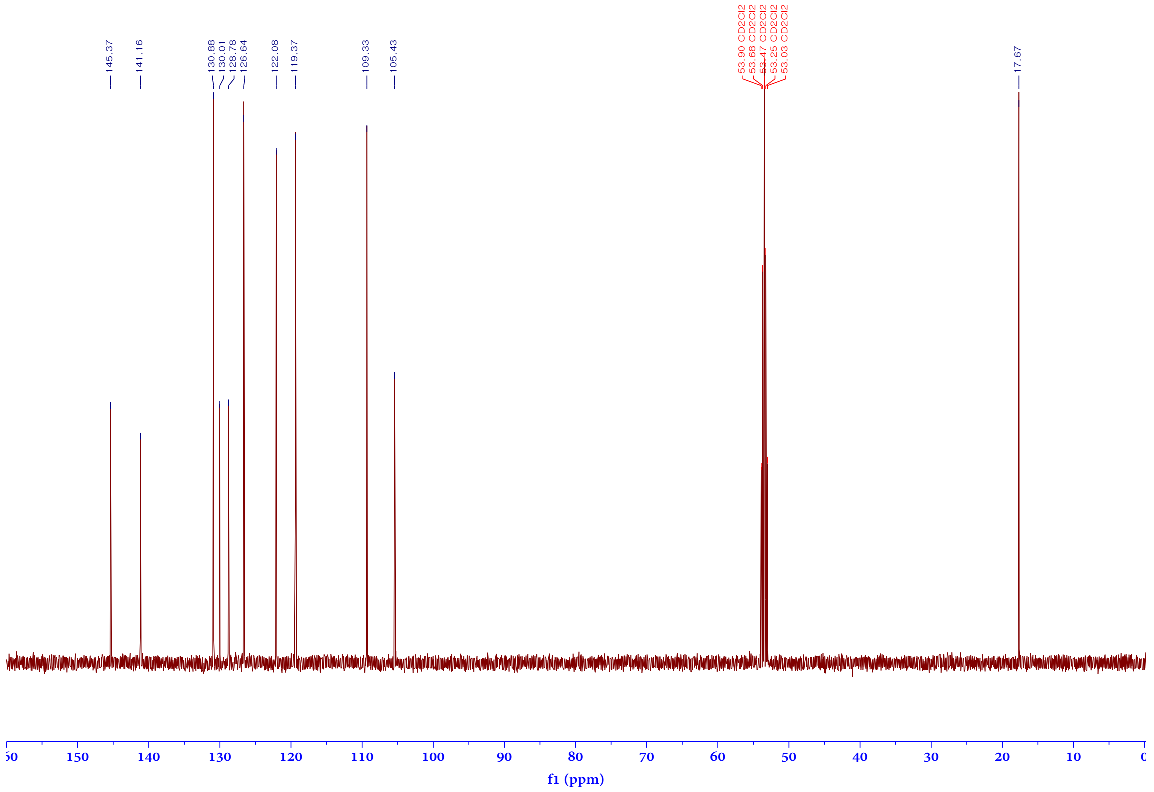

Figure S12. ^{13}C-NMR spectrum of compound 8 in CD_2Cl_2 at 25 ºC.

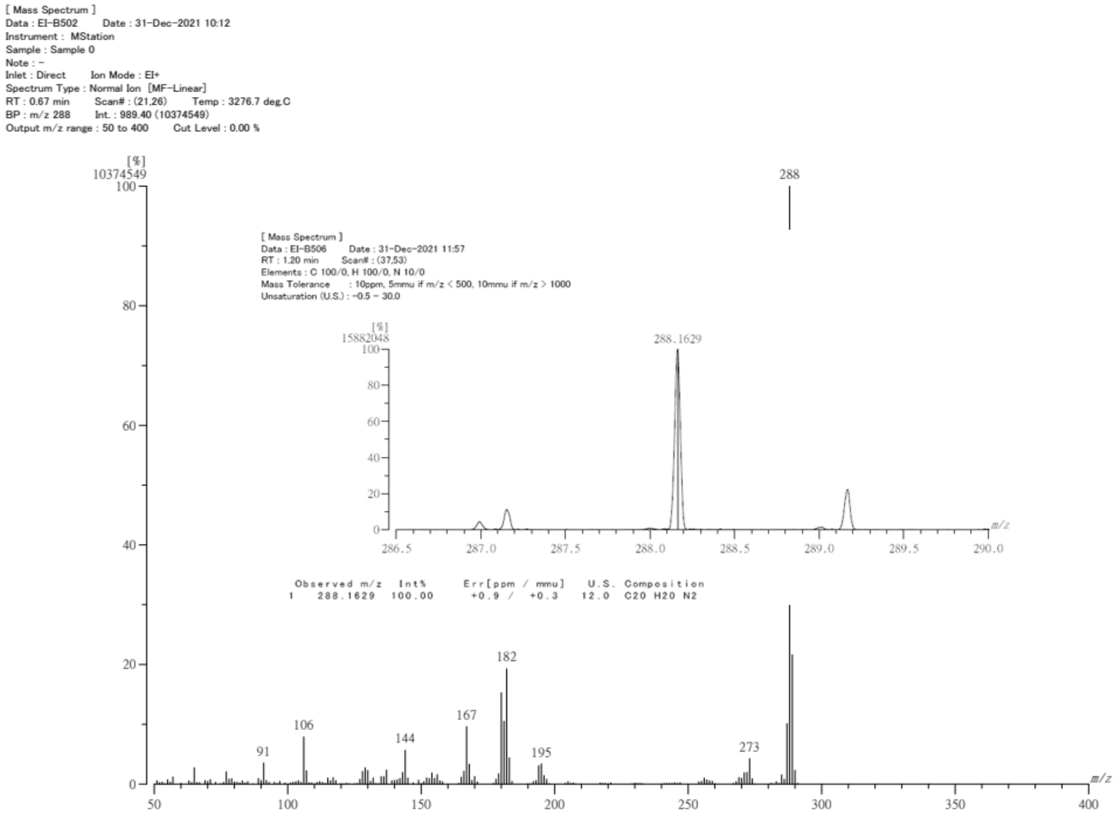

Figure S13. EI+ Mass spectrum of compound 8

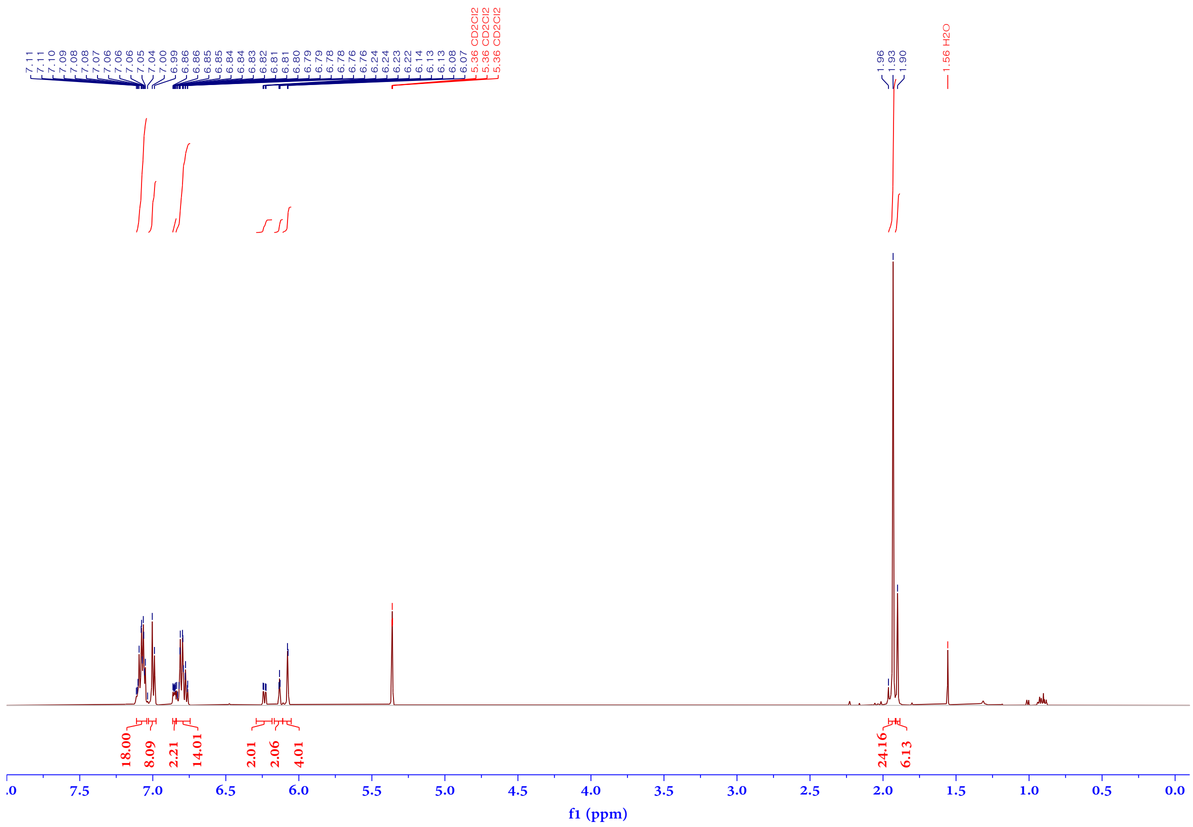

Figure S14. ^{1}H-NMR spectrum of compound 9 in CD_2Cl_2 at 25 ºC.

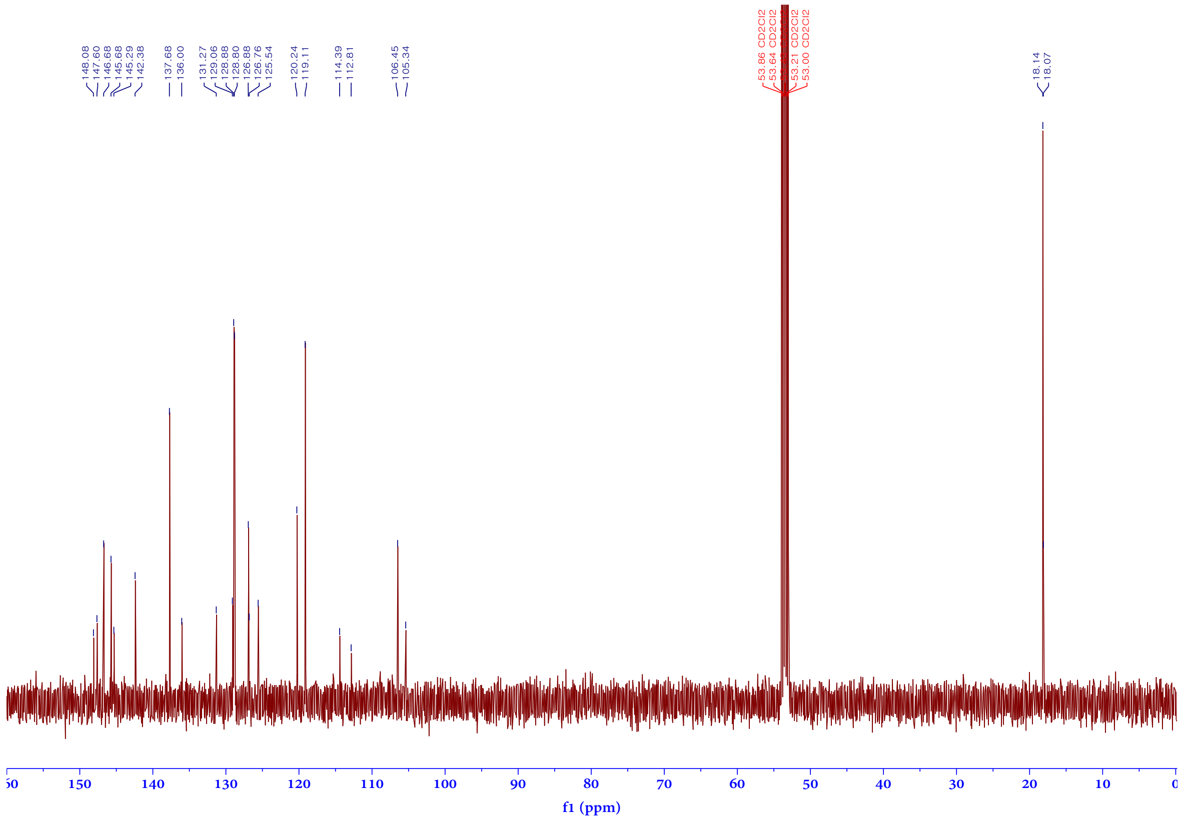

Figure S15. ^{13}C-NMR spectrum of compound 9 in CD_2Cl_2 at 25 ºC.

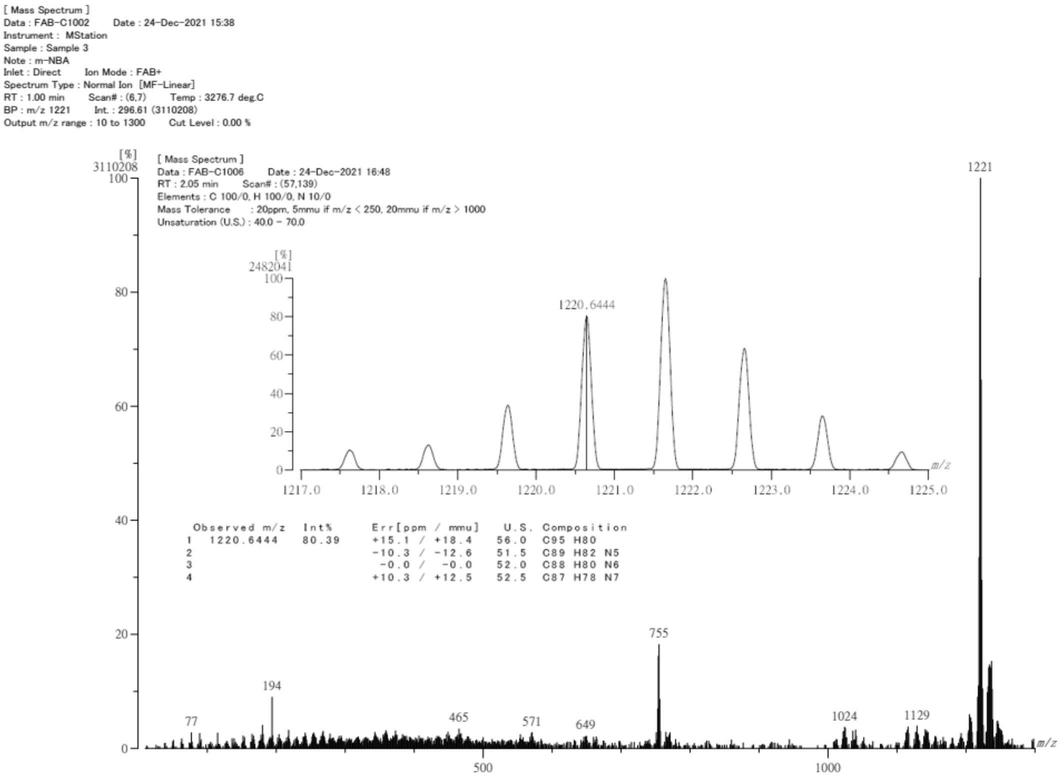

Figure S16. FAB+ Mass spectrum of compound 9

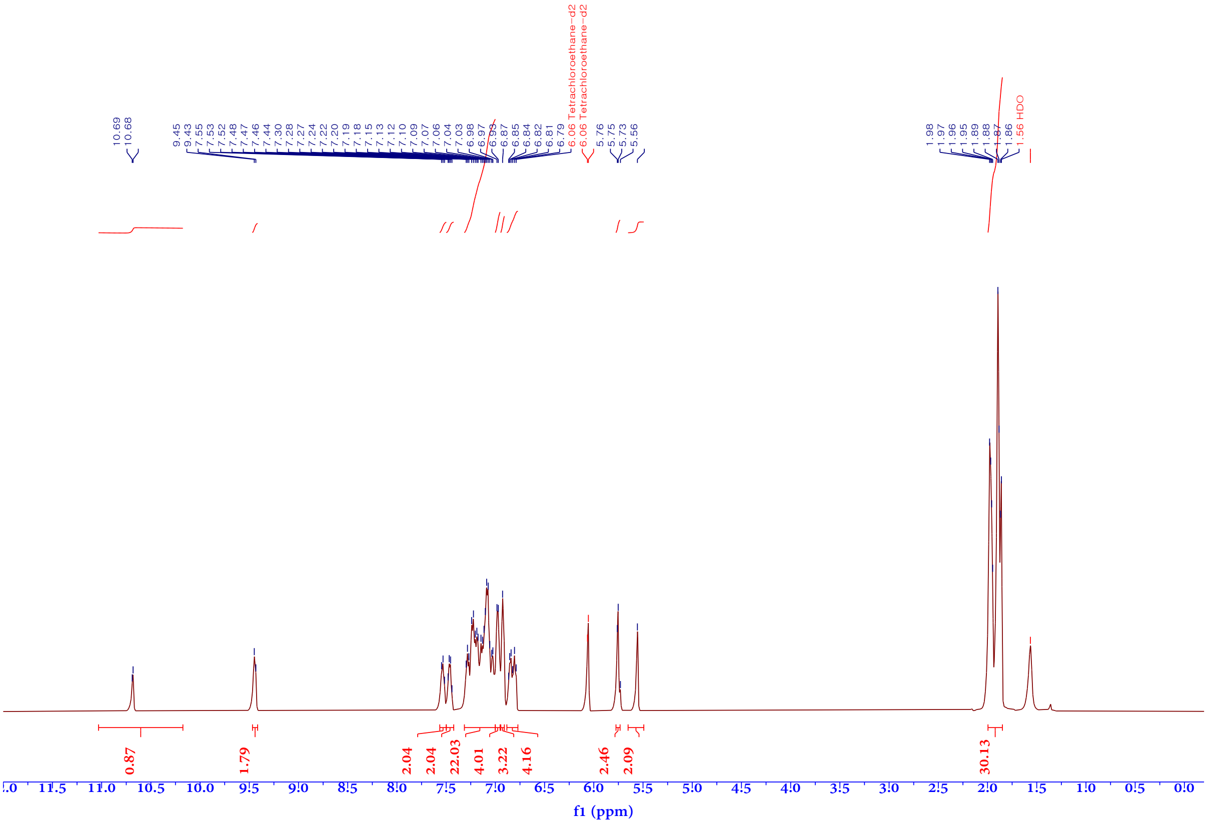

Figure S17. ^{1}H-NMR spectrum of compound 10 (***o*-Tol-*v*-DABNA-Me**) in CD_2Cl_2 at 80 ºC.

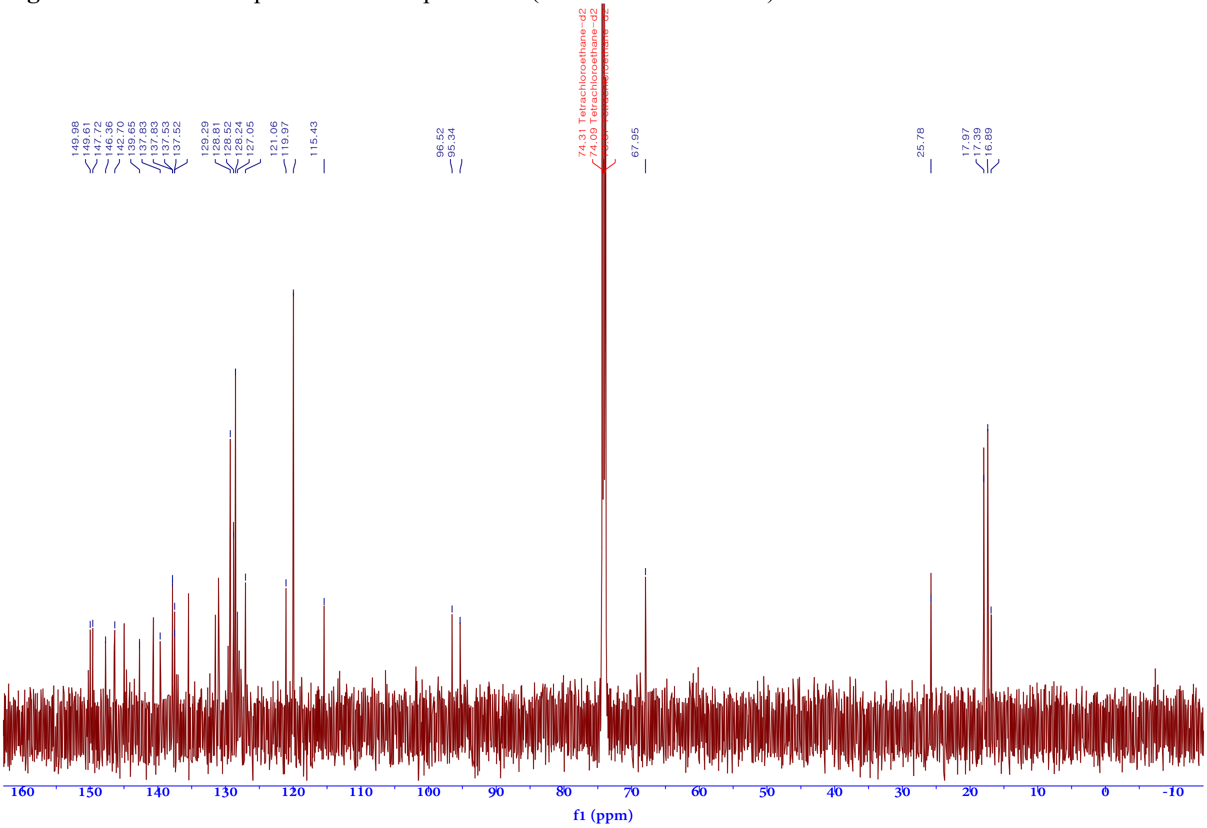

Figure S18. ^{13}C-NMR spectrum of compound 10 (***o*-Tol-*v*-DABNA-Me**) in CD_2Cl_2 at 80 ºC.

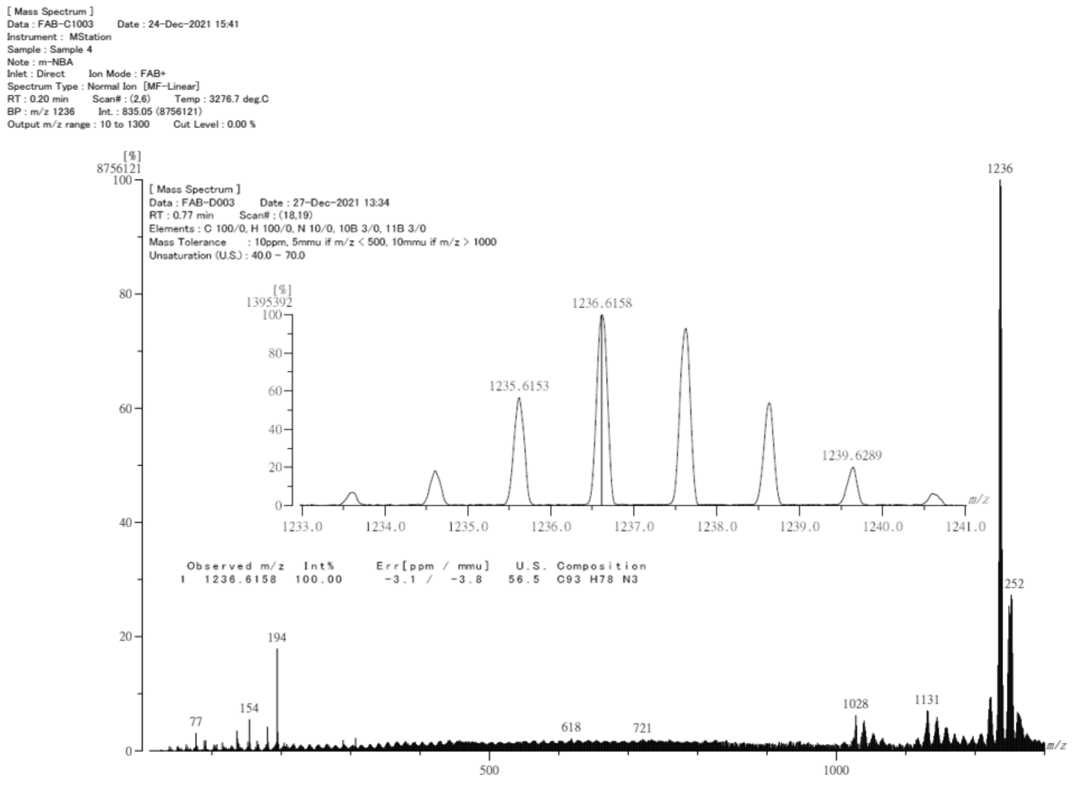

Figure S19. FAB+ Mass spectrum of compound 10 (***o*-Tol-*v*-DABNA-Me**).

Single crystal information. The crystal of ***o*-Tol-*v*-DABNA-Me** was grown by slow evaporation in tetrahydrofuran and acetonitrile solvents. A single-crystal suitable for X-ray measurement was coated with paratone oil and mounted on a glass fiber. The measurements of crystal were performed using a BRUKER (APEX2 ULTRA) diffractometer with an APEX II 4K CCD detector. The crystal was kept at 173.0 K during data collection. The structure was solved by direct methods (*29*) and refined by full-matrix least-squares fitting on F^2 using SHELXL-2014. (*30*) Full details of the crystal data is deposited as a cif with The Cambridge Crystallographic Data Centre (CCDC deposition number: 2132218). The data can be obtained free of charge via The Cambridge Crystallographic Data Centre "http://www.ccdc.cam.ac.uk/data request/cif".

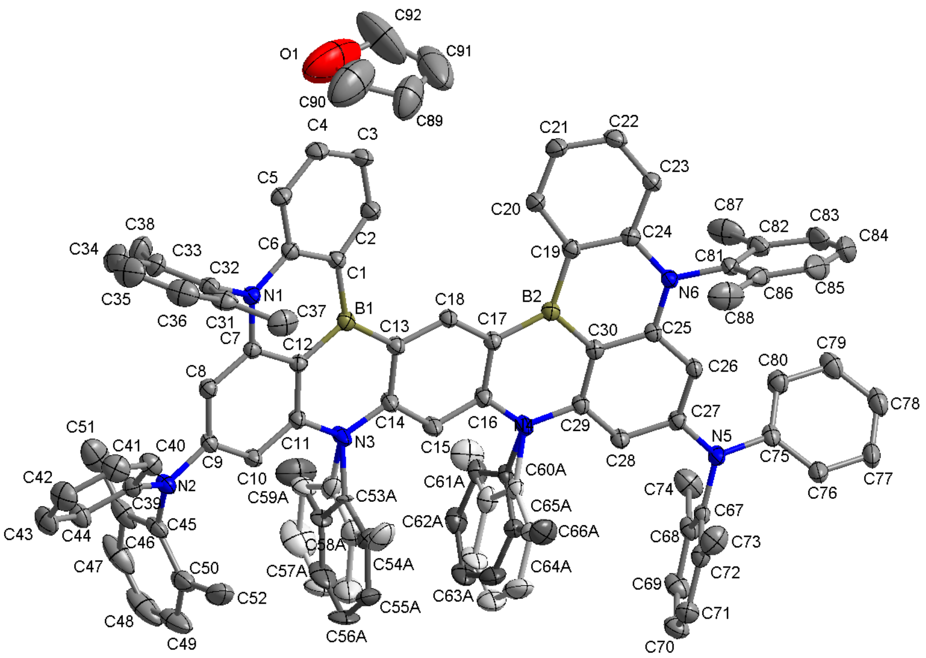

Figure S20. Crystal structure of ***o*-Tol-*v*-DABNA-Me.**

Table S1. Crystal data and structure refinement ***o*-Tol-*v*-DABNA-Me**.

Identification code	*o*-Tol-*v*-DABNA-Me_a	
Empirical formula	$C_{92}H_{82}B_2N_6O$	
Formula weight	1309.25	
Temperature	173(2) K	
Wavelength	0.71073 Å	
Crystal system	Monoclinic	
Space group	$P2_1/c$	
Unit cell dimensions	a = 21.2694(3) Å	α = 90°.
	b = 14.0774(2) Å	β = 103.2960(10)°.
	c = 25.1844(3) Å	γ = 90°.
Volume	7338.53(17) $Å^3$	
Z	4	
Density (calculated)	1.185 Mg/m^3	
Absorption coefficient	0.069 mm^{-1}	
F(000)	2776	
Crystal size	0.270 x 0.180 x 0.120 mm^3	
Theta range for data collection	1.662 to 26.000°.	
Index ranges	$-26 \leq h \leq 26$, $-17 \leq k \leq 17$, $-31 \leq l \leq 31$	
Reflections collected	185290	
Independent reflections	14442 [R_{int} = 0.0774]	
Completeness to theta = 25.242°	100.00%	
Absorption correction	Semi-empirical from equivalents	
Max. and min. transmission	0.7456 and 0.7141	
Refinement method	Full-matrix least-squares on F^2	
Data / restraints / parameters	14442 / 0 / 1036	
Goodness-of-fit on F^2	1.015	
Final R indices [I>2sigma(I)]	R1 = 0.0649, wR2 = 0.1595	
R indices (all data)	R1 = 0.1054, wR2 = 0.1890	
Extinction coefficient	n/a	
Largest diff. peak and hole	0.528 and -0.496 e.$Å^{-3}$	

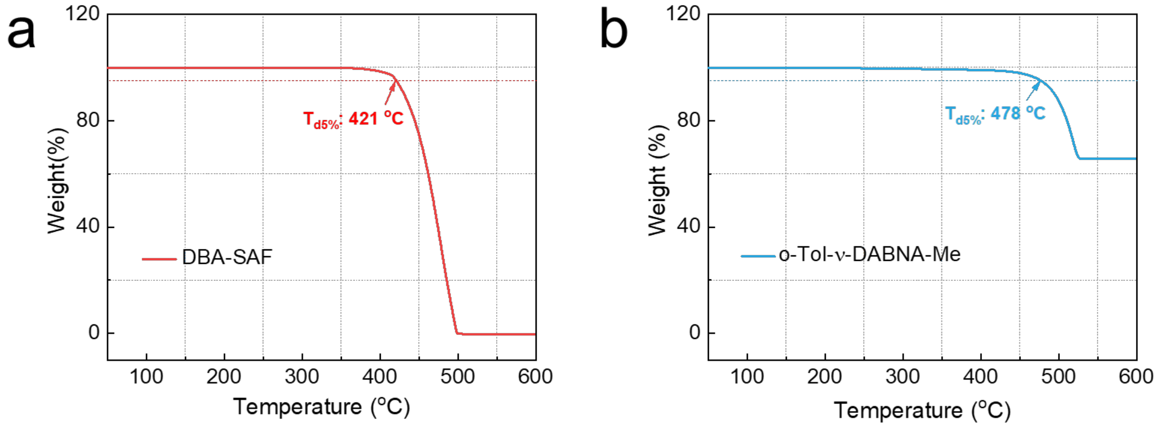

Figure S21. TGA measurements of (a) **DBA-SAF** and (b) ***o*-Tol-*ν*-DABNA-Me**, respectively.

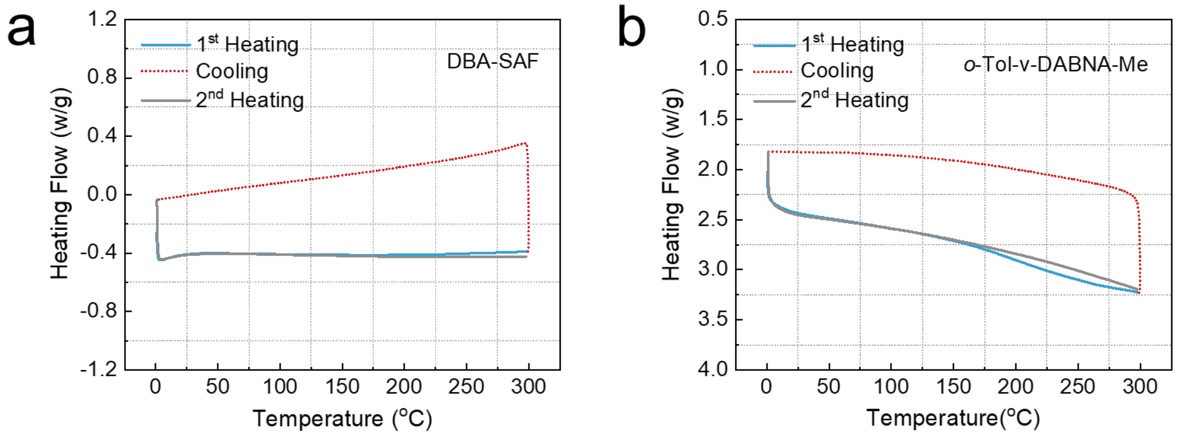

Figure S22. DSC measurements of (a) **DBA-SAF** and (b) ***o*-Tol-*ν*-DABNA-Me**, respectively.

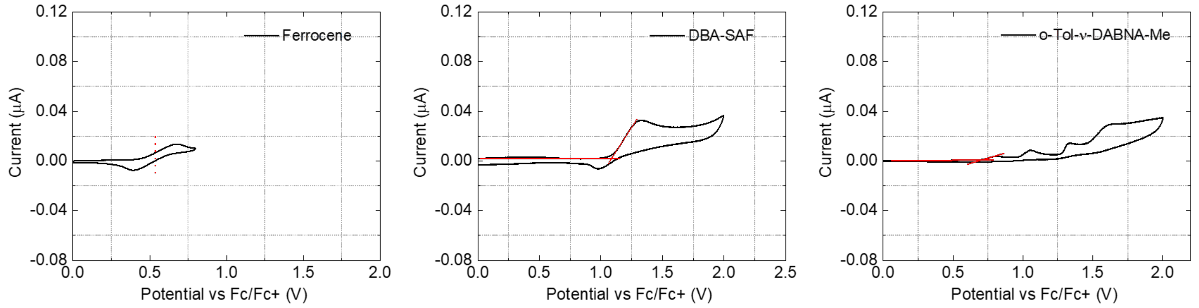

Figure S23. Cyclic voltammetry (CV) analysis of **DBA-SAF** (HOMO: - 4.97 eV) and ***o*-Tol-*ν*-DABNA-Me** (HOMO: - 5.36 eV), respectively. $\mathbf{HOMO\ (eV) = -4.8\ eV - [\mathit{E_{onset}} - \mathit{E_{1/2}}(\mathit{Ferrocence})]}$

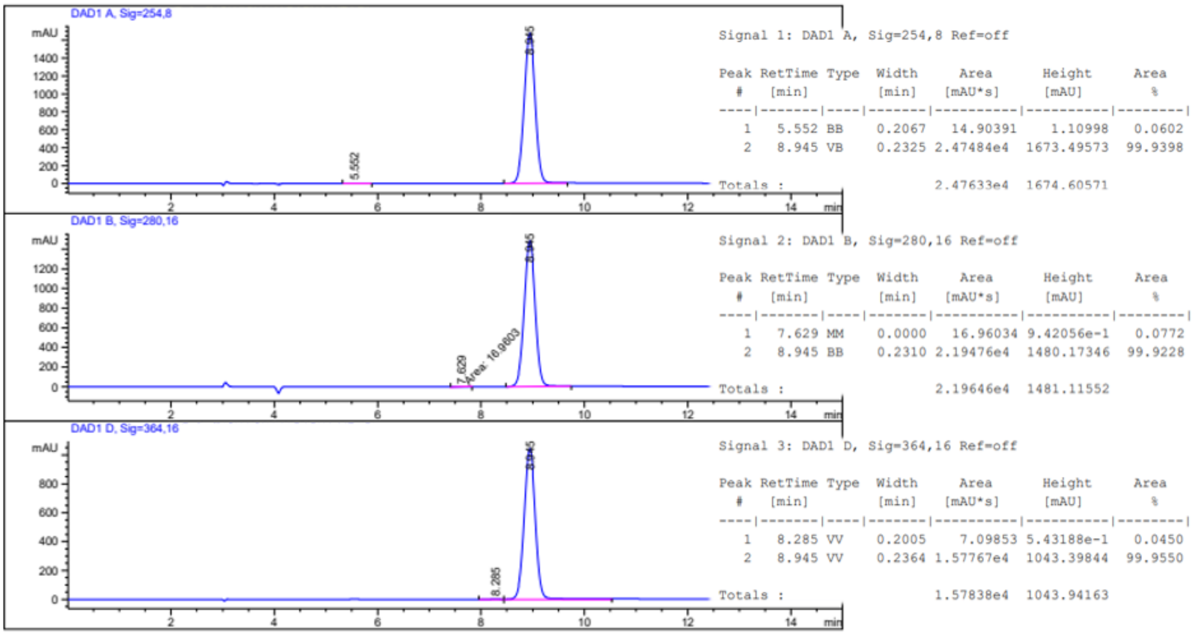

Figure S24. High-pressure liquid chromatography (HPLC) spectra of ***o*-Tol-*ν*-DABNA-Me**.

III. Computational details

Molecular Electronic-structure Calculation. To scrutinize the electronic structures of *o*-Tol-*v*-DABNA-Me, we first optimized the ground state (S_0) at the DFT level of theory using the B3LYP hybrid functional (*31-32*) with a valence double-ζ (DZ) polarized basis set (def2-SVP) (*33*) for all atoms. We then calculated vibrational frequency for the optimized geometry and confirmed no negative frequency. The electronic vertical absorption energies (E_{VA}) were calculated by employing a series of functionals and same basis set (def2-SVP) referenced from the S_0 geometry at the time-dependent DFT (TD-DFT) level within the Tamm-Dancoff approximation (TDA) (*34*); the first five singlet- and first five triplet excited states were taken into consideration, respectively. To obtain a good description of singlet and triplet excited CT states, two types of long-range corrected exchange functionals were introduced [i.e., Perdew-Burke-Ernzerhof exchange functional (LC-*w*PBE) (*35-36*) and *w*B97X functional with a van der Waals correction (*w*B97xD) (*37-38*)]. We thus adjusted the range separation parameter (*w*): the division ratio for exchange-correlation divided into a short-range domain (the first term) and a long-range part (the second term) in **Eq. S1**. (*39-40*)

$$\frac{1}{r_{12}} = \frac{1-[\alpha+\beta\cdot\operatorname{erf}(wr_{12})]}{r_{12}} + \frac{\alpha+\beta\cdot\operatorname{erf}(wr_{12})}{r_{12}}$$

Eq. (S1)

α [or β] quantifies the HF exchange contribution [or the DFT counterpart] over the whole range by a factor of α [or $1-(\alpha+\beta)$]. In conclusion, a three-parameter (α, β, and w) in **Eq. S1** of the two-electron operator ($1/r_{12}$) can be used for the separation of exchange term into each range-dependent domain (so-called Coulomb-attenuating method). (*39-40*) At this stage, we need to find the optimal range separation point (w^*) for the reliable CT excited-state properties. We thus utilized the concept based on Koopmans' theorem approximately applicable to the (generalized) Kohn-Sham calculation; the HOMO value can be identical to the minus vertical ionization potential (IP) when we neglect the orbital relaxation. (*41*) Furthermore, it can be considered not only IP but the electron affinity (EA) at the *N* (*N*+1) electron system at the target molecule for the rigorous calculation as show in **Eq. S2**. (*37*)

$$J^2(w) = \sum_{i=0}^{1} |\varepsilon^{w}_{\text{HOMO}}(N+i) + E^{w}(N-1+i) - E^{w}(N+i)|^2$$

Eq. (S2)

The geometries in S_1 and T_1 excited states of target material were optimized (i.e., adiabatic) using B3LYP/def2-SVP to investigate emission properties at the TD-DFT level. For the rigorous calculation, we also obtained T_1 state geometry of *o*-Tol-*v*-DABNA-Me via spin-unrestricted SCF calculation. (*15*) Herein, we did not consider any symmetry constraint for excited state geometry optimization. We then calculated vibrational frequency for the optimized geometry and confirmed no negative frequency. For an accurate ΔE_{ST} estimation of our target molecule, we introduced the state-of-the-art similarity transformed equation of motion coupled cluster singles and doubles (STEOM-CCSD) calculation. Particularly, the domain-based local pair natural orbital (DLPNO) theory-

based STEOM-CCSD applicable to an accurate excited energy level for a large molecule was used (HF/def2-SVP). (*42-43*) As a result, we obtained the converged S_1 (2.882 eV) and T_1 (2.867 eV) referenced from the optimized ground geometry at B3LYP/def2-SVP at DFT level. The percentage active character of each excited state was ~ 96 %, which means that the target roots (i.e., S_1 and T_1) are converged. And, we performed natural transition orbital (NTO) analyses to investigate the nature of excited states. (*44*)

Spin-orbit coupling matrix element (SOCME). We treated SOC as a perturbation based on the pseudo-relativistic orbitals after SCF and TDDFT calculation (so-called pSOC-TDDFT), (*45*) applicable to the molecular system composed of the light atoms (e.g., H, B, C, and N atoms in this study). We then calculated SOCME between S_1 and T_1 state, $\langle S_1|\hat{H}_{SOC}|T_1\rangle$, referenced from the optimized T_1 geometry. In the case of T_1 state geometry of *o*-Tol-*ν*-DABNA-Me, the spin-unrestricted SCF calculation was performed by using B3LYP/def2-SVP at UDFT level. In this calculation, we carried out the pSOC-TDDFT approach, using the B3LYP/double-ζ polarized the zero-order regular approximation (ZORA) (*46-47*) basis set for all atoms (DYALL-2ZCVP_ZORA-J-PT-GEN) (*48*) within TDA.

Excimer binding energy calculation. Given that the representative hybrid functional, B3LYP, underestimates Van der Waals interactions (e.g., the dispersion and dipole-dipole bound system) in general, we used the B3LYP-MM, (*21*) a posteriori-corrected functional, which accounts for non-covalent interactions by a series of empirical corrections. Those corrections depend on Cartesian coordinates of atoms (i.e. the geometry of target molecule) as well as the types of atoms rather than the electronic density of target molecule. We can add those empirical corrections after the regular DFT has been performed as described in **Eq. S3**. (*21*)

$$E_{B3LYP-MM} = E_{B3LYP} + E_{LDC} + E_{HBC} + E_{\pi^+}$$

Eq. (S3),

wherein E_{LDC}, E_{HBC}, and E_{π^+} denote the terms for Lennard-Jones-like correction to account for dispersion and induction effects, a hydrogen bonding correction, and a cation-pi correction, respectively. The use of B3LYP-MM functional has been reported to be more accurate for describing the type of interactions such as Van der Waals and hydrogen bonding than B3LYP-D3 and M06-2X functionals. (*21*) Hereby, we thus computed interaction energy of excimer sets extracted from the equilibrated frame in MD simulation by using B3LYP/LACVP* at DFT level. Further, we considered the compensation for the basis set superposition error (BSSE) to obtain accurate interaction energy in this study.

All the calculation methods described were performed using Gaussian 16, (*49*) MATERIALS SCIENCE SUITE quantum chemical and DFT package Jaguar developed by Schrödinger, (*50*) ORCA, (*51*) and wave function software Multiwfn. (*52*)

Molecular dynamic simulation (MD). We made the physically accessible disordered system based on an empirical force-field (OPLS4) for MD simulation. To do so, the molecular simulation box by populating molecules with a uniform probability distribution was built for each EML system: *ν*-DABNA derivatives doped on DBFPO host from 3 wt.% to neat state, Hyperfluorescent (HF) ternary system, and DBFPO host, but the

structures with rotatable bonds are rebuilt, using a self-avoiding random walk algorithm implemented in Desmond.(*18-19*) Herein, we used the periodic box (the number of particles, *N*, 1024 molecules) for MD simulation defining the interatomic interactions. And, we carried out MD simulation by running an *NPT* (the isothermal-isobaric ensemble) for 100 ns using Nosé-Hoover chain thermostat @ 300 K and Martyna-Tobias-Klein (*MTK*) barostat @ 1.013 bar with isotropic pressure coupling after the relaxation protocol: (i) 20 ps *NVT* (the canonical ensemble) Brownian minimization @ 10 *K*, (ii) 20 ps *NPT* Brownian minimization @ 100 *K*, then (iii) 100 ps *NPT* MD stage @ 100 *K*. We confirmed the convergence and used the trajectories collected from the last 20 ns for the analyses in this study.

IV. Exciton dynamics

The Exciton Lifetime Fitting. The transient PL intensities (*I, counts*) are fitted with the sum of exponential decay of the following expression;

$$I\ (counts) = \sum_{l=1}^{n} I_{\mathrm{n}} e^{-\frac{t}{\tau_n}} + I_{\mathrm{IRF}}$$

Eq. (S4)

Hereby, I_{n} is the relative contribution of the individual decays with lifetimes (τ_{n}) and I_{IRF} is a constant offset, corresponding to an instrument response function (IRF) in solution and solid-states.

TADF kinetics for an Optical/Electrical Model. From the three-level model (optical), (*25*,*54*) we can simply define an electrical model by adding a series of term: $\frac{J}{4qd} - G$ and $\frac{3J}{4qd}$ in **Eqs. S5** and **S6**, respectively.

$$\frac{dN_{\mathrm{S}}(t)}{dt} = -(k_{\mathrm{r}}^{\mathrm{s}} + k_{\mathrm{nr}}^{\mathrm{s}} + k_{\mathrm{ISC}})N_{\mathrm{S}}(t) + k_{\mathrm{RISC}}N_{\mathrm{T}}(t) + G$$

Eq. (S5)

$$\frac{\mathrm{d}N_{\mathrm{T}}(t)}{dt} = -(k_{\mathrm{nr}}^{\mathrm{T}} + k_{\mathrm{r}}^{\mathrm{T}} + k_{\mathrm{RISC}})N_{\mathrm{T}}(t) + k_{\mathrm{ISC}}N_{\mathrm{S}}(t)$$

Eq. (S6)

Where G is the singlet exciton generation rate from the photon absorption in the system, J is the current density, q is the elementary charge, and d is the recombination zone thickness, respectively.

Roll-off Model Analysis. Hereby, we can express the singlet and triplet exciton density under the electrical system when the bi-excitonic annihilations, STA and TTA, dominantly occurs in TADF based OLED devices: (*23-24*)

$$\frac{dN_{\mathrm{S}}(t)}{dt} = -(k_{\mathrm{r}}^{\mathrm{s}} + k_{\mathrm{nr}}^{\mathrm{s}} + k_{\mathrm{ISC}})N_{\mathrm{S}}(t) + k_{\mathrm{RISC}}N_{\mathrm{T}}(t) - k_{\mathrm{STA}}N_{\mathrm{S}}(t)N_{\mathrm{T}}(t) + \frac{1}{8}k_{\mathrm{TTA}}N_{\mathrm{T}}^{2}(t) + \frac{J}{4qd}$$

Eq. (S7)

$$\frac{\mathrm{d}N_{\mathrm{T}}(t)}{dt} = -(k_{\mathrm{nr}}^{\mathrm{T}} + k_{\mathrm{r}}^{\mathrm{T}} + k_{\mathrm{RISC}})N_{\mathrm{T}}(t) + k_{\mathrm{ISC}}N_{\mathrm{S}}(t) - \frac{5}{8}k_{\mathrm{TTA}}N_{\mathrm{T}}^{2}(t) + \frac{3J}{4qd}$$

Eq. (S8)

Where k_{STA} [k_{TTA}] denotes the rate constant [$\mathrm{cm}^3\ \mathrm{s}^{-1}$] of STA [TTA] process, respectively. In this model, we factored in the singlet production ratio (α) with 0.25 (i.e., following spin-statistics), T_1 exciton exothermic reaction from TTA by a factor of half (0.5), and the triplet attenuation ratio of -5/8 (refer to **Eq. S8**).

We re-fitted the experimental data for 4,5-di(9*H*-carbazole-9-yl)phthalonitrile [2CzPN] based OLEDs reported by Masui et al. (*23*) by our analytical model to verify fitting rigorousness. From the results, we got the comparable values of $k_{TTA} = 8.2 \times 10^{-15}$ cm^3 s^{-1} and $k_{STA} = 1.6 \times 10^{-11}$ cm^3 s^{-1} (*ceteris paribus* in this calculation, excluding the triplet attenuation ratio of -5/4) [reference value, $k_{TTA} = 5 \times 10^{-15}$ cm^3 s^{-1} and $k_{STA} = 2 \times 10^{-11}$ cm^3 s^{-1}].

V. The calculated electronic properties of *o*-Tol-*v*-DABNA-Me

To investigate the nature of CT electronic structures for target material, at this stage, we performed QM calculations using the representative density functional theory (DFT) functionals and the state-of-art post-Hartree-Fock (HF) method presented in **Figures S25**a and b. In general, the hybrid functionals, such as B3LYP (20% HF) and PBE0 (25% HF), underestimate the conventional D-A type CT excitation energy. (*15*) We thus performed long-range corrected functionals (*w*B97x-D and LC-*w*PBE) with optimal range-parameter *w** ─LC-*w**PBE (0.1197 Bohr^{-1}), *w**B97x-D (0.0908 Bohr^{-1})─ based on Koopman's theorem (see 'Computational details' section and **Figure S26** for more details on the calculation). However, the vertical absorption excitations (E_{VA}) at gas-phase using various DFT functionals yield large ΔE_{ST} values in the range of ~ 0.33 – 0.50 eV (**Table S2**). This significant overestimation of ΔE_{ST} at the time-dependent DFT (TD-DFT) level is driven by the short-distance reshuffling of the electronic density at the excited states of the DABNA analog system, which could cause errors in that the conventional D-A CT type chromophores have a long-distance charge separation upon the excitation.

To describe those high-order electronic correlation effects correctly, we introduced the similarity transformed equation of motion coupled-cluster singles and doubles (STEOM-CCSD) method. (*42-43*) **Figure S25**a shows the natural transition orbital (NTO) behaviors for S_1 state reference from the ground geometry. Remarkable two main electronic transitions: (i) HONTO → LUNTO and (ii) HONTO-1 → LUNTO+1 occur mainly on π-extended skeleton rather than PPGs. In particular, the spatially short-distance delocalized multiple CT (^{de}CT) effects are dominantly made from the electron-rich nitrogen atoms part to boron-centered azatriangulene backbone for those contracted electronic transition cases. With this calculation, ΔE_{ST} value is estimated to be 0.015 eV, which is in good agreement with the experimental value of 0.01 eV.

The enhancement of ^{de}CT is attributed to the delocalized wavefunctions that result from the MR effect between hole/electron rich parts within a short distance. The CT distance (Δr) between the hole and particle for optimized S_1 (adiabatic) is given as 0.71 Å at π-extended ribbon skeleton. This results in a very small exchange energy (K) and thus ΔE_{ST}. It should be noted that this small ΔE_{ST} is achieved while maintaining a relatively large overlap between HOMO and LUMO ($I_{H/L}$) of 57.7%; this leads to a strong f_{osc} of 0.78 and thus high PLQY, which is supported by the increased polarizability along the long axis of the molecule (refer to the direction of the TDM vector as show in **Figure S29**). For 3 wt.% *o*-Tol-*v*-DABNA-Me doped DBFPO host, the rate constant of radiative singlet decay (k_r^S) and PLQY are estimated to be 2.77×10^8 s^{-1} and ~0.99 owing to the f_{osc} enhanced by the MR effect.

To deepen understating of spin-flip behaviors, we calculated spin-orbit coupling matrix element (SOCME, H_{SO}) at optimized T_1 geometry under the scalar relativistic level. The average H_{SO} between S_1 and T_n (n = 1-3) state was in order of 0.02, 0.06, and 0.08 cm^{-1}, respectively (**Table S3**). It is noteworthy that the such a small H_{SO} was obtained due to the similar MR-CT FMOs features between S_1 and T_1 state, leading to small orbital angular momentum change. (*15*) Therefore, the triplet uphill may occur via reverse internal conversion (rIC) have been proposed and under discussion. (*8*) From a little different perspective, under the framework of Fermi's golden rule, the theoretical kinetic prediction for the rate constant of RISC (k_{RISC}) can be formulated by introduction of Marcus theory. (*15*, *55*) **Figure S27** shows the theoretical behavior of k_{RISC} for the target molecule as a function of H_{SO} and ΔE_{ST} at fixed reorganization energy of 0.03 eV. Note that the predicted k_{RISC} value (@ H_{SO} = 0.02 cm^{-1}, ΔE_{ST} = 0.015 eV) was 3.10×10^5 s^{-1} is well-matched with the experimentally determined that of k_{RISC} (3.21×10^5 s^{-1}, 3 wt.% doped DBFPO host). Further, the k_{RISC} found around 2.1 – 3.2×10^5 s^{-1} at RT in a series of solutions and films supports the discussion thereof. And, the obtained k_{ISC} was in the range of 3.1 – 7.4×10^7 s^{-1} for specimens, which is consistent with the results of organic chromophores composed of light atoms (Z_n < 15).

Table S2. Single-point calculation results from the optimized ground state (o-Tol-ν-DAVNA-Me)

Item[a]	B3LYP [eV]	PBE0 [eV]	[b]LC-*w*PBE [eV]	[c]LC-*w**PBE [eV]	[b]*w*B97x-D [eV]	[c]*w**B97x-D [eV]	[d]STEOM-CCSD [eV]
S_1	2.947	3.060	3.607	2.895	3.570	3.202	2.882
T_1	2.617	2.696	3.103	2.532	3.113	2.820	2.867
$\Delta E_{ST,ver}$	0.331	0.364	0.504	0.363	0.457	0.381	0.015
f_{osc}	0.775	0.810	0.881	0.706	0.857	0.797	0.555

[a]Vertical excitation energies obtained from the ground state at DFT level (B3LYP/def2-SVP). [b]w = 0.3 $Bohr^{-1}$ utilized for the system. [c]An optimal $w*$ used for individual system. [d]The percentage active character of the converged S_1 and T_1 is ~ 96 %, respectively.

Table S3. Calculated adiabatic energies for excited states and SOCME for *o*-Tol-*ν*-DABNA-Me

$S_{1(Adia)}$[a]	$T_{1(Adia)}$[a]	$T_{1(Adia)}$[b]	HOMO[c]	LUMO[c]	[d]$H_{SO,T1}$	[d]$H_{SO,T2}$	[d]$H_{SO,T3}$
2.88 eV	2.54 eV	2.54 eV	-4.85 eV	-1.44 eV	0.02 cm^{-1}	0.06 cm^{-1}	0.08 cm^{-1}

[a]Optimized B3LYP/def2-SVP at TD-DFT level. [b]Triplet geometry obtained from Unrestricted DFT (UDFT) method. [c]at optimized ground state (B3LYP/def2-SVP). [d]SOCME values between S_1 and T_n (n = 3) at the T_1 geometry accessed by spin-relaxed open-shell optimization. The calculation based on B3LYP/ZORA basis set at the scalar relativistic level (i.e., pSOC-TDDFT within TDA).

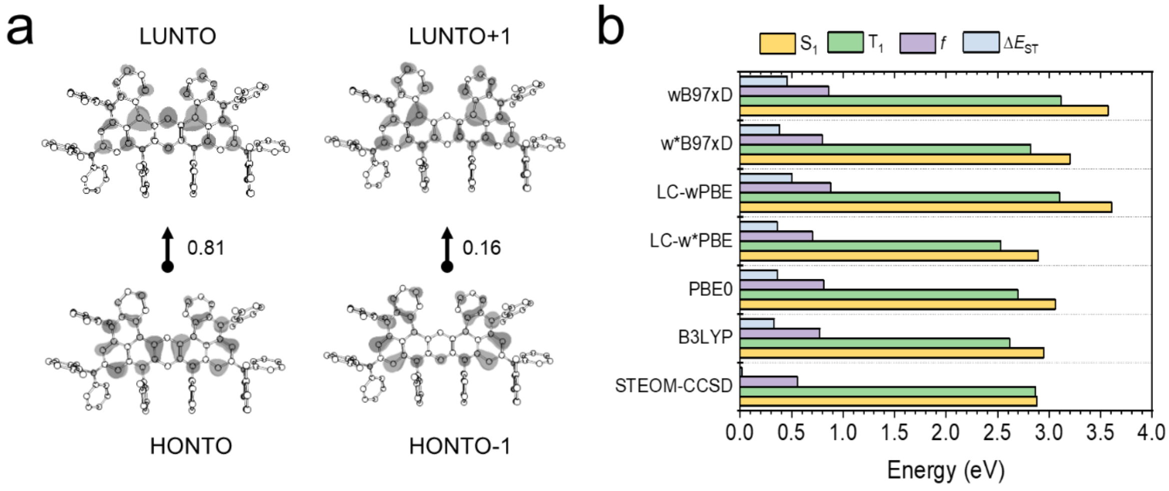

Figure S25. Quantum chemical calculations (a) NTO behaviors at S_1 using a post-HF method [domain-based local pair natural orbital theory (DLPNO) based STEOM-CCSD/def2-SVP]. (b) The vertical excitation energy (E_{VA}) for S_1 and T_1 state of *o*-Tol-*ν*-DABNA-Me.

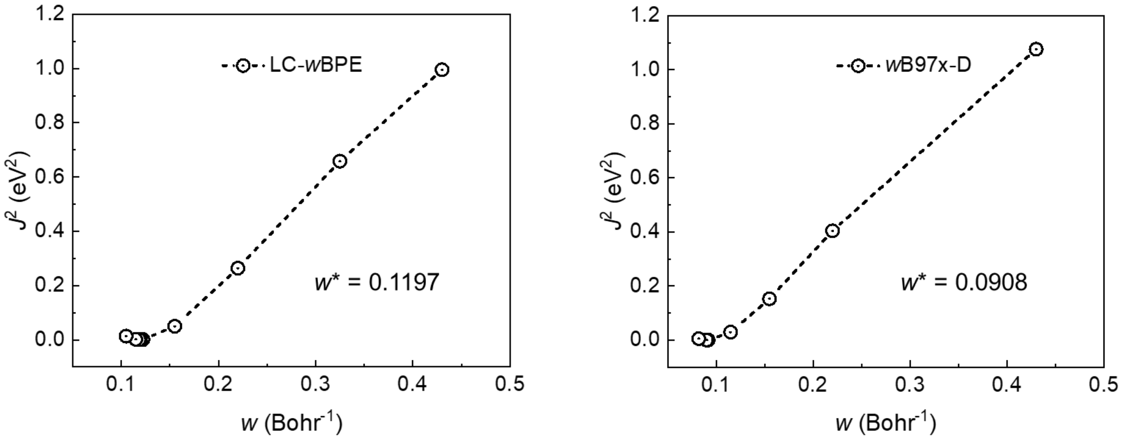

Figure S26. Optimal *w** found using long-range corrected functionals (LC-*w*BPE and *w*B97x-D) and basis set (def2-SVP) referenced from the optimized ground state geometry of *o*-Tol-*ν*-DABNA-Me (B3LYP/def2-SVP) at DFT level.

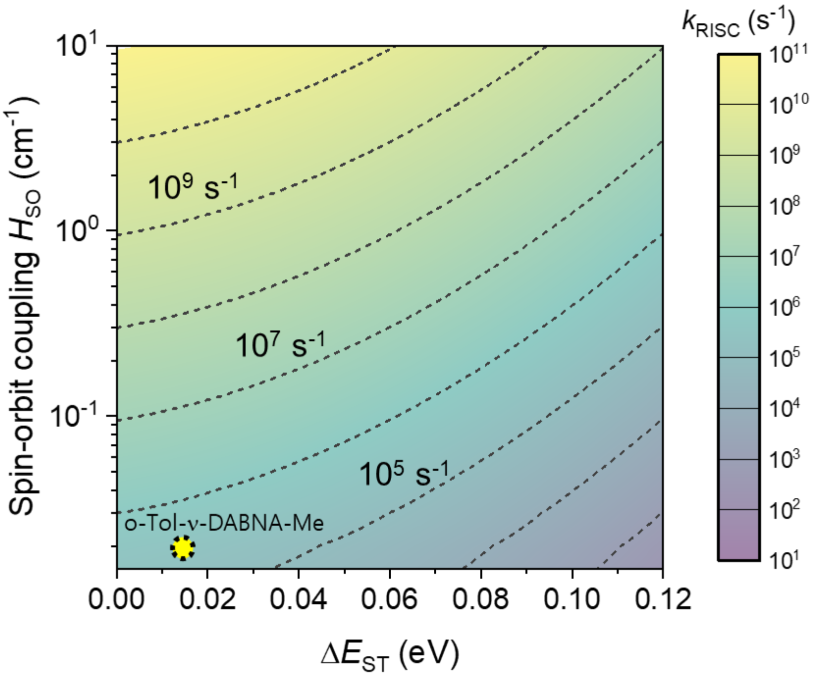

Figure S27. k_{RISC} as a function of H_{SO} and ΔE_{ST} with fixed reorganization energy (0.03 eV).

VI. Photophysical Study

Figure S28a shows the room-temperature PL (RTPL) and low-temperature PL (LTPL) curves for *o*-Tol-*ν*-DABNA-Me diluted in toluene after 74 ms of a delay from the excitation. Interestingly enough, the resemblance between RTPL and LTPL curve indicates that S_1 and T_1 have similar MR-CT features, resulting in a very small ΔE_{ST} of ~ 0.01 eV. To further characterize its MR-CT effect, we recorded a series of transient-PL (Tr-PL) profiles in solution and in its condensed state as shown in **Figures S28**b and c, respectively. In contrast to the conventional CT-type materials, a solvatochromism has little influence on the absorption and emission profiles of *o*-Tol-*ν*-DABNA-Me; there was almost no change in peak wavelength (λ_{peak}) and the exciton decay lifetime (both prompt and delayed). This was also the case for the absolute PLQY (Φ_{PLQY}) except for a slight increase with the solvent polarity. This is because the transition dipole moment (TDM) of *o*-Tol-*ν*-DABNA-Me for the transition: $S_1 \rightarrow S_0$ ‒ [X, Y, Z] = [-3.91, -0.10, 0.17] ‒ lies along the 5-membered ring plane (i.e., the long axis of the molecule) as shown in **Figure S29**. In other words, solvent-induced dipole moment change is orthogonal to the TDM vector, and thus a solvatochromic effect on TDM is negligible in this case. Based on the measured PL emission spectra shown in **Figure S30**, we observed that the solvent polarity affects the MR-CT character of S_1 state but a small degree. For example, when one changes the dielectric constant of solvent from that of cyclohexane (ε = 2.02) all the way to that of acetonitrile (ε = 36.64), only a slight red-shift up to 7.4 nm was measured, indicating that the solvatochromic shift is not prominent in this work.

Table S4. Photo-physical properties of o-Tol-ν-DAVNA-Me

State	λ_{peak} (nm)	PLQY[a]	τ_{PF} (ns)	τ_{DF} (μs)	k_r^S ($\times 10^8$ s^{-1})	k_{ISC} ($\times 10^7$ s^{-1})	k_{RISC} ($\times 10^5$ s^{-1})	Φ_{DF}/Φ_{PF}	Chi^2
Toluene (0.05 mM)	466	0.92 (0.58)	4.59	1.72	1.87	3.09	2.95	0.072	1.05
THF (0.05 mM)	468	0.89 (0.49)	4.56	1.68	1.85	3.42	2.08	0.055	1.00
DPEPO (3 wt.%)	472	0.89	2.94	3.15	2.66	7.40	2.01	0.138	1.04
DBFPO (3 wt.%)	472	0.99	3.04	3.68	2.77	5.21	3.21	0.187	1.13
DPEPO (5 wt.%)	473	N/A	2.98	3.07	N/A	N/A	N/A	N/A	0.95
DBFPO (5 wt.%)	473	N/A	3.09	2.28	N/A	N/A	N/A	N/A	1.08

[a]Parenthesis means that the measured PLQY at an aerated system.

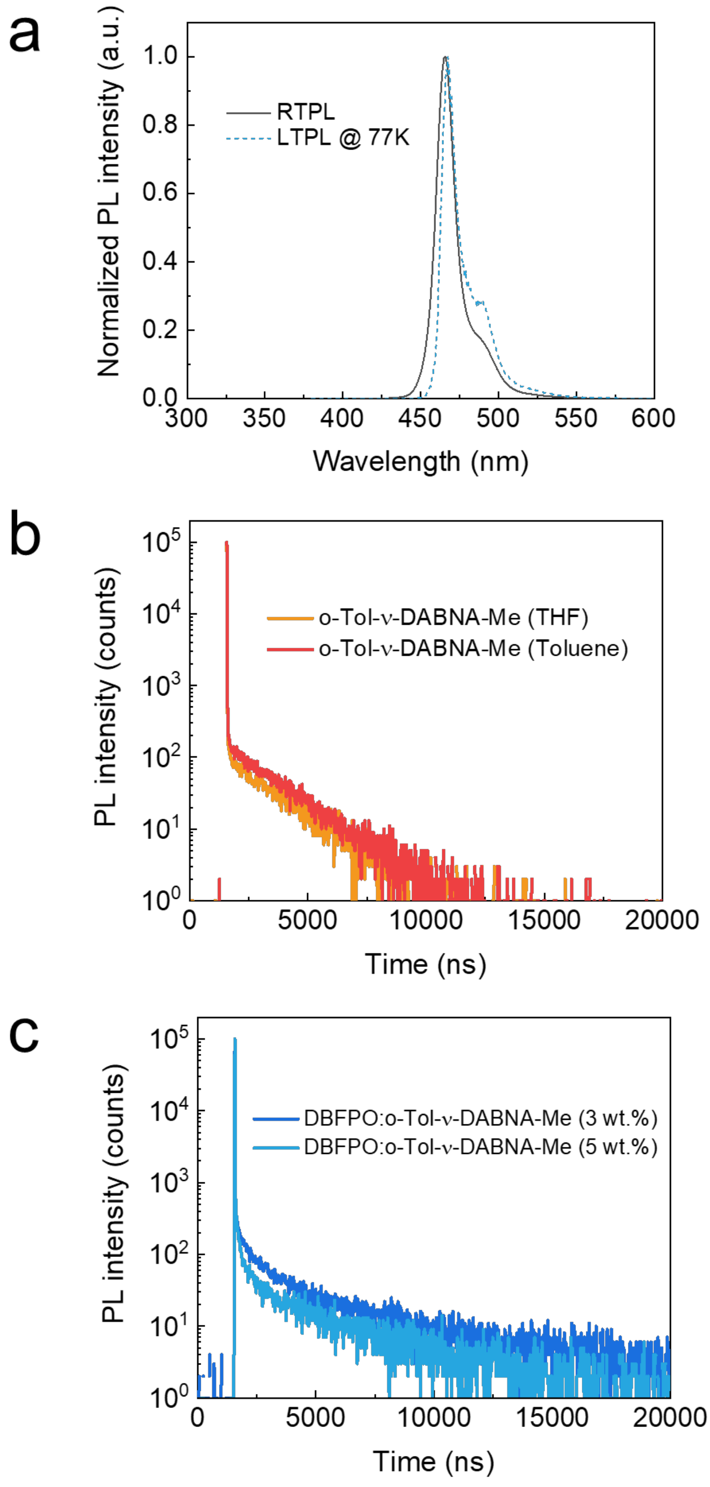

Figure S28. Photophysical properties (a) RTPL and LTPL profiles for target material at 77K in toluene. (b) Tr-PL profiles of *o*-Tol-*ν*-DABNA-Me in solutions (0.05 mM). (c) The representative Tr-PL decay profiles for target material in the condensed solid-state.

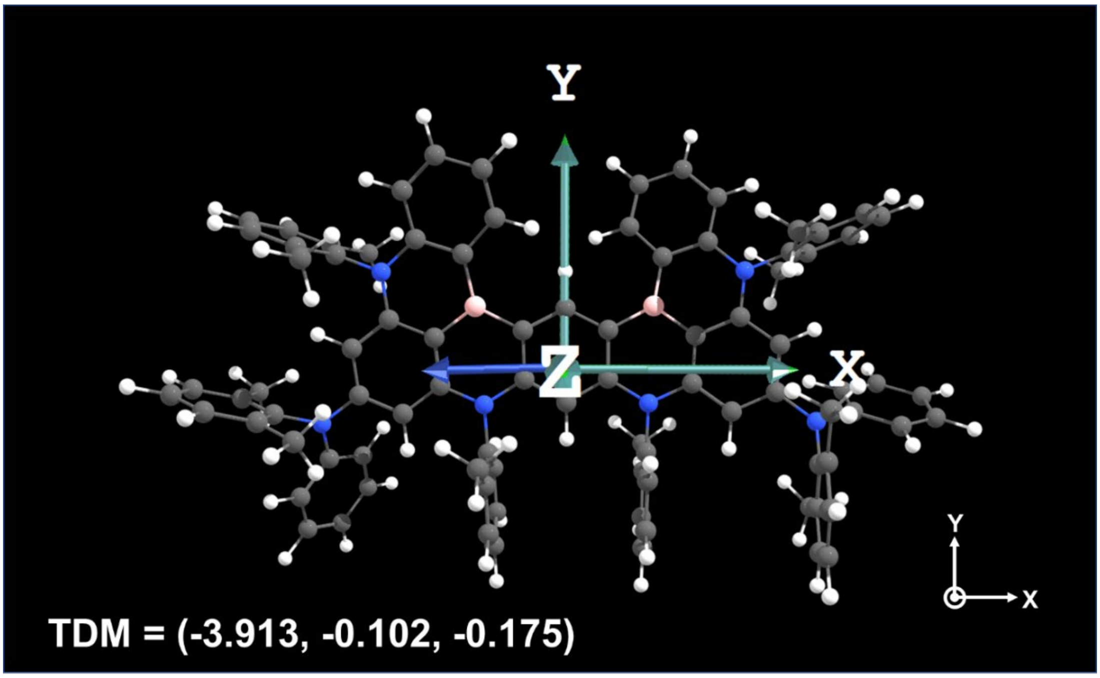

Figure S29. The transition dipole moment (TDM) vector for *o*-Tol-*v*-DABNA-Me from the electronic transition: $S_1 \rightarrow S_0$. The single-point calculation is done by B3LYP/def2-SVP @ TD-DFT level reference from the optimized excited singlet state (S_1).

To further support our statement that the TDM vector keeps its orientation in the plane of the DABNA ribbon along the long axis even in solution phase, we employed the Poisson-Boltzmann continuum solvation model using the optimized S_1 geometry of *o*-Tol-*v*-DABNA-Me in the gas phase as the reference energy. As shown in **Figures. S31**a and b, we can confirm that the solvation energy is proportional to solvent polarity, indicating that the molecule can be readily dissolved into solvent as the dielectric constant increases. Interestingly, the calculated vertical emission energy (E_{VE}) in **Figure. S31**c and the oscillator strength (*f*) in **Figure. S31**d show little differences compared to those in the gas phase. This suggests that the solvent re-orientation might not induce electronic relaxation of S_1 state, and that the TDM vector is orthogonal (insensitive) to the solvent-induced dipole moment change. In summary, our results demonstrate that the solvation effect affects the electronic properties of *o*-Tol-*v*-DABNA-Me only weakly and that the TDM vector remains almost unchanged upon solvation.

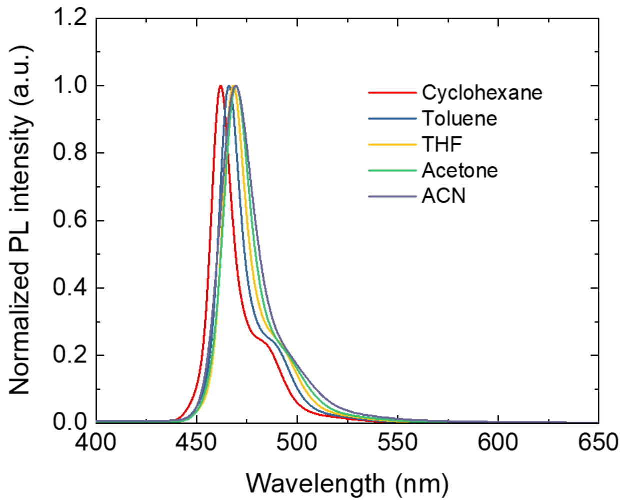

Figure 30. Solvation effect on *o*-Tol-ν-DABNA-Me at the concentration of 5×10^{-5} M. The main peak wavelength of *o*-Tol-ν-DABNA-Me dissolved at each solvent is as follows; Cyclohexane (462.0 nm), toluene (466.2 nm), tetrahydrofuran (THF) (468.2 nm), acetone (469.6 nm), and acetonitrile (ACN) (469.4 nm).

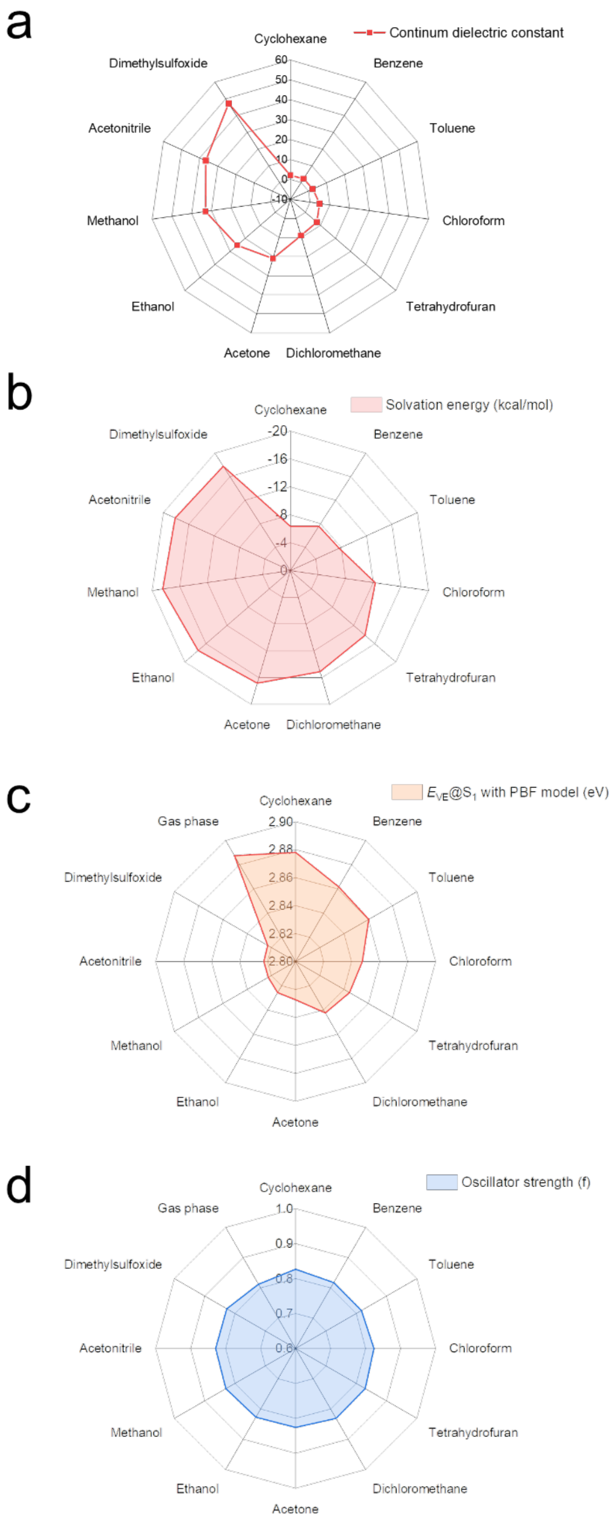

Figure S31. The continuum dielectric constant for a series of solvents. (b) The calculated solvation energy (kcal/mol) for *o*-Tol-*ν*-DABNA-Me molecule. (c) The calculated E_{VE} and (d) oscillator strength (*f*) referenced from the optimized S_1 geometry (gas phase). For the consideration of solvation effect, we used Poisson-Boltzmann continuum solvation model.

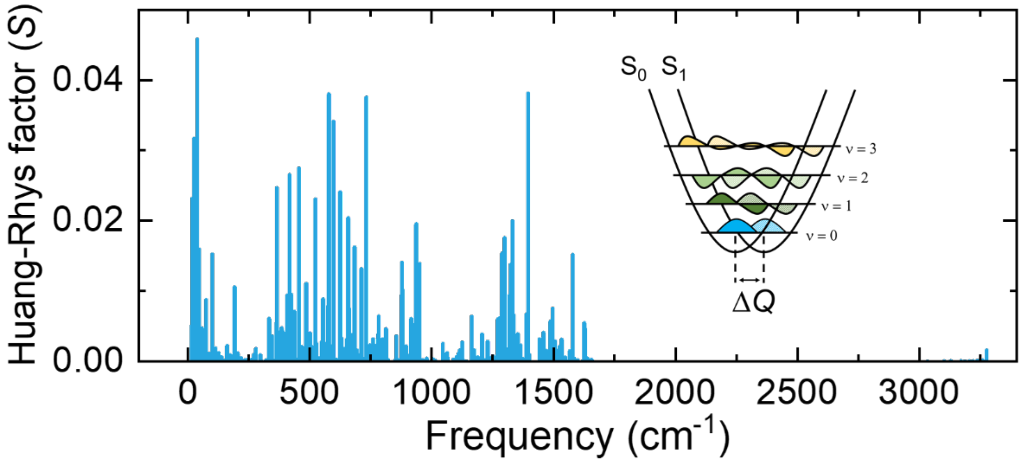

Figure S32. The Huang-Rhys (HR) factor (S_i) factorized into reorganization energy (λ_S) for *o*-Tol-*ν*-DABNA-Me system. The normal coordinates (*Q*) of two electronic states (i.e., the optimized S_1 and S_0 states) can be described by a linear transformation under the harmonic approximation; Q (at S_0) = JQ (at S_1) + K where J is Duschinsky matrix and K is the equilibrium position displacement vector of two electronic states. To obtain the equilibrium geometries and vibration frequencies in each electronic state, we performed a series of DFT calculations at B3LYP/def2-SVP level.

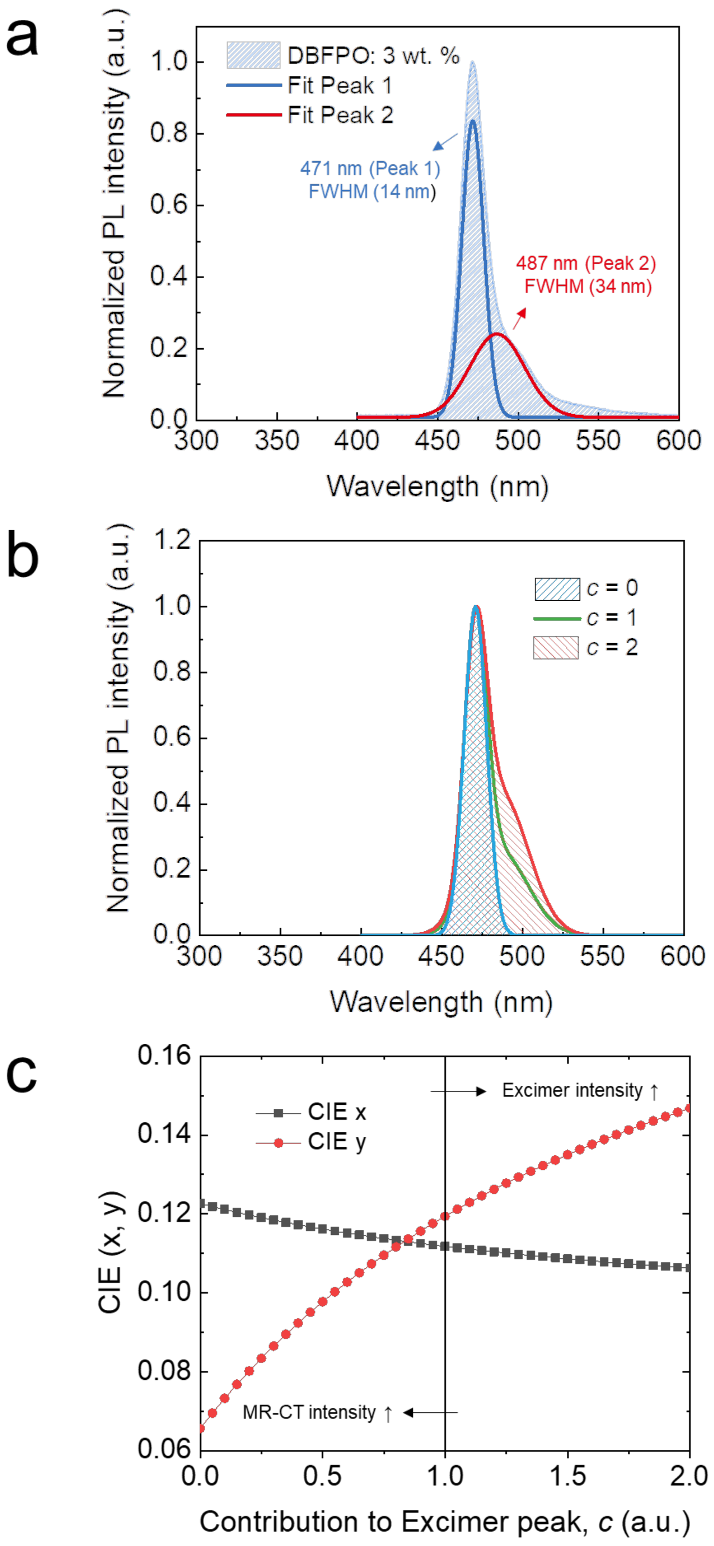

Figure S33. The contribution from λ_{side} (a) The deconvoluted PL profile of 3 wt.% doped DBFPO host (b) The PL profiles by an increment of excimer PL profile (c) CIE (x, y) *vs* contribution to Excimer peak.

Table S5. The interaction event counts (median, counts/mol) obtained from the equilibrated amorphous-state

Item	DBFPO	3 wt.%	5 wt.%	10 wt.%	20 wt.%	40 wt.%	HF
π-π interactions ($\times 10^{18}$ Counts/mol)	1.47	1.43	1.40	1.35	1.22	0.99	1.56

[a]To quantify π-π interactions, the face-to-face geometry wherein the cut-off point is defined as the maximum distance between centroids of the rings with 4.4 Å and the maximum angle between the ring planes with 30°. In the case of the edge-to-face geometry, we used the cut-off point that includes the maximum distance between centroids of the rings with 5.5 Å and the minimum angle between the ring planes with 60°.

It is noted that the quantified π-π interaction events decrease in gradual from DBFPO host (1.47×10^{18} *counts/mol*) to 40 wt.% doped case (9.91×10^{17} *counts/mol*) in **Table S5** since the number of non-bonding interactions made from the intermolecular host molecules is more probable than those of dopants. Surprisingly, the cohesive energy (E_{coh}) change in accordance with doping ratio, the intermolecular non-bonding energy (e.g., Van der Waals, electrostatics, and hydrogen bonding), from each MD simulation connotes that the face-to-edge configuration of excimers are more favorable to occur in the high-doping ratio for *ν*-DABNA clusters.

VII. Molecular Anisotropy

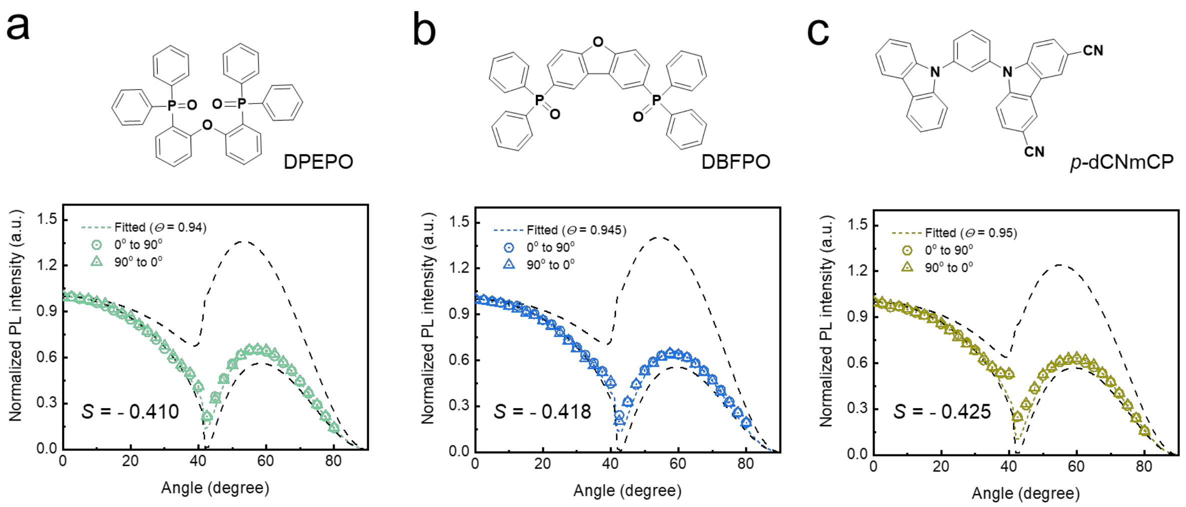

Figure S34. Angular-dependent *p*-polarized PL profiles for *o*-Tol-*ν*-DABNA-Me (5 wt.% doped) for (a) DPEPO, (b) DBFPO, and (c) *p*-dCNmCP blue host candidates, respectively. (The film thickness: 50 nm).

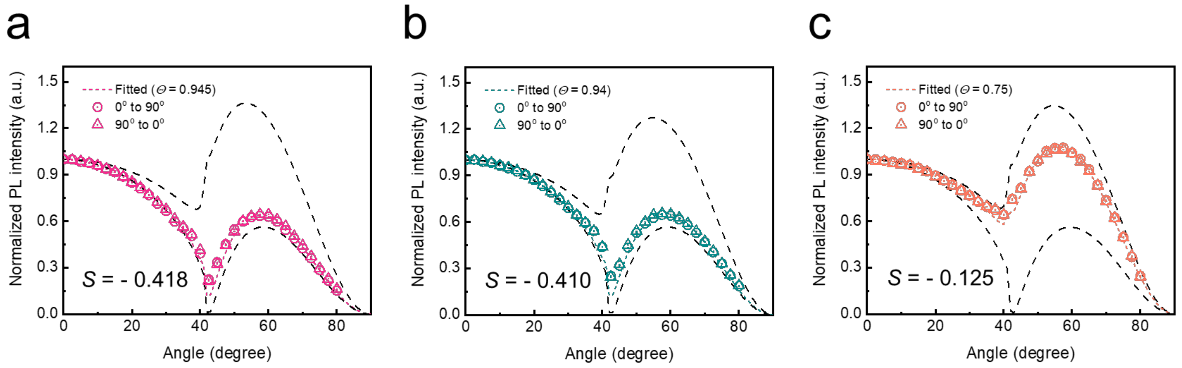

Figure S35. Angular-dependent *p*-polarized PL profiles for (a) *o*-Tol-*ν*-DABNA-Me (3 wt.% doped) for DPEPO host, (b) *o*-Tol-*ν*-DABNA-Me (10 wt.% doped) for DBFPO host, and (c) DBA-SAF (30 wt.% doped) for DBFPO host, respectively. (The film thickness: 50 nm).

VIII. Electrostatic potentials (ESP) mapping

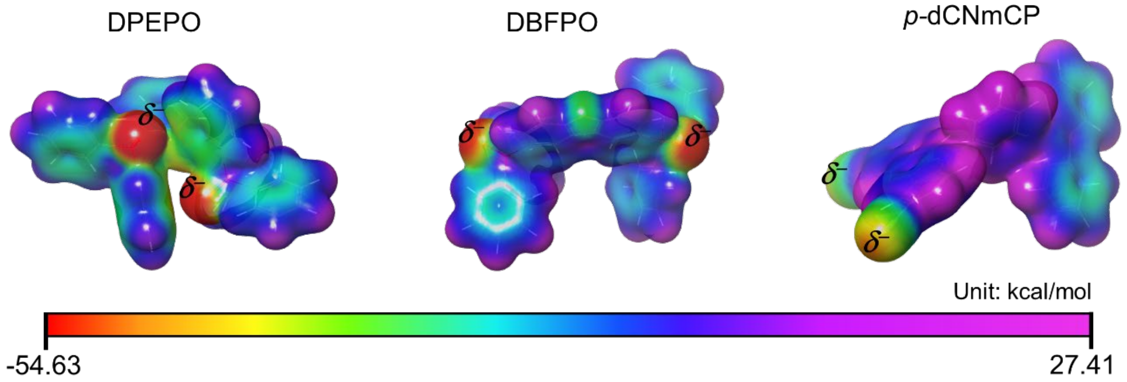

Figure S36. Electrostatic potentials (ESP) of DPEPO, DBFPO, and *p*-dCNmCP host molecules. Herein, we used the optimized ground state geometry using B3LYP/6-31G** at DFT level. And, ESP is mapped on the isosurface of electron density (0.005 electrons/bohr^3). A measure of charge separation for DPEPO, DBFPO, and *p*-dCNmCP molecules for 10.96, 9.89, and 11.09 kcal/mol, respectively.

IX. Device performance

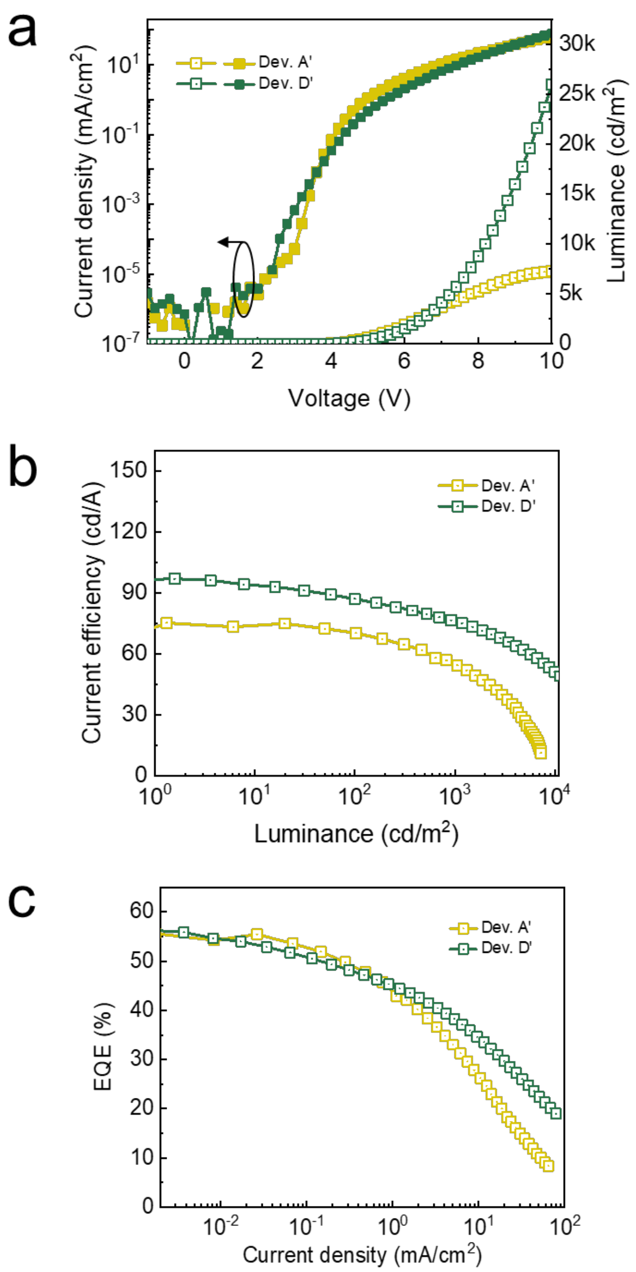

Figure S37. Device performance (a) *J-V-L* characteristics (b) luminance-efficiency curves, and (c) EQE vs current density for **Dev. A'** and **Dev. D'**, respectively.

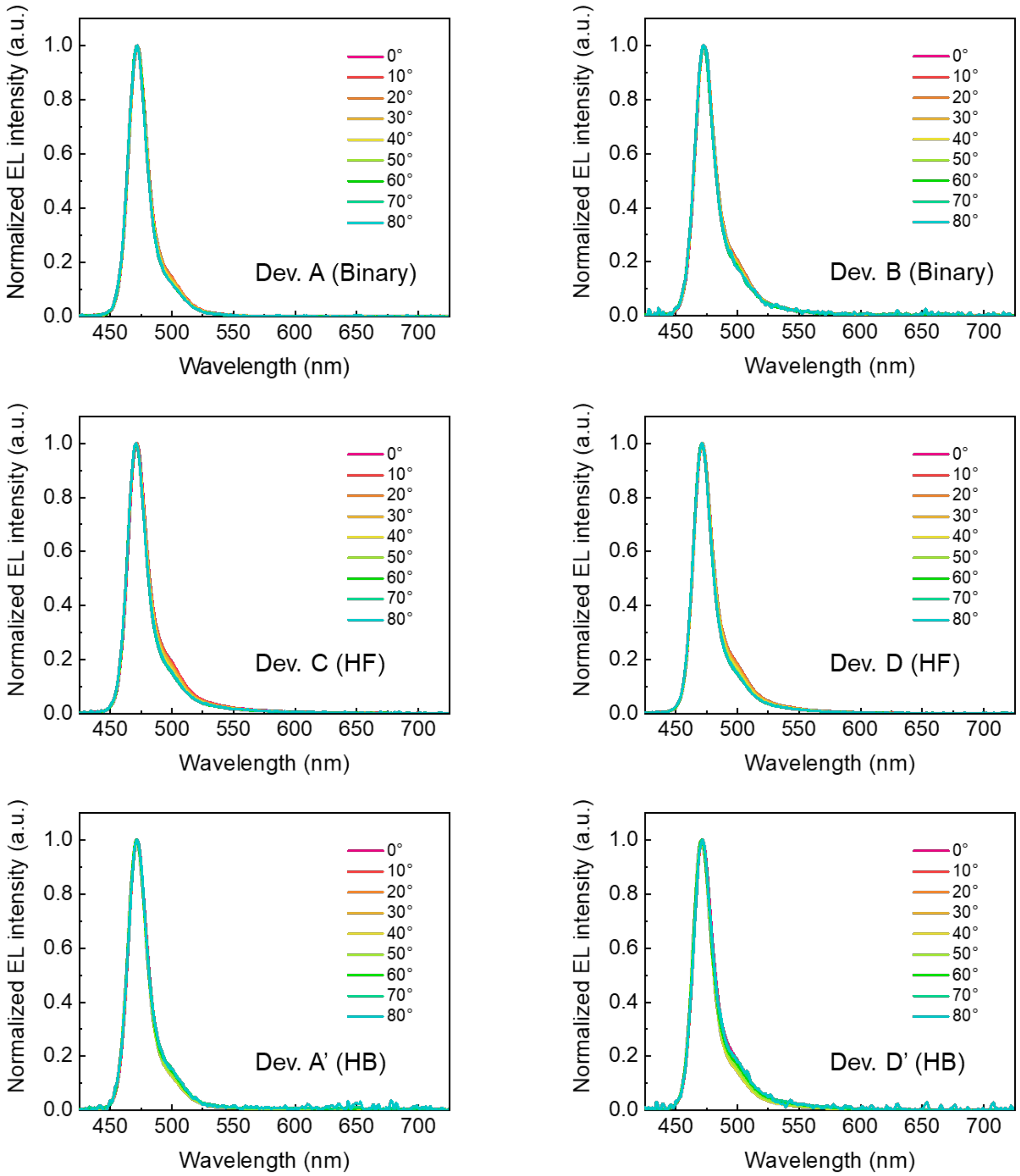

Figure S38. Angular EL spectra for a series of tested devices in this study (in steps of 10º).

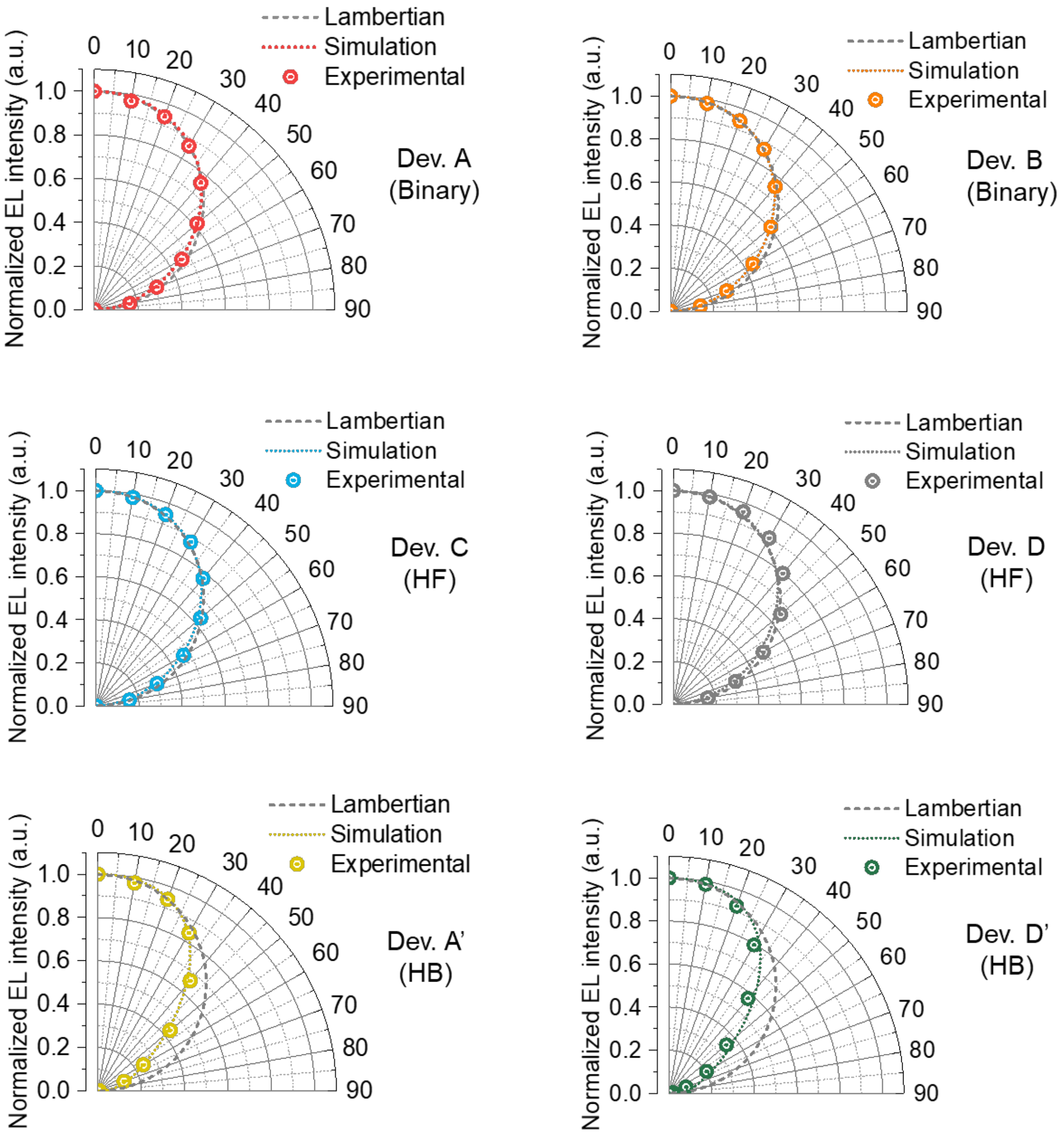

Figure S39. The angle dependent EL emission pattern of a series of tested devices.

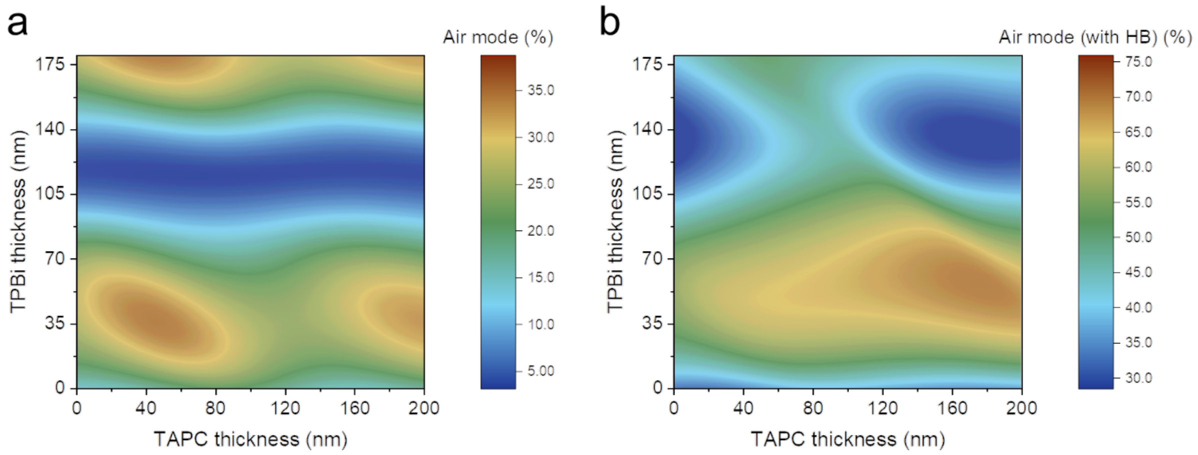

Figure S40. Outcoupling efficiency (η_{OC}) contour plots for (a) **Dev. A** and (b) **Dev. A'** as functions of HTL versus ETL thickness for the proposed device architecture. (PLQY:0.9, Θ_h:0.945, fixed-dipole model)

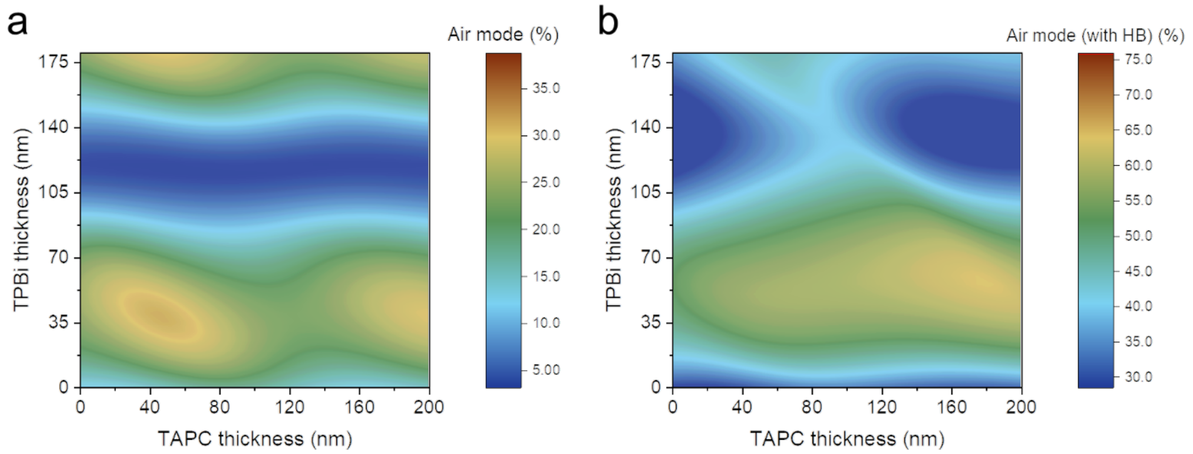

Figure S41. Outcoupling efficiency (η_{OC}) contour plots for (a) **Dev. B** and (b) **Dev. B'** as functions of HTL versus ETL thickness for the proposed device architecture. (PLQY:0.8, Θ_h:0.94, fixed-dipole model)

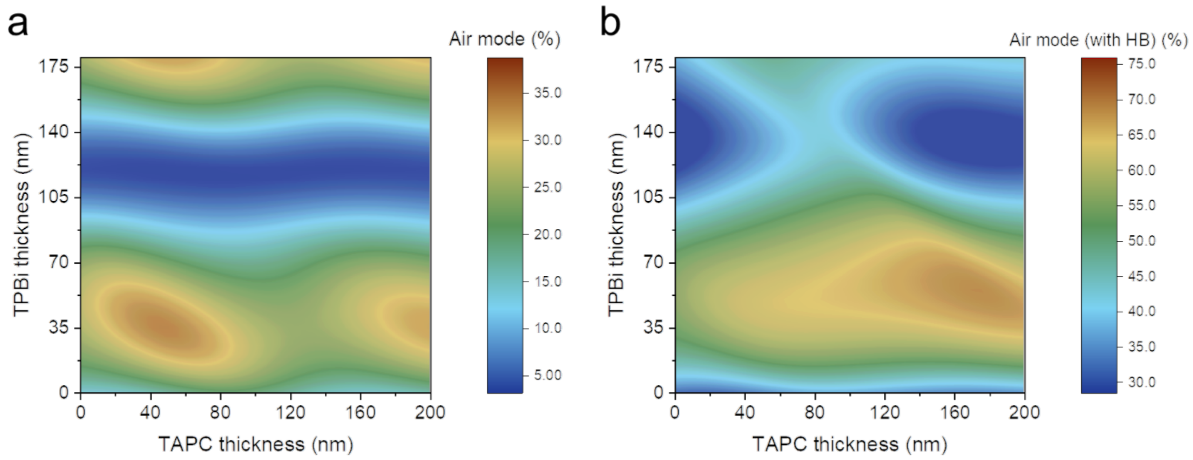

Figure S42. Outcoupling efficiency (η_{OC}) contour plots for (a) **Dev. D** and (b) **Dev. D'** as functions of HTL versus ETL thickness for the proposed device architecture. (PLQY:0.9, Θ_h:0.94, fixed-dipole model)

X. Hyperfluorescent (HF) design

A fundamental understanding of the DABNA-core molecule and its impact on OLED device performance is mostly unexplored. In this context, we showed proof-of-concept HF study by use of not only a terminal fluorescent dopant for *o*-Tol-*v*-DABNA-Me but also co-deposited TADF emitter as an assistant dopant, 10-(5,9-dioxa-13b-boranaphtho[3,2,1-de]anthracen-7-yl)-10*H*-spiro[acridine-9,9'-fluorene] (DBA-SAF). An assistant dopant exhibited λ_{peak} of 455 nm in toluene solution (0.05mM), but that of DBA-SAF is 476 nm in 30 wt.% doped DBFPO host system with high Φ_{PLQY} ~ 0.94 (i.e., concentration quenching resistant). It originated from the electronic stabilization effect observed in the conventional CT-type molecule (**Figure 2**a).(*56*) To control FRET process from $S_{1,\,Assistant}$ (DBA-SAF) to $S_{1,Terminal}$ (*o*-Tol-*v*-DABNA-Me), the value of red-dashed spectral overlap integral (J_F) was calculated to be 6.71 × 10^{14} nm^4 M^{-1} cm^{-1} in **Figure 2**a, corresponding to the FRET radius (R_F) of 2.61 nm (see **Table S6**).

The steady-state PL profiles resemblance between binary composition (i.e., host-guest) and ternary one (i.e., HF architecture) supports that FRET process: $S_{1,\,Assistant} \rightarrow S_{1,Terminal}$ occurs dominantly, leading to a narrowed blue emission similar to the terminal dopant one (**Figure 2**b). Strikingly, given that the efficiency of FRET (Φ_{FRET}) resides at ~ 64% for the state-of-art *v*-DABNA-based HF system, (*9*) the proposed HF architecture with Φ_{FRET} of ~ 98% in this study (see **Figure S43**) utilizes the overshadowed benefits, particularly, color purity issue. In other words, an incomplete FRET arises, facing a tricky issue for the suppression of entailed emission spectrum from an assistant dopant although the low terminal doping ratio of 0.5 – 2.0 wt.% in HF system could prevent from the *v*-DABNA excimer quenching on the extent.

To grasp the whole picture of FRET-based HF system, the average distance between dopants (R') can be estimated by the following equation: (*57*) $R' = [(\text{molecular density in a film}) \times (\text{mol \% of the dopant in a film})]^{-1/3}$ wherein we used the film density (ρ) of 1.185 ± 0.001 g/cm^3 at converged 200 frames obtained from each MD simulation by running *NPT* ensemble. **Figure S44**a shows R' dependent with doping concentration for a series of *o*-Tol-*v*-DABNA-Me (terminal) and DBA-SAF (assistant), respectively. FRET-based quenching radius of *o*-Tol-*v*-DABNA-Me @ 3 wt.% doped DBFPO host film was 2.44 nm, corresponding to around 23 wt.% doping condition (i.e., $\Phi_{FRET\text{-}CQ,\,Terminal}$ of 50 %). To get clear spatial correlations in a ternary film, we derived the radial distribution function [R.D.F, g(*r*)] as a function of the center-of-mass (C.O.M) distance as shown in **Figure S44**b. The simulated ρ was 1.180 ± 0.001 g/cm^3, which is similar to those of binary films. Interestingly enough, we found that the estimated R' (1.4 nm) for DBA-SAF @ 30 wt.% in **Figure S44**a is well-matched with that of $R_{DBA\text{-}SAF:DBA\text{-}SAF}$ obtained from the first peak (1.2 nm). And the average intermolecular distance between DBFPO and DBA-SAF ($R_{DBFPO:DBA\text{-}SAF}$) is close enough to transfer excitons from host to assistant dopant in this EML composition. In conclusion, the value of $R_{DBA\text{-}SAF:\,o\text{-}Tol\text{-}v\text{-}DABNA\text{-}Me}$ was 1.28 nm, which strongly supports that FRET predominantly occurs from $S_{1,\,Assistant} \rightarrow S_{1,Terminal}$ in this ternary film in absence of concentration quenching effect from terminal dopants.

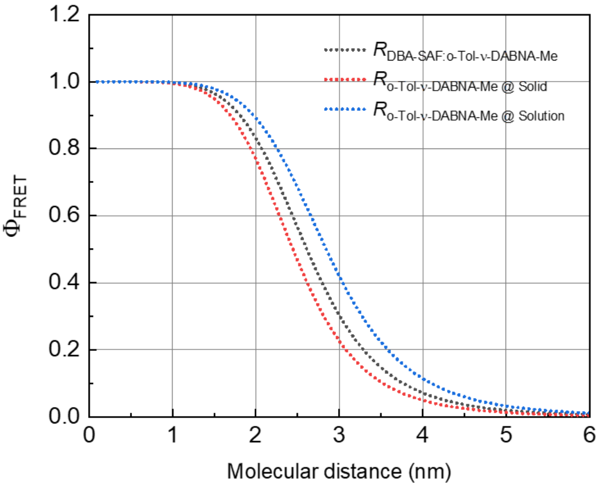

Figure S43. Φ_{FRET} as a function of molecular distance (r) for each system.

Table S6. Calculated FRET radius (R_F) in each individual system.

System	Type	J_F [nm^4 M^{-1} cm^{-1}]	Φ_D	R_F [nm]
DBFPO:DBA-SAF (30 wt. %)	Enegy transfer	6.71 × 10^{14}	0.94	2.61
DBFPO:*o*-Tol-*ν*-DABNA-Me (3 wt. %)	Self-quenching	4.29 × 10^{14}	0.99	2.44
o-Tol-*ν*-DABNA-Me (0.01 mM, Toluene)	Self-quenching	1.14 × 10^{15}	0.92	2.84

In this study, we used the equation: (*58*-*59*) $R_F^6 = \frac{9\ln 10}{128\pi^5 N_A}\frac{\kappa^2 \Phi_D}{n^4} J_F$ where N_A is Avogadro's number, κ is the dipole orientation factor ($0.845\sqrt{2/3}$), n is the index of refraction at the wavelength of maximum overlap (1.8). Φ_D is the PLQY of the donor in the absence of the acceptor, and J_F is the spectral overlap integral ($\int \overline{f_D}(\lambda)\varepsilon_A(\lambda)\,\lambda^4 d\lambda$) between the donor PL, $\overline{f_D}(\lambda)$ wherein the donor (DBA-SAF or o-Tol-ν-DAVNA-Me) emission spectrum is normalized to an area of 1, and the molar extinction coefficient of o-Tol-ν-DAVNA-Me [$\varepsilon_A(\lambda)$].

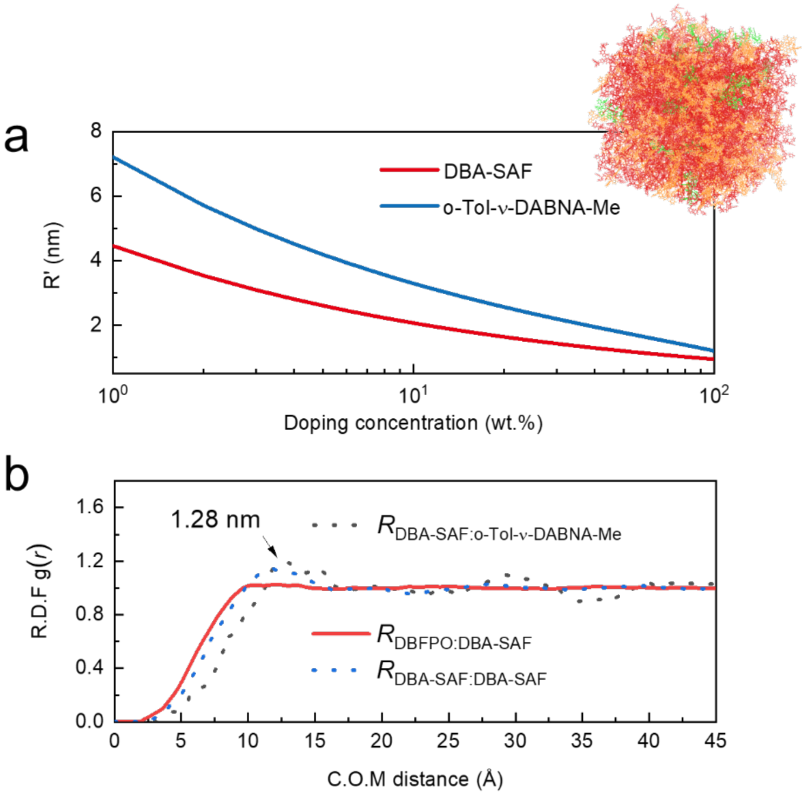

Figure S44. Intermolecular distances (a) An average intermolecular distance (*R*') versus doping concentration (wt.%). Inset: the equilibrated amorphous state for *o*-Tol-*ν*-DABNA-Me (3 wt.%):DBA-SAF (30 wt.%):DBFPO ternary film by MD simulation @ 100.0 ns (b) The radial distribution function (RDFs) for *o*-Tol-*ν*-DABNA-Me (3 wt.%):DBA-SAF (30 wt.%):DBFPO ternary film, as a function of C.O.M intermolecular distances (*R*).

Table S7. Condensed solid film models for *o*-Tol-*v*-DABNA-Me based system

Item (mean)	DBFPO	3 wt.%	5 wt.%	10 wt.%	20 wt.%	40 wt.%	Neat	HF
Density (g/cm^3)	1.188	1.185	1.180	1.174	1.156	1.129	1.024	1.180
$^aE_{coh.}$ (kcal/mol)	45.71	45.85	46.01	46.43	47.08	49.28	61.39	44.40
$^b\Delta H_{vap}$ (kcal/mol)	46.30	46.45	46.60	47.03	47.68	49.88	61.99	45.00
^{c}Vdw (kcal/mol)	-37.59	-37.93	-38.06	-38.59	-39.62	-42.60	-57.02	-38.27
Electrostatic (kcal/mol)	-8.72	-8.52	-8.55	-8.44	-8.05	-7.28	-4.98	-6.73

^{a}The cohesive energy (E_{coh}) is the energy of the periodic unit cell (E_{cell}) divided by the number of molecules in the cell (N = 1024), minus the weighted energy of a single average molecule (E_{mol}) in the gas phase, which can be obtained: $E_{coh} = \frac{E_{cell}}{N} - E_{mol}$. ^{b}The heat of vaporization (ΔH_{vap}) is calculated from the following equation: $\Delta H_{vap} =< |E_{cell} - \sum_i^{1024} E_i| >_{t=80-100\text{ ns}} + RT$.
^{C}Van der Waals interaction.

Table S8. Condensed solid film models for *v*-DABNA based system

Item (mean)	DBFPO	3 wt.%	5 wt.%	10 wt.%	20 wt.%	40 wt.%	Neat
Density (g/cm^3)	1.188	1.188	1.184	1.179	1.175	1.154	1.091
$^aE_{coh.}$ (kcal/mol)	45.71	46.11	46.21	46.75	48.24	51.15	67.15
$^b\Delta H_{vap}$ (kcal/mol)	46.30	46.70	46.80	47.35	48.83	51.74	67.75
^{c}Vdw (kcal/mol)	-37.59	-38.04	-38.27	-38.99	-40.65	-44.05	-61.55
Electrostatic (kcal/mol)	-8.72	-8.67	-8.53	-8.36	-8.19	-7.77	-6.20

^{a}The cohesive energy (E_{coh}) is the energy of the periodic unit cell (E_{cell}) divided by the number of molecules in the cell (N = 1024), minus the weighted energy of a single average molecule (E_{mol}) in the gas phase, which can be obtained: $E_{coh} = \frac{E_{cell}}{N} - E_{mol}$. ^{b}The heat of vaporization (ΔH_{vap}) is calculated from the following equation: $\Delta H_{vap} =< |E_{cell} - \sum_i^{1024} E_i| >_{t=80-100\text{ ns}} + RT$.
^{C}Van der Waals interaction.

XI. Comparison Study for o-Tol-ν-DABNA-Me vs. ν-DABNA

Photophysical Study We calculated the Huang-Rhys (HR) factor of unmodified *ν*-DABNA (control) as shown in **Figure S45**. The total factor HR factor for all the vibrational modes involved ($= \sum_{i=1}^{414} S_i$) is estimated to be as small as 0.67, which shows little difference compared to that of *o*-Tol-*ν*-DABNA-Me (1.07). The reorganization energies of *ν*-DABNA for its S_1 (λ_S) and T_1 (λ_T) states have been estimated to be as low as 0.03 and 0.04 eV, respectively, from the potential energy surface curves. For a clear comparison between *o*-Tol-*ν*-DABNA-Me and *ν*-DABNA, we have tabulated the adiabatic energies for excited states and SOCME, as shown in **Table S9**. Based on the TD-DFT results, it is clear that PPGs not only effectively restrict its major non-radiative recombination channel by the steric hindrance, but also slightly de-stabilize the excited electronic state [$S_{1\ (Adia)}$; 2.87 eV (*ν*-DABNA) → 2.88 eV (*o*-Tol-*ν*-DABNA-Me)], thus shifting the emission peak toward the shorter wavelength. (**Table S9**) Although the modification of PPGs could be prone to alter the molecular vibrational behavior in such a way that Stokes shift and FWHM increases, (*8*) our design strategy of PPGs retain a small HR-factor on the *ν*-DABNA backbone, keeping all the beneficial characteristics of *ν*-DABNA such as narrow FWHM and small Stokes shift (See emission and absorption spectra shown in **Figure S46**) while reducing excimer formation and shifting the peak wavelength toward deeper blue.

Table S9. Calculated adiabatic energies for excited states and SOCME for *ν*-DABNA (Control)

$S_{1(Adia)}$[a]	$T_{1(Adia)}$[a]	$T_{1(Adia)}$[b]	HOMO[c]	LUMO[c]	[d]$H_{SO,T1}$	[d]$H_{SO,T2}$	[d]$H_{SO,T3}$
2.87 eV	2.52 eV	2.52 eV	-4.84 eV	-1.45 eV	0.01 cm^{-1}	0.04 cm^{-1}	0.04 cm^{-1}

[a]Optimized B3LYP/def2-SVP at TD-DFT level. [b]Triplet geometry obtained from UDFT method. [c]at optimized ground state (B3LYP/def2-SVP). [d]SOCME values between S_1 and T_n (n = 3) at the T_1 geometry accessed by spin-relaxed open-shell optimization. The calculation based on B3LYP/ZORA basis set at the scalar relativistic level (i.e., pSOC-TDDFT within TDA). *ν*-DABNA

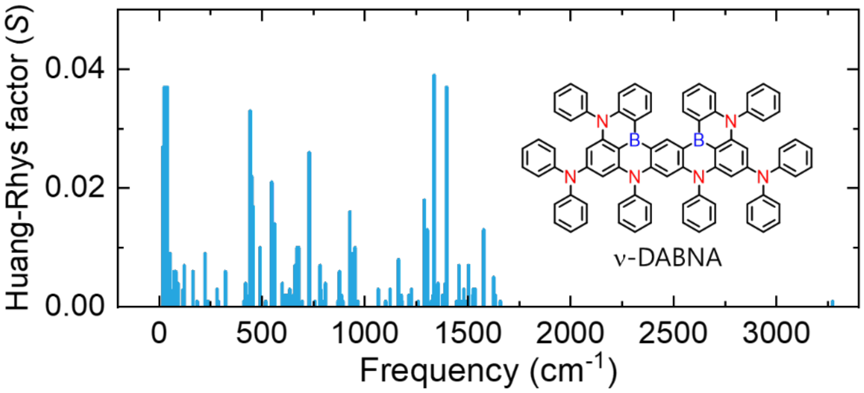

Figure S45. The Huang-Rhys (HR) factor (S_i) factorized into reorganization energy (λ_S) for *ν*-DABNA system. To obtain the equilibrium geometries and vibration frequencies in each electronic state, we performed a series of DFT calculations at B3LYP/def2-SVP level. The total HR factor for all the vibrational modes involved ($= \sum_{i=1}^{420} S_i$) is estimated to be as small as 0.67

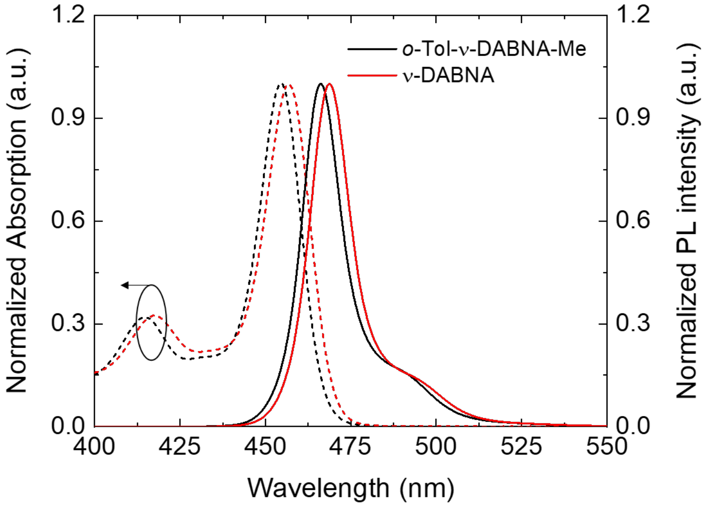

Figure S46. Absorption spectra of *ν*-DABNA (red) and *o*-Tol-*ν*-DABNA-Me (black) in toluene solution (0.05 mM) and the fluorescence PL spectra measured at a toluene solution (conc at 0.05 mM). The Stoke shift for *ν*-DABNA (o-Tol-*ν*-DABNA-Me) is 0.065 (0.065) eV, respectively.

Molecular Anisotropy We conducted experimental and theoretical studies to characterize the transition dipole orientation (TDO) of *ν*-DABNA. First of all, theoretical calculation shows that the transition dipole moment (TDM) of *ν*-DABNA is also in the plane of the DABNA ribbon along the long axis (see **Figure S47**), similar to our proposed molecule (*o*-Tol-*ν*-DABNA-Me). Furthermore, from an angle-dependent characterization of *p*-polarized PL profile (refer to **Figure S48**), Θ_h of *ν*-DABNA doped in DBFPO host film is estimated to be also very high, at 0.945. One should note that, with the proposed *o*-Tol-*ν*-DABNA-Me, we are not trying to make a "better-in-all-aspects" emitter than *ν*-DABNA. Rather, our purpose is to solve the excimer-formation issue while keeping the beneficial characteristics of *ν*-DABNA as much as possible so that we can unlock the full potential of *ν*-DABNA and its derivatives.

Please keep in mind that excimer formations even at a relatively mild doping concentration (i.e., 3 wt.%) could not be suppressed in *ν*-DABNA without the help of the proposed fine-tuned PPGs design; if excimer formation persisted, even though *ν*-DABNA has a highly horizontal TDM orientation yielding a high outcoupling efficiency, (i) the overall EQE might not necessarily be high due to the reduced PLQY (The measured PLQY for a film embedded in BN and HF-type for *ν*-DABNA was 0.63 and 0.62, respectively.), and furthermore, the device would be subject to the reduction in color purity.

The effectiveness of the proposed PPGs in suppressing excimer formation is further confirmed in **Figure S49** that shows the steady-state PL spectra for *o*-Tol-*ν*-DABNA-Me and *ν*-DABNA. In summary, the PPGs proposed hereby maintain the molecular arrangement to be highly anisotropic through a series of dipole-induced van der Waals (Vdw) interactions between our target molecule and surrounding host molecules, and mitigate excimer

formation issue, associated with Vdw interaction between *v*-DABNA derivative itself. It is noteworthy that the excimer-formation has been one of the most serious bottlenecks of *v*-DABNA that holds back its practical use in the field.

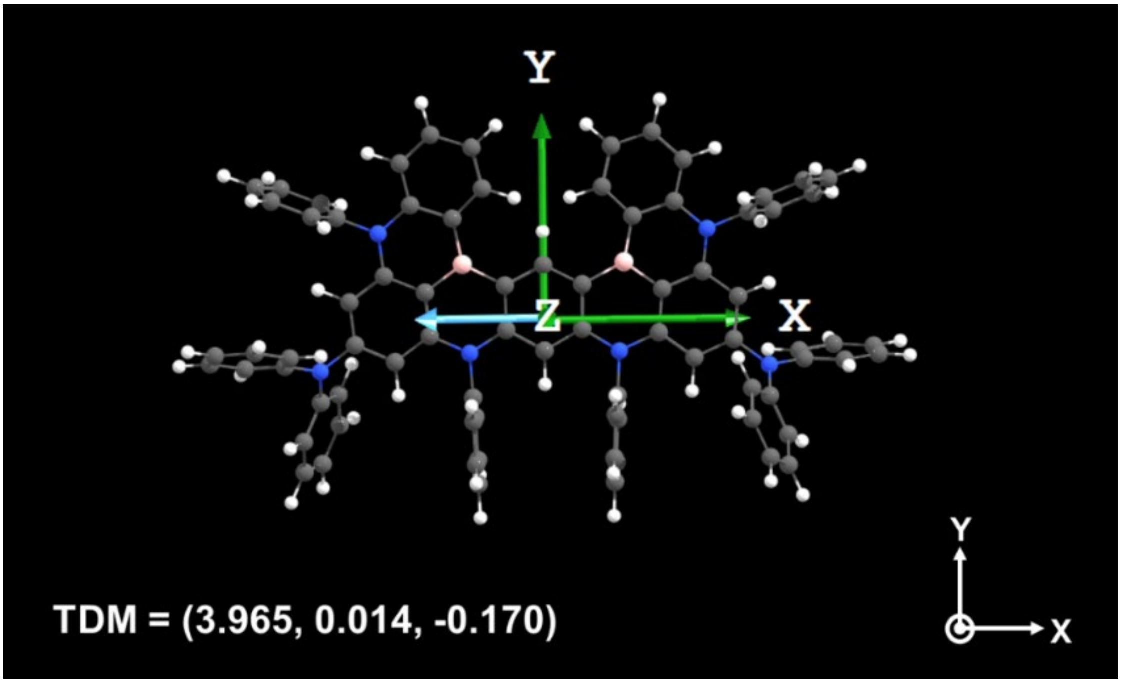

Figure S47. The transition dipole moment (TDM) vector for *v*-DABNA from the electronic transition: $S_1 \rightarrow S_0$. The single-point calculation is done by B3LYP/def2-SVP @ TD-DFT level reference from the optimized excited singlet state (S_1).

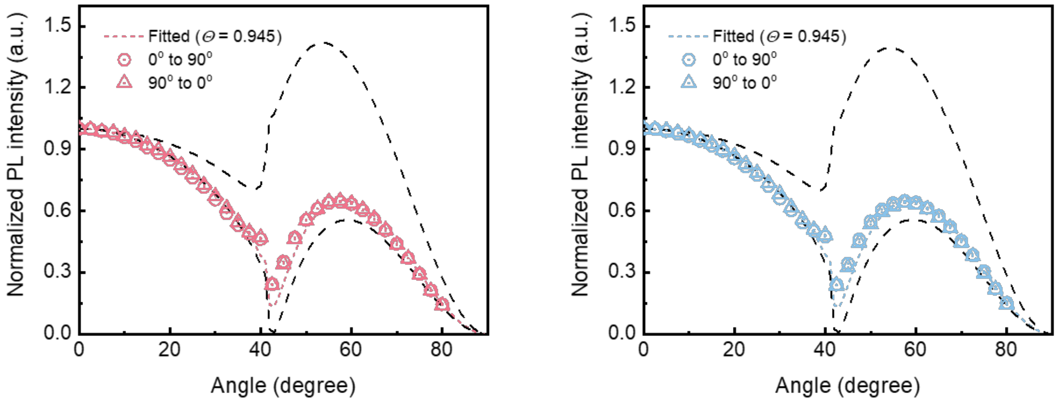

Figure S48. Angular-dependent *p*-polarized PL profiles for *v*-DABNA (3 wt.% doped) DBFPO host [left, Binary film, 50 nm] and DBFPO:DBA-SAF (30 wt. %): *v*-DABNA (3 wt. %) [right, the ternary film, 50 nm], respectively.

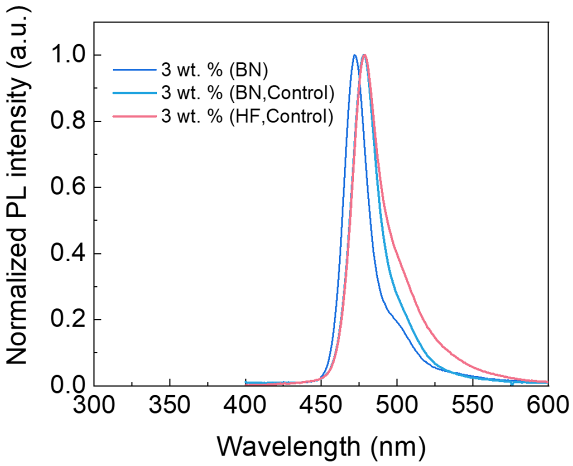

Figure S49. Steady-state PL spectra for *o*-Tol-*ν*-DABNA-Me (3 wt.% doped DBFPO host, BN), ν-DABNA (3 wt.% doped DBFPO host, BN), and HF system for DBFPO:DBA-SAF (30 wt. %):ν-DABNA (3 wt. %).

Device Performance We conducted a 1-to-1 comparison between *ν*-DABNA and the proposed *o*-Tol-*ν*-DABNA-Me using the same EML type (i.e., BN- and HF-type) and dopant concentration (3 wt. %). The detailed information on the device performances (*o*-Tol-*ν*-DABNA-Me vs. *ν*-DABNA) is summarized in **Table S10** and **Figure. S50**. In summary, the devices with the proposed *o*-Tol-*ν*-DABNA-Me were found to be superior to those made of *ν*-DABNA in terms of both EQE and CIE y-coordinate, whether the EML composition was binary (BN)-type or for hyperfluorescent (HF)-type. Considering that the EQE and CIE y-coordinate of BN-type OLEDs based on low-concentration *ν*-DABNA (1 wt.%) are comparable to those of BN-type OLEDs based on 3 wt.% *o*-Tol-*ν*-DABNA-Me, (*9*) the lower EQE (26.0 %) and increased y-coordinate (0.19) in the OLEDs with 3 wt.% v-DABNA well illustrate the adverse effect of concentration-induced excimer formation in *ν*-DABNA such as lowered PLQY and increased longer-wavelength side-peak emission. Note that the measured PLQY for the 3 wt.% film for *ν*-DABNA was 0.63 for BN-type EM and 0.62 for HF-type EML. To clarify our assertion, we carried out the full angular characterization of *ν*-DABNA based OLEDs without Lambertian simplification (**Figures. S51** and **S52**). (*60*) EQE obtained in this way well corresponds to the value predicted with optical simulation done with the PLQY values mentioned above. (**Figure. S53**).

The benefit of the proposed approach is even clearer in the HF-case. It is worthwhile to re-emphasize that the results presented in this work demonstrate a clear improvement over the *ν*-DABNA-based HF type OLED in almost all the essential categories (e.g., λ_{EL}, EQE, λ_{FWHM}, and color purity) including the roll-off. This improvement was made since the proposed *o*-Tol-*ν*-DABNA-Me allows for higher end-emitter concentrations with less concerns about excimer formation. As a result, HF OLEDs based on o-Tol-v-DABNA-Me has a considerable advantage over *ν*-DABNA-based HF OLED in terms of efficiency roll-off (refer to the peak-normalized EQE curves *vs*. *J* in, **Figure. S54**)

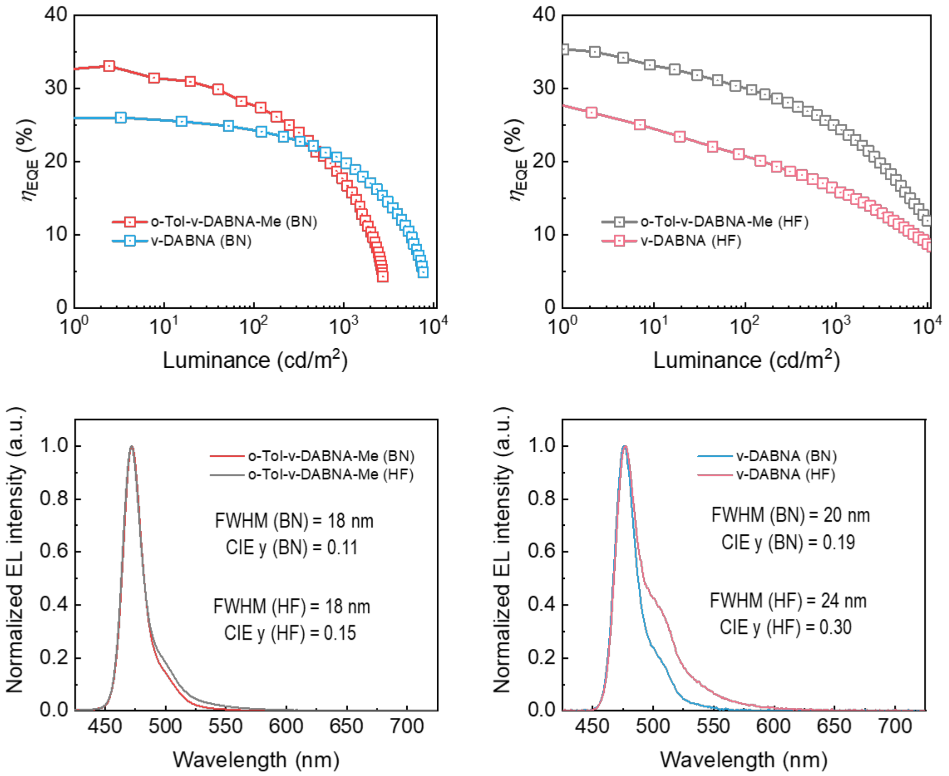

Figure S50. *EQE-L* curves and normalized EL spectra for a series of tested devices at normal direction.

Table S10. The key characteristics of OLEDs based on *o*-Tol-*ν*-DABNA-Me vs. ν-DABNA (Control)

**Device**[a]	**EML Type/ Dopant conc. (wt.%)**	**λ_{EL} (nm)**	**EQE (%)**[b]	**λ_{FWHM} (nm)**	**CIE (x, y)**
Dev. A	BN/3.0	472	33.1/17.7	18	(0.11, **0.12**)
Cont. A	BN/3.0	476	26.0/20.0	20	(0.10, **0.19**)
Dev. D	HF/3.0	472	35.4/25.0	18	(0.12, **0.15**)
Cont. D	HF/3.0	477	27.7/16.1	24	(0.12, **0.30**)

[a]ITO (70 nm)/MoO_3 (4 nm)/TAPC (40 nm)/TCTA (10 nm)/mCP (10 nm)/EML (25 nm)/DBFPO (5 nm)/TPBi (35 nm)/LiF (1 nm)/Al (100 nm) wherein the binary (BN) film (3.0 wt.% doped DBFPO host) for **Dev. A, Cont. A** and ternary film (3.0 wt.% *o*-Tol-*ν*-DABNA-Me or *ν*-DABNA: 30 wt.% DBA-SAF: DBFPO host) for **Dev. D**, **Cont. D**, respectively. [b]External quantum efficiency: maximum, then values at 1,000 cd/m^2.

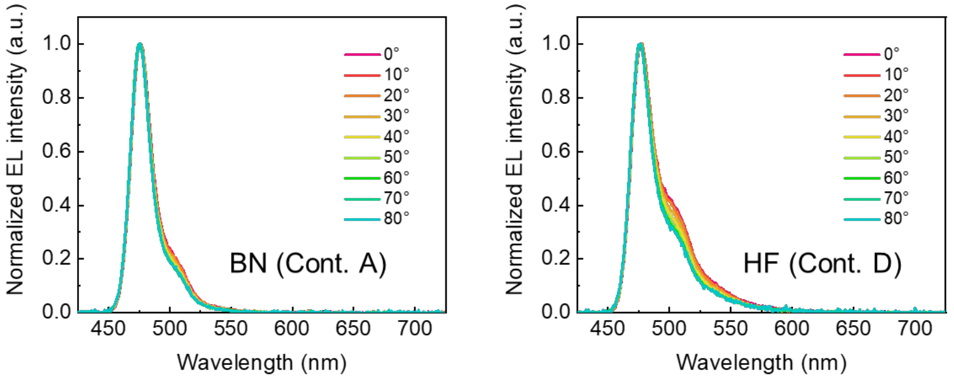

Figure S51. Angular EL spectra for a series of tested devices in this study (in steps of 10°).

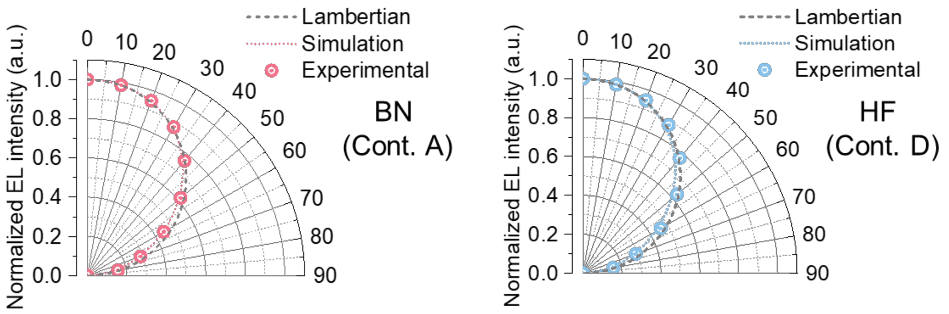

Figure S52. The angle dependent EL emission pattern of a series of tested devices.

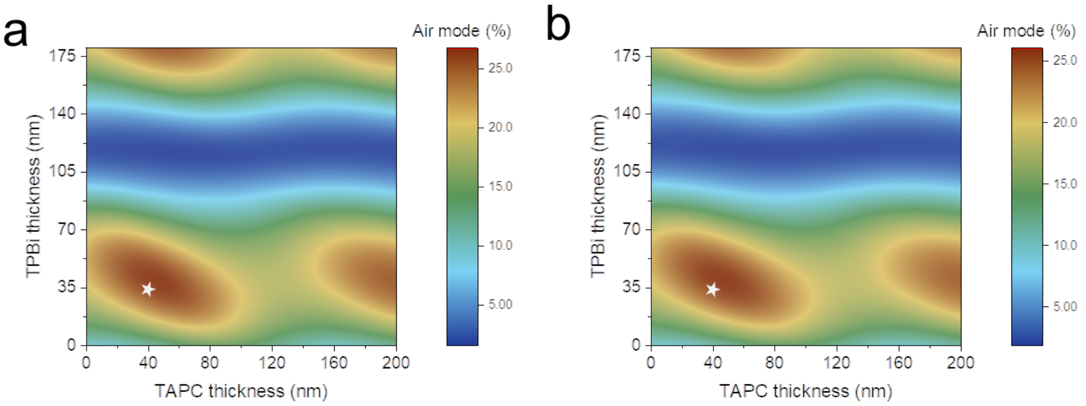

Figure S53. Maximum EQE vs. ETL/HTL thicknesses calculated for (a) **BN (Cont. A)** and (b) **HF (Cont. D)** with the proposed device architecture. [PLQY: 0.63(BN) and 0.62(HF), Θ_h:0.945 for BN and HF. Calculation was based on the so-called 'CPS' model. (*61*) The "stars" on the plot indicate the device geometries taken in this study.

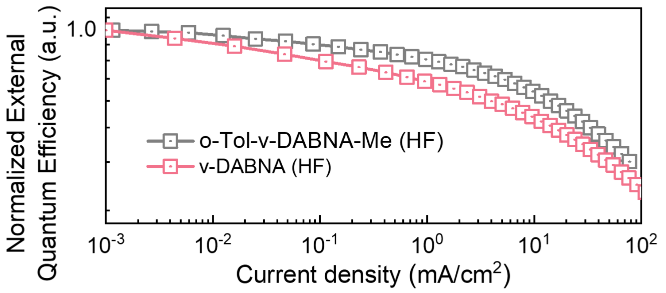

Figure S54. *J-EQE* curves for HF architecture with *o*-Tol-*v*-DABNA-Me and *v*-DABNA in this study. EQE is normalized to the maximum values for each of the cases to better compare the roll-off behavior.

Photostability of *v*-DABNA and its derivative We conducted further investigations on the photostability of *o*-Tol-*v*-DABNA-Me in comparison to that of the control, *v*-DABNA. To this end, we prepared the binary thin film (3 wt.% doped on DBFPO host) deposited on a quartz substrate for UV irradiation. Based on the result of the PL reliability test (refer to **Figure S55**), it is evident that the proposed *o*-Tol-*v*-DABNA-Me has photostability that is comparable to or slightly better than *v*-DABNA.

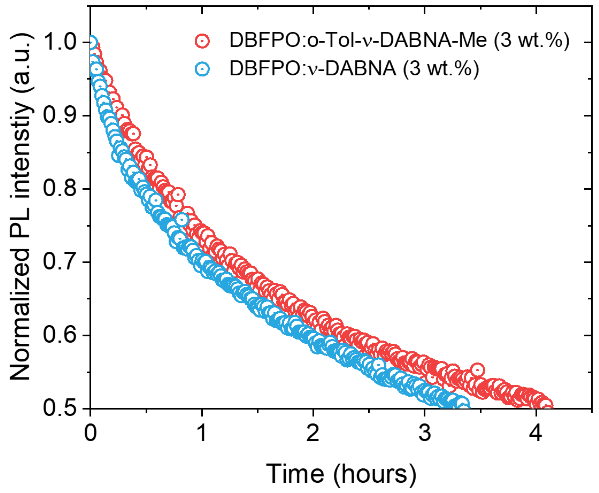

Figure S55. The UV irradiation PL reliability test for *v*-DABNA and its under the excitation power of 5 mW/cm^2 at the wavelength of 340 ± 5 nm.

REFERENCES AND NOTES

1. H. Uoyama, K. Goushi, K. Shizu, H. Nomura, C. Adachi, Highly efficient organic light-emitting diodes from delayed fluorescence. *Nature* **492**, 234–238 (2012).

2. S. O. Jeon, K. H. Lee, J. S. Kim, S. -G. Ihn, Y. S. Chung, J. W. Kim, H. Lee, S. Kim, H. Choi, J. Y. Lee, High-efficiency, long-lifetime deep-blue organic light-emitting diodes. *Nat. Photon.* **15**, 208–215 (2021).

3. S. Hirata, Y. Sakai, K. Masui, H. Tanaka, S. Y. Lee, H. Nomura, N. Nakamura, M. Yasumatsu, H. Nakanotani, Q. Zhang, K. Sizu, H. Miyazaki, C. Adachi, Highly efficient blue electroluminescence based on thermally activated delayed fluorescence. *Nat. Mater.* **14**, 330–336 (2015).

4. H. Tanaka, S. Oda, G. Ricci, H. Gotoh, K. Tabata, R. Kawasumi, D. Beljonne, Y. Olivier, T. Hatakeyama, Hypsochromic shift of multiple-resonance-induced thermally activated delayed fluorescence by oxygen atom incorporation. *Angew. Chem. Int. Ed.* **60**, 17910–17914 (2021).

5. T. Hatakeyama, K. Shiren, K. Nakajima, S. Nomura, S. Nakatsuka, K. Kinoshita, J. Ni, Y. Ono, T. Ikuta, Ultrapure blue thermally activated delayed fluorescence molecules: Efficient HOMO–LUMO separation by the multiple resonance effect. *Adv. Mater.* **28**, 2777–2781 (2016).

6. Y. Kondo, K. Yoshiura, S. Kitera, H. Nishi, S. Oda, H. Gotoh, Y. Sasada, M. Yanai, T. Hatakeyama, Narrowband deep-blue organic light-emitting diode featuring an organoboron-based emitter. *Nat. Photon.* **13**, 678–682 (2019).

7. A. Pershin, D. Hall, V. Lemaur, J. -C Sancho-Garcia, L. Muccioli, E. Zysman-Colman, D. Belijonne, Y. Olivier, Highly emissive excitons with reduced exchange energy in thermally activated delayed fluorescent molecules. *Nat. Commun.* **10**, 597 (2019).

8. K. Stavrou, A. Danos, T. Hama, T. Hatakeyama, A. Monkman, Hot vibrational states in a high-performance multiple resonance emitter and the effect of excimer quenching on organic light-emitting diodes. *ACS Appl. Mater. Interfaces* **13**, 8643–8655 (2021).

9. C. -Y. Chan, M. Tanaka, Y. -T. Lee, Y. -W. Wong, H. Nakanotani, T. Hatakeyama, C. Adachi, Stable pure-blue hyperfluorescence organic light-emitting diodes with high-efficiency and narrow emission. *Nat. Photon.* **15**, 203–207 (2021).

10. H. J. Cheon, Y. -S. Shin, N. -H. Park, J. -H. Lee, Y. -H. Kim, Boron-based multi-resonance TADF emitter with suppressed intermolecular interaction and isomer formation for efficient pure blue OLEDs. *Small* **18**, 2107574 (2022).

11. P. T. Ruhoff, M. A. Ratner, Algorithms for computing Franck–Condon overlap integrals. *Int. J. Quantum.* **77**, 383–392 (2000).

12. A. Toniolo, M. Persico, Efficient calculation of Franck–Condon factors and vibronic couplings in polyatomics. *Int. J. Quantum.* **22**, 968–975 (2001).

13. H. S. Kim, J. Y. Lee, S. Shin, W. Jeong, S. H. Lee, S. Kim, J. Lee, M. C. Suh, S. Yoo, Enhancement of reverse intersystem crossing in charge-transfer molecule through internal heavy atom effect. *Adv. Funct. Mater.* **31**, 2104646 (2021).

14. T. E. Sharp, H. M. Rosenstock, Franck–Condon factors for polyatomic molecules. *J. Chem. Phys.* **41**, 3453–3463 (1964).

15. P. K. Samanta, D. Kim, V. Coropceanu, J.-L. Brédas, Up-conversion intersystem crossing rates in organic emitters for thermally activated delayed fluorescence: Impact of the nature of singlet vs triplet excited states. *J. Am. Chem. Soc.* **139**, 4042–4051 (2017).

16. Q. Peng, D. Fan, Y. Yi, Y. Niu, D. Wang, Z. Shuai, Theoretical study of conversion and decay processes of excited triplet and singlet states in a thermally activated delayed fluorescence molecule. *J. Phys. Chem. C* **121**, 13448–13456 (2017).

17. X. -K. Chen, D. Kim, J.-L. Brédas, Thermally activated delayed fluorescence (TADF) path toward efficient electroluminescence in purely organic materials: Molecular level insight. *Acc. Chem. Res.* **51**, 2215–2224 (2018).

18. C. Lu, C. Wu, D. Ghoreishi, W. Chen, L. Wang, W. Damm, G. A. Ross, M. K. Dahlgren, E. Russell, C. D. Von Bargen, R. Abel, R A. Friesner, E. D. Harder, OPLS4: Improving force field accuracy on challenging regimes of chemical space. *J. Chem. Theory Comput.* **17**, 4291–4300 (2021).

19. D. R. Evans, H. S. Kwak, D. J. Giesen, A. Goldberg, M. D. Halls, Estimation of charge carrier mobility in amorphous organic materials using percolation corrected random-walk model. *Org. Electron.* **29**, 50–56 (2016).

20. A. D. Bochevarov, Jaguar: A high-performance quantum chemistry software program with strengths in life and materials sciences. *Int. J. Quantum Chem.* **113**, 2110–2142 (2013).

21. S. T. Schneebeli, A. D. Bochevarov, R. A. Friesner, Parameterization of a B3LYP specific correction for noncovalent interactions and basis set superposition error on a gigantic data set of CCSD(T) quality noncovalent interaction energies. *J. Chem. Theory. Comput.* **7**, 658–668 (2011).

22. C. -K. Moon, K. -H. Kim, J. -J. Kim, Unraveling the orientation of phosphors doped in organic semiconducting layers. *Nat. Commun.* **8**, 791 (2017).

23. K. Masui, H. Nakanotani, C. Adachi, Analysis of exciton annihilation in high-efficiency sky-blue organic light-emitting diodes with thermally activated delayed fluorescence. *Org. Electron.* **14**, 2721–2726 (2013).

24. T. Furukawa, H. Nakanotani, M. Inoue, C. Adachi. Dual enhancement of electroluminescence efficiency and operational stability by rapid upconversion of triplet excitons in OLEDs. *Sci. Rep.* **5**, 8429 (2015).

25. C. Baleizão, M. N. Berberan-Santos, Thermally activated delayed fluorescence as a cycling process between excited singlet and triplet states: Application to the fullerenes. *J. Chem. Phys.* **126**, 204510 (2007).

26. B. Yurash, H. Nakanotani, Y. Olivier, D. Beljonne, C. Adachi, T. -Q. Nguyen, Photoluminescence quenching probes spin conversion and exciton dynamics in thermally activated delayed fluorescence materials. *Adv. Mater.* **31**, 1804490 (2019).

27. H. Lim, H. J. Cheon, S.-J. Woo, S.-K. Kwon, Y.-H. Kim, J.-J. Kim, Highly efficient deep-blue OLEDs using a TADF emitter with a narrow emission spectrum and high horizontal emitting dipole ratio. *Adv. Mater.* **32**, 2004083 (2020).

28. M. Numata, T. Yasuda, C. Adachi, High efficiency pure blue thermally activated delayed fluorescence molecules having 10H-phenoxaborin and acridan units. *Chem. Commun.* **51**, 9443–9446 (2015).

29. G. M. Sheldrick, A short history of SHELX. *Acta Crystallogr.* **64**, 112–122 (2008).

30. G. M. Sheldrick, Crystal structure refinement with SHELXL. *Acta Crystallogr.* **71**, 3–8 (2015).

31. A. D. Becke, A new mixing of Hartree–Fock and local density-functional theories. *J. Chem. Phys.* **98**, 1372–1377 (1993).

32. C. Lee, W. Yang, R. G. Parr, Development of the Colle-Salvetti correlation-energy formula into a functional of the electron density. *Phys. Rev. B* **37**, 785–789 (1988).

33. F. Weigend, R. Ahlrichs, Balanced basis sets of split valence, triple zeta valence and quadruple zeta valence quality for H to Rn: Design and assessment of accuracy. *Phys. Chem. Chem. Phys.* **7**, 3297–3305 (2005).

34. M. E. Casida, M. Huix-Rotllant, Progress in time-dependent density-functional theory. *Annu. Rev. Phys. Chem.* **63**, 287–323 (2012).

35. J. P. Perdew, K Burke, M. Ernzerhof, Generalized gradient approximation made simple. *Phys. Rev. Lett.* **77**, 3865–3868 (1996).

36. O. A. Vydrov, G. E. Scuseria, Assessment of a long-range corrected hybrid functional. *J. Chem. Phys.* **125**, 234109 (2006).

37. J.-D. Chai, M. Head-Gordon, Systematic optimization of long-range corrected hybrid density functionals. *J. Chem. Phys.* **128**, 084106 (2008).

38. A. D. Becke, Density-functional thermochemistry. V. Systematic optimization of exchange-correlation functionals. *J. Chem. Phys.* **17**, 8554 (1997).

39. T. Yanai, D. P. Tew, N. C. Handy, A new hybrid exchange–correlation functional using the Coulomb-attenuating method (CAM-B3LYP). *Chem. Phys. Lett.* **393**, 51–57 (2004).

40. H. Sun, C. Zhong, J.-L. Brédas, Reliable prediction with tuned range-separated functionals of the singlet–Triplet gap in organic emitters for thermally activated delayed fluorescence. *J. Chem. Theory. Comput.* **11**, 3851–3858 (2015).

41. M. E. Casida, Correlated optimized effective-potential treatment of the derivative discontinuity and of the highest occupied Kohn-Sham eigenvalue: A Janak-type theorem for the optimized effective-potential model. *Phys. Rev. B* **59**, 4694–4698 (1999).

42. K. Bhattacharyya, Can TDDFT render the electronic excited states ordering of Azine derivative? A closer investigation with DLPNO-STEOM-CCSD. *Chem. Phys. Lett.* **779**, 138827 (2021).

43. A. K. Dutta, M. Nooijen, F. Neese, R. Izsák, Exploring the accuracy of a low scaling similarity transformed equation of motion method for vertical excitation energies. *J. Chem. Theory. Comput.***14**, 72–91 (2018).

44. R. L. Martin, Natural transition orbitals. *J. Chem. Phys.* **118**, 4775–4777 (2003).

45. F. Wang, T. Ziegler, E. van Lenthe, S. J. van Gisbergen, E. J. Baerends, The calculation of excitation energies based on the relativistic two-component zeroth-order regular approximation and time-dependent density-functional with full use of symmetry. *J. Chem. Phys.* **122**, 204103 (2005).

46. E. van Lenthe, E. J. Baerends, J. G. Snijders, Relativistic regular two-component hamiltonians. *J. Chem. Phys.* **99**, 4597–4610 (1993).

47. E. van Lenthe, E. J. Baerends, J. G. Snijders, Relativistic total energy using regular approximations. *J. Chem. Phys.* **101**, 9783–9792 (1994).

48. K. G. Dyall, Relativistic and nonrelativistic finite nucleus optimized double zeta basis sets for the 4p, 5p and 6p elements. *Theor. Chem. Acc.* **99**, 366–371 (1998).

49. M. J. Frisch, G. W. Trucks, H. B. Schlegel, G. E. Scuseria, M. A. Robb, J. R. Cheeseman, G. Scalmani, V. Barone, G. A. Petersson, H. Nakatsuji, X. Li, M. Caricato, A. V. Marenich, J. Bloino, B. G. Janesko, R. Gomperts, B. Mennucci, H. P. Hratchian, J. V. Ortiz, A. F. Izmaylov, J. L. Sonnenberg, D. Williams-Young, F. Ding, F. Lipparini, F. Egidi, J. Goings, B. Peng, A. Petrone, T. Henderson, D. Ranasinghe, V. G. Zakrzewski, J. Gao, N. Rega, G. Zheng, W. Liang, M. Hada, M. Ehara, K. Toyota, R. Fukuda, J. Hasegawa, M. Ishida, T. Nakajima, Y. Honda, O. Kitao, H. Nakai, T. Vreven, K. Throssell, J. A. Montgomery Jr., J. E. Peralta, F. Ogliaro, M. J. Bearpark, J. J. Heyd, E. N. Brothers, K. N. Kudin, V. N. Staroverov, T. A. Keith, R. Kobayashi, J. Normand, K. Raghavachari, A. P. Rendell, J. C. Burant, S. S. Iyengar, J. Tomasi, M. Cossi, J. M. Millam, M. Klene, C. Adamo, R. Cammi, J. W. Ochterski, R. L. Martin, K. Morokuma, O. Farkas, J. B. Foresman, D. J. Fox, Gaussian 16 Revision C.01, Gaussian Inc., 2016, p. 16.

50. Schrödinger Release 2021-4: Jaguar, Schrödinger LLC, 2021.

51. F. Neese, The ORCA program system, *Wiley Interdiscip. Rev. Comput. Mol. Sci.* **2**, 73–78 (2012).

52. T. Lu, F. Chen, Multiwfn: A multifunctional wavefunction analyzer. *J. Comput. Chem.* **33**, 580–592 (2012).

53. C. Lu, C. Wu, D. Ghoreishi, W. Chen, L. Wang, W. Damm, G. A. Ross, M. K. Dahlgren, E. Rusell, C. D. V. Bargen, R. Abel, R. A. Friesner, E. D. Harder, OPLS4: Improving force field accuracy on challenging regimes of chemical space. *J. Comput. Chem.* **17**, 4291–4300 (2021).

54. K. Goushi, K. Yoshida, K. Sato, C. Adachi, Organic light-emitting diodes employing efficient reverse intersystem crossing for triplet-to-singlet state conversion. *Nat. Photon.* **6**, 253–258 (2012).

55. N. Aizawa, Y. Harabuchi, S. Maeda, Y. -J. Pu, Kinetic prediction of reverse intersystem crossing in organic donor–acceptor molecules. *Nat. Commun.* **11**, 3909 (2020).

56. H. S. Kim, S.-R. Park, M. C. Suh, Concentration quenching behavior of thermally activated delayed fluorescence in a solid film. *J. Phys. Chem. C* **121**, 13986–13997 (2017).

57. Y. Kawamura, J. Brooks, J. J. Brown, H. Sasabe, C. Adachi, Intermolecular interaction and a concentration-quenching mechanism of phosphorescent Ir(III) complexes in a solid film. *Phys. Rev. Lett.* **96**, 017404 (2006).

58. T. Forster, 10th Spiers memorial lecture. Transfer mechanisms of electronic excitation. *Discuss. Faraday Soc.* **27**, 7–17 (1959).

59. H. S. Kim, S. H. Lee, J. Y. Lee, S. Yoo, M. C. Suh, Mitigating the trade-off between triplet harvesting and roll-off by opening a Dexter-type channel in OLEDs. *J. Phys. Chem. C* **123**, 18283–18293 (2019).

60. S. -H. Jeong, J. Park, T. -H. Han, F. Zhang, K. Zhu, J. S. Kim, M. -H. Park, M. O. Reese, S. Yoo, T. -W. Lee, Characterizing the efficiency of perovskite solar cells and light-emitting diodes. *Joule* **4**, 1206–1235 (2020).

61. R. R. Chance, A. Prock, R. Silbey, Molecular fluorescence and energy transfer near metal interfaces. *Adv. Chem. Phys.* **37**, 1–65 (1978).
